# Introducing and utilizing innovative technologies in health care systems: a country comparison for peripheral drug-eluting stents in Germany and the USA

**DOI:** 10.3389/fpubh.2025.1488091

**Published:** 2025-06-19

**Authors:** Susanne Felgner, Helene Eckhardt, Marie Dreger, Dimitra Panteli, Cornelia Henschke

**Affiliations:** ^1^Department of Health Care Management, Institute of Technology and Management, Technische Universität Berlin, Berlin, Germany; ^2^Institute of Gender in Medicine, Charité – Universitätsmedizin Berlin, Berlin, Germany; ^3^Institute of General Practice and Interprofessional Care, University Hospital Tübingen, Tübingen, Germany

**Keywords:** medical device, innovation, evidence, decision-making, drug-eluting stent, safety, Germany, USA

## Abstract

Drug-eluting stents in the upper leg (DES-UL) are used to treat diseases of the peripheral vessels that are associated with an increased risk of cardiovascular events and are prevalent in industrialized countries such as Germany and the USA. Innovative technologies like DES-UL can bring great benefits to patients, possibly representing the only treatment option. However, they also entail risks since reliable evidence on efficacy/effectiveness and safety are often not available at the beginning of products' life cycles. The aim of the study is to examine utilization of DES-UL in German and US-American hospitals and the development of evidence on efficacy/effectiveness and safety for DES-UL over time. To identify evidence, we conducted a systematic literature search in four biomedical databases (2006–2022) for articles on clinical trials that we categorized by predefined characteristics, including studies' level of evidence (LoE) and population sizes, and the articles' conclusions regarding the technology's efficacy/effectiveness and safety clustered “positive”, “indecisive”, “neutral”, or “negative”. Additionally, we searched for clinical trial registry entries, HTA reports, clinical guidelines, safety notices & recalls, market approval dates, and financing instruments. The utilization of DES-UL was operationalized by annual hospital case numbers. We identified a total of 2,724 publications, of which 123 remained relevant after title/abstract and full text screening. In the early phase of the observation period of DES-UL utilization, the evidence development is characterized by a few articles on studies of low LoE and small population studies. Over time, the body of evidence expands, and articles on studies of high LoE (e.g., RCTs) and larger population sizes were published. Overall, articles with “positive” (n = 41) and “indecisive” (n = 58) conclusions predominate, with especially “positive” conclusions pointing to the efficacy/effectiveness and safety of DES-UL. Overall, utilization of DES-UL in hospitals increased in both Germany and the USA, although not uniformly across all years. An influence of various events on the case numbers' development can be assumed. Health policy makers must ensure that efficacy/effectiveness and safety of technologies are evaluated appropriately. Therefore, robust evidence should be generated and made accessible to clinical and health decision-makers in a timely manner and promptly reflected in clinical guidelines.

## 1 Introduction

Innovative technologies in medicine, including medical devices, play a crucial role for patients, health care professionals and health care systems (1–3). They can contribute to the improvement of diagnosis, treatment, and prevention of diseases (4). Furthermore, innovative medical devices enable new approaches in medicine, such as surgical procedures, and minimally invasive surgical procedures that require fewer invasive interventions and can lead to shorter recovery times and fewer complications, significantly improving quality of life (QoL) and increasing life expectancy of patients (5, 6). A key aspect of innovative medical technologies is their ability to enhance the efficiency and accuracy of medical procedures. By using new effective technologies, diagnoses can be made faster and more accurately, which can lead to earlier treatment and a better prognosis (4). These advantages of innovative technologies underline the need for adequate coverage to allow access for patients. However, innovations in health care may also pose risks (7). In this regard potential safety gaps are a major concern (8). Innovative technologies might have unknown product malfunctions, undesirable side effects, or a lack of reliability (9, 10). Safety concerns are particularly relevant in the case of high-risk medical devices where their utilization can have serious effects on patients' health. A major challenge is a limited evidence base at the beginning of products' life cycles since there is often only little clinical data available (11). In addition, there is a lack of time and financial resources for costly studies and evaluation reports [e.g., health technology assessment (HTA)] to generate long-term evidence on technologies' effectiveness and safety (12, 13). Therefore, clinical and health decision-makers often lack the evidence base to decide on utilization and coverage of innovative technologies in public health systems.

Usually, physicians decide on the adoption of innovations, which might be influenced by a variety of factors, such as evidence base and individual interests (14). Depending on institutional requirements, decisions on technology adoption might also be made in hospital committees (15). However, negative or delayed coverage and reimbursement decisions may hamper utilization of innovations. This is exacerbated by competition between hospitals, which are under financial pressure and must use their resources efficiently. As a consequence, high costs for innovative technologies can lead physicians to fall back on established standard therapies, which are fully reimbursed by health insurance, as reported, for example, for breast cancer drug therapy in Germany (16). It was already shown that evidence development and reimbursement schemes can influence the utilization of innovative technologies, and therefore the number of patients treated with them in hospitals (17). Vascular surgery is a medical specialty in which many innovative, high-cost medical devices are used. In this discipline, peripheral vascular diseases such as peripheral arterial occlusive disease (PAOD) are treated, e.g., with drug-eluting stents in the upper leg (DES-UL) (14, 17). Diseases of the peripheral vessels are particularly widespread in industrialized countries such as Germany and the USA, and are closely linked to obesity, diabetes, and cardiovascular diseases, leading to increased health resource consumption due to the increased risk of cardiovascular events and limb-threatening ischemia (17–20). These health concerns are omnipresent in the countries. According to the 2023 Health Statistics of the Organization for Economic Co-operation and Development (OECD), over 70% of adults in the USA are classified as overweight or obese. In Germany, although the rate is slightly lower, it remains substantial at ~60% (21). A major problem is also the prevalence of diabetes, which affects a large proportion of the population in both countries, significantly influenced by high obesity rates due to certain dietary and lifestyle habits (22, 23).

In our study, we analyze the adoption and utilization of DES-UL in Germany and the USA. Both countries are leaders in the development of medical technology innovations but have different health care and financing systems and different market approval procedures for medical devices (24). In the European Union (EU) and the US, DES-UL are classified as high-risk medical devices according to the classification rules of the Medical Device Regulation (MDR) (25), previously regulated by the EU Directive 2017/745 (26), and according to the US Food and Drug Administration (FDA) classification processes (27, 28). In Germany and the USA DES-UL products are defined as a “new” technology (used synonymously with “innovative” in the following). In Germany, this is due to their use in a new indication area (UL) compared to the already established DES used in coronary arteries (14, 29). In the USA, the criteria are met that DES-UL products were approved by the FDA in the last 2–3 years and are not substantially similar to existing technologies (30). The aim of this study is to investigate a potential relationship between evidence on efficacy/effectiveness and safety, safety notices & recalls, market approval dates, and country-specific health care financing instruments on the utilization of DES-UL in German and US-American hospitals.

### 1.1 Excursus: financing instruments for medical technologies in the German and the US inpatient care

#### 1.1.1 Germany

In Germany, reimbursement of medical technologies in inpatient care is included in (German) Diagnosis Related Groups (G-DRG). Hospital cases are categorized on the basis of diagnoses and procedures to determine the reimbursement by means of flat rates per cases for both private and statutory health insurance (PHI and SHI), including the utilization of medical devices (14, 31). However, the retrospective calculation of G-DRGs to the extent of average purchasing costs calculated based on the expenses of certain “calculation hospitals” create disincentives for new technologies as those cannot be included in the calculation of G-DRGs in a timely manner (32, 33). Therefore, certain medical technologies that are not yet included in the G-DRG classification may be reimbursed for individual hospitals on the basis of “New Diagnostic and Treatment Methods” (NUB) payments negotiated between individual hospitals and health insurance companies for a period of 12 months. Since 2005, individual hospitals have had the opportunity to submit an annual application to the Institute for the Hospital Remuneration System (InEK) to negotiate additional remuneration with the health insurance companies. Since 2016, according to Section 137h Social Code Book V, hospitals that wish to use NUB based on high-risk medical devices and wish to be remunerated for them, must provide the Federal Joint Committee (G-BA) available scientific evidence regarding the procedure and the device. In the further process, usually the Institute for Quality and Efficiency in Health Care (IQWiG) assesses the technology's benefits, clinical effectiveness, and harmfulness. On this basis, the G-BA decides whether the technology is accepted and can be reimbursed (via NUB payments), is excluded from SHI's benefit basket and reimbursement, or enters a trial phase to gather further evidence (14, 34). Following the “coverage with evidence development” (CED) approach, hospitals can thus receive remuneration for new technologies that goes beyond the regular G-DRG flat rates, while gathering additional evidence (35). This approach aims to ensure efficacy and safety of innovative medical devices before they are widely used in inpatient care. Furthermore, two types of additional payments, so-called “supplementary payments” (fixed and negotiable), may be defined for medical technologies. This is the case if the use of a particular procedure does not yet justify the creation of a separate G-DRG. Fixed supplementary payments may be calculated if the new technology has been used in a sufficient number of patients and deviations from the calculated costs are not too high. The amount is predetermined by the InEK and is the same nationwide. Negotiable supplementary payments are only paid for certain procedures, which are set by the IneK. The amount of the negotiable supplementary payments is determined at local level in negotiations between individual hospitals and the health insurance companies (34, 35).

#### 1.1.2 United States

In contrast to Germany, there is no standardized reimbursement system for financing of medical devices across all health insurance companies in the USA. The Medicare health care program considers severity and complexity of patients' illnesses or medical conditions (Medicare Severity Diagnosis Related Groups: MS-DRG) when defining flat rates based on patients' diagnoses. Medical devices are reimbursed as part of it (36). Utilization of technologies may also be reimbursed via other measures (e.g., agreements with hospitals, value-based contracts), or reimbursement may be specific to health insurance companies. DRG billing is, besides Medicare, commonly used by some private insurers. The approach to financing medical technologies therefore depends on the health insurance company. A significant portion of the population is covered by private health insurance, either through employer-sponsored plans (60–65% of the US population in 2018), or through individual purchases (37). These private insurance companies play an important role as they negotiate rates with medical facilities, and subsequently determine which technologies are reimbursed. In addition, the public health care programs, particularly Medicare and Medicaid, also play a central role in this landscape. Medicare primarily serves older and disabled people, while Medicaid is primarily intended for the low-income population (38). It is also possible that patients without insurance, or patients undergoing treatment that is not covered by their insurance, must pay out of pocket. These direct payments are another important component of the US medical technology financing structure (39). In the USA, there is a concept similar to NUB payments, the so-called “New Technology Add-on Payments” (NTAP). NTAPs are a mechanism used by the Centers for Medicare & Medicaid Services (CMS) to reimburse the costs of implementing new medical technologies and procedures that provide significant improvements in patient treatment or diagnosis, in addition to standard MS-DRG payments. These additional payments are intended to compensate hospitals for higher costs associated with utilization of innovative technologies and treatment methods before they are fully integrated into the MS-DRG payment system (40). Also, the Medicare program may in some cases provide conditional coverage for innovative medical devices (Medicare CED) if manufacturers agree to collect efficacy and safety data as part of CED studies (41).

## 2 Materials and methods

### 2.1 Systematic evidence search and case numbers identification

We conducted systematic searches for evidence, including **scientific articles, clinical trial registry entries, HTA reports**, and **clinical guidelines**. In addition, we searched for **safety notices & recalls, market approval dates** in the EU and the USA, and relevant **financing instruments**. Furthermore, we identified **hospital case numbers** for both countries, depicting utilization of DES-UL in inpatient care. We used various sources and approaches for search and analysis, which are presented in more detail below.

To identify **scientific articles** on studies, we conducted a systematic literature search in the biomedical databases PubMed, Medline (via OVID), Embase (via OVID), and the Cochrane Library for the overall observation period 2006 to 2022 (initial: 2006–2017, and update search: 2017–2022). To keep the data for this study up to date, an update search was carried out. We used the PICO scheme (= population, intervention, comparison, outcomes) for developing our search strategy and concept (42). These items also represented the inclusion and exclusion criteria for the subsequent literature selection. The application of the PICO scheme to our research question is shown in Table 1.

**Table 1:** PICO scheme and literature selection criteria.

**PICO items**		Inclusion criteria^*^	**Exclusion criteria**
Population and Indication		•Adult population (≥18 years) •Peripheral occlusive disease •Peripheral arteries and veins of upper leg, femoral vessels (below the hip joint, femoral, saphenous)	•Children, animals, *in-vitro*-studies •Saphenous vein graft approach •Vessels of lower leg (e.g., popliteal, infrapopliteal), and other (e.g., coronary, intracranial and brain, abdominal, iliac, brachial)
Intervention		•Implantation of one (or more) DES^**^	•Exclusive implantation of non-DES (e.g., BMS), bioresorbable stents, and stent prostheses (e.g., stent-grafts) •Exclusive utilization of other therapy methods (e.g., balloon catheters)
Comparator		No restrictions	
Outcomes		No restrictions	
Study design		•Primary and secondary studies, incl. interventional and observational studies, case studies, case series, registry data analyses, reviews, meta-analyses, guidelines, HTA reports, etc.	•Exclusive description of treatment algorithms or sequences, processes, and technical handling of technology •Protocols, conference abstracts, editorials, letters to the editor, commentaries, errata, notes
Time frame	Initial search	2006–2017	Before 2006 and after 2017
	Update search	2017–2022	Before 2017

^*^Due to the initial aim of our research project, publications on DES with utilization in the lower leg and below the knee were also searched; ^**^German procedure code OPS 8-841 (see Appendix A.3); BMS, bare metal stent(s); DES, drug-eluting stent(s); HTA, health technology assessment.

To define search terms, we used technology assessment documents by the German Medical Service (MDK) of the National Association of Statutory Health Insurance Funds [Spitzenverband Bund der Krankenkassen (GKV-Spitzenverband)], the German procedure code classification system (OPS), and the International Statistical Classification of Diseases and Related Health Problems (ICD). Details are published elsewhere (11). The concept, strings, dates, and hits of the (initial and update) searches in the biomedical databases can be found in Appendix A.1.

Furthermore, we conducted additional searches in **clinical trial registries** [World Health Organization (WHO), and clinicaltrials.gov database], **HTA databases**, and **clinical guideline databases**. For website screening, we used search terms including DES-UL product names, diseases that might require utilization of DES-UL, and technology synonyms (e.g., “drug-eluting stent”). HTA reports were only included in analysis in case they explicitly investigated clinical benefit of DES-UL. Used literature sources, including links to websites of clinical trial registries, HTA databases, and clinical guideline databases, and search/screening terms are listed in Appendix A.2.

We searched for **safety notices & recalls** on the website^1^ of the German Federal Institute for Drugs and Medical Devices (BfArM), which documents safety events in Germany. We also conducted a search for international documentation on the “implant-files” website,^2^ an initiative of the International Consortium of Investigative Journalists. The search for **dates of market approval of DES-UL products** was realized by searching the internet via Google for each product individually. As search terms we used the product names, which were extracted from the full texts after selection of relevant study articles. Depending on the country, the search terms “CE” [Conformité Européenne – CE marking in the EU (43)] for Germany, and “FDA” [as the FDA is the approval authority (44)] for the USA were added to the search strings. In addition, information regarding **financing instruments** was researched. For this purpose, the website^3^ of the InEK was searched for German data, and data on G-DRG and NUB payments were extracted for DES-UL. We limited our analysis on financing instruments in the USA to Medicare, as this documents the billing of flat rates per case in hospitals and the financing mechanisms of innovative technologies (45). In this regard we searched the CMS website^4^ for information on MS-DRG and its Medicare Coverage Database (MCD) for information on additional payments.

The utilization of DES-UL was operationalized by annual **case numbers** of patients treated in hospitals in Germany and the USA with one or more implanted DES-UL during one hospital stay between 2008 (Germany)/2010 (USA) and 2020. DES-UL procedure codes were required to determine the case numbers. These were identified via the website^5^ of the German Institute for Medical Documentation and Information (DIMDI) and a US code website.^6^ The case numbers from Germany were obtained from the German Research Data Center (FDZ) on Health of the German Federal Statistical Office [Statistisches Bundesamt (Destatis)] by query: data basis is the G-DRG statistics representing billing data from German hospitals per year, recorded as part of the G-DRG system, in which hospital cases are classified based on diagnoses and procedures (46). The data from the USA were obtained from the National (Nationwide) Inpatient Sample (NIS) datasets of the Healthcare Cost and Utilization Project (HCUP) of the US Agency for Healthcare Research and Quality (AHRQ) (47, 48) and analyzed using STATA software (version 15). HCUP's NIS include annual data on hospital inpatient care in the USA that encompasses all payers (i.e., Medicare, Medicaid, private insurance, uninsured) and provides a national estimate of the number of inpatients. The development of case numbers is illustrated graphically. Additionally, the case numbers were standardized to 100,000 inhabitants/population (49) (basis for both countries: total national population per year; all hospital cases, regardless of payer). The procedure codes used in the analyses can be found in Appendix A.3. Population numbers per year, data sources, and the formular for standardization calculation are given in Appendix A.4.

### 2.2 Selection of evidence and assessment of risk of bias potential

**Figure 1:**
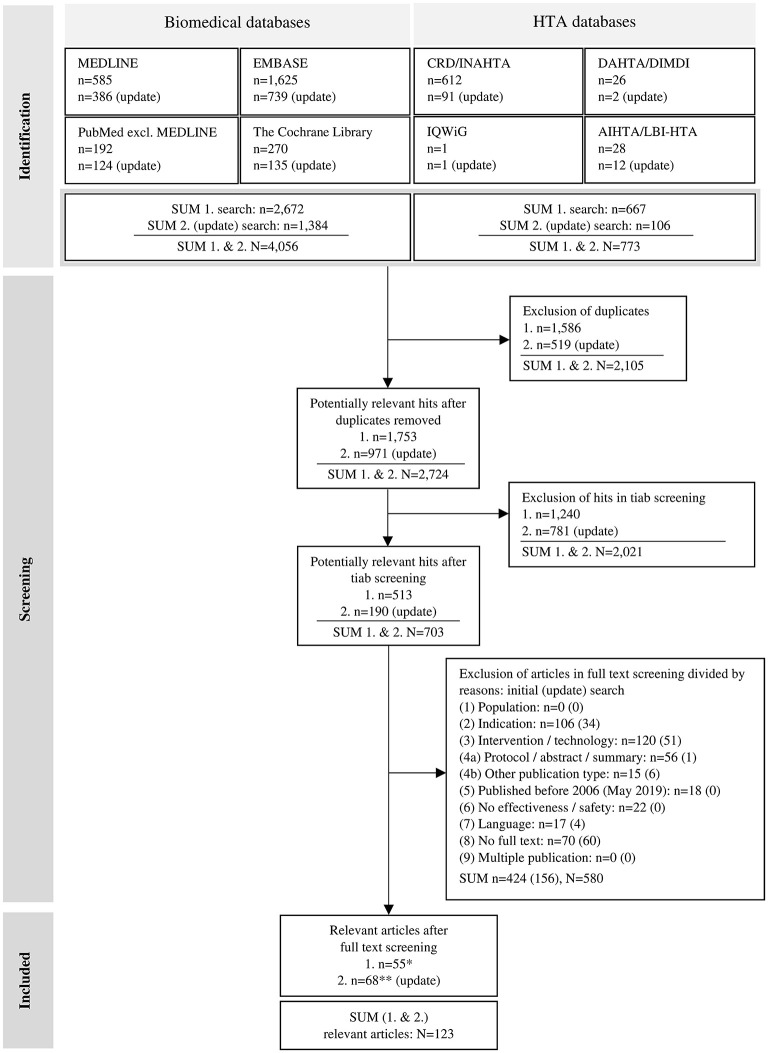
Flow chart of the literature selection process, adapted from the PRISMA 2020 flow diagram by Page et al. (51); licensed under CC BY 4.0. AIHTA/LBI-HTA – HTA Austria: Austrian Institute for Health Technology Assessment GmbH (AIHTA)/former Ludwig Boltzmann Institute for Health Technology Assessment (LBI-HTA); CRD/INAHTA – University of York: Centre for Reviews and Dissemination (CRD) & International Network of Agencies for Health Technology Assessment (INAHTA); DAHTA/DIMDI – German Agency for Health Technology Assessment [Deutsche Agentur für Health Technology Assessment (DAHTA)] & German Institute for Medical Documentation and Information [Deutsches Institut für Medizinische Dokumentation und Information (DIMDI)]; HTA – health technology assessment; n – number of articles/publications; N – sum of articles/publications; tiab – title/abstract; IQWiG – Institute for Quality and Efficiency in Health Care [Institut für Qualität und Wirtschaftlichkeit im Gesundheitswesen (IQWiG)]; */** not included: n = 148/n = 10 narrative reviews, n = 6/n = 17 case studies [level of evidence (LoE) V].

The screening and selection of the literature identified in the biomedical databases was conducted using EndNote software (version X9). After uploading the literature search hits into EndNote, duplicates were removed, initially via an automated process, and additionally via manual comparison by one reviewer (SF). The subsequent literature selection was realized in a three-step process using a category system created in EndNote according to the various selection criteria: (I) title/abstract (tiab) random sample screening, (II) tiab screening of further hits [remaining after (I)], and (III) full text screening. The inclusion and exclusion criteria for literature selection (Table 1) were predefined, based on the items in the PICO scheme: (1) population, (2) indication, (3) intervention, (4) study design, and (5) time frame (see Section 2.1); and other criteria: (6) no effectiveness or safety investigated, (7) language (whether English nor German), (8) no full text available, and (9) multiple publication without relevant added value. In screening step (I), we used the rapid review approach suggested by the Cochrane Collaboration (50): a random sample of 10% of the hits was drawn from the literature pool of potentially relevant publications using RStudio software. After independent screening of the sample by two reviewers (SF, HE), results were compared. In case of disagreement, inclusion and exclusion criteria were discussed and adjusted. If necessary, a third person was involved until consensus was reached. Further screening (step II and III) was carried out by one reviewer (SF). The full texts of potentially relevant hits were searched through the automated literature search in EndNote and were retrieved manually, e.g., by searching library databases and Google, and by requesting documents directly from authors. The literature selection process was conducted according to the PRISMA criteria and is illustrated in a flow chart (Figure 1) (51).

The registry entries identified in the WHO database were first screened by title using the selection criteria based on our PICO scheme. Potentially relevant hits were then exported to an Excel file. Secondly, the full text registry entries were downloaded, screened, and the relevant ones selected. The search hits from the clinicaltrials.gov database, representing entries with detailed information on health conditions and technologies information, etc., were also imported into an Excel file and selected in a multi-stage screening process according to our PICO criteria: (1) indication, (2) intervention (if available: product name), and (3) plausibility in (3.1) title and (3.2) registry entry. Subsequently, relevant entries from both databases were then merged in Excel, duplicates were removed, and data were extracted to predefined variables [e.g., register ID, study title, study status, (estimated) date of study completion].

Clinical guidelines were considered for further analysis if they addressed the use of DES-UL and were published after 2005. No strict restrictions were defined regarding comparative interventions. Relevant guidelines were not included in the literature analysis but were analyzed separately. Extracted information per guideline contain reference and background [author (year)/institution, title, country focus], targeted technology [DES technology (product name), indication], recommendation [level of evidence (LoE), number of articles cited in clinical guideline (reference(s))/grade of recommendation (GoR), statement], and source (website URL) of guideline.

Identified randomized controlled trials (RCTs) were assessed regarding their potential risk of bias (RoB) according to the requirements of the G-BA's Rules of Procedure (p. 164 ff) (52). This standardized table was used to assess the following reporting criteria: 1. adequate generation of the randomization sequence, 2. concealment of the group allocation, 3. blinding [of patients and (further) treating persons], 4. outcome-independent reporting of all relevant endpoints, and 5. absence of other aspects. The fulfillment of the criteria was rated as “yes”, “no”, or “unclear” resulting in a low or high potential RoB at study level. If the assessment criteria (1.) or (2.) could not be answered with either “yes” or “unclear”, trial LoE was downgraded from Ib to IIb.

### 2.3 Data extraction and analysis

For data extraction and analysis, a distinction was made between primary studies of LoE I-IV (e.g., RCTs) and secondary studies of LoE Ia & IIa (i.e., systematic reviews, meta-analyses, HTA reports). We determined the articles' LoE in accordance with the G-BA's Rules of Procedure, 2nd Chap., §11(2 and 3) (52). Difficulties in classification regarding LoE were discussed and agreed in the reviewer team. Comparative studies that examined DES-UL in both the intervention and control group were downgraded to non-comparative studies. Publications of LoE V (e.g., case reports) and other study designs were excluded. Data from the included articles were extracted in an extraction sheet in Excel using predefined variables, e.g., number of patients (secondary studies also: number and total population sizes of studies included in analyses of reviews and reports), median follow-up, reported endpoints, and the authors' conclusions from abstract and main text. Based on the authors' conclusions extracted from each publication, a single reviewer (SF) assigned an “author assessment” to reflect its core statement. The assessment categories – “positive”, “indecisive”, “neutral”, or “negative” – and their definitions are shown in Table 2. As an additional study design characteristic, length of follow-up was considered to determine long-term studies. Accordingly, primary study articles were selected if they reported a (median) follow-up of ≥5 years or explicitly described the study as “longitudinal”.

**Table 2:** Author assessment: categories and definitions.

**Assessment category**	**Definition: The article's author/authors …**
Positive	… conclusions are consistently positive (regarding efficacy AND safety, and across patient groups). If neutral (e.g., “equally safe”) and positive (e.g., “effective”) statements are combined, the results are considered positive.
Indecisive	… conclude that no definite statement can be made, e.g., because •there is better efficacy but worse safety, •results are not generalizable to all patients (recommended for some patient groups but not for all), •the author/authors do not make a statement in the conclusions, neither explicitly (“no statement is possible”) nor implicitly (“could”, “maybe”, “possibly”), or •only surrogate parameters are reported and consequently no inference of benefits is possible.
Neutral	… conclude no difference between intervention and comparison intervention.
Negative	… conclusions are consistently negative (regarding efficacy AND safety, and across patient groups). If neutral (e.g., “less safe”) and negative (e.g., “less effective”) statements are combined, the results are considered negative.

**Figure 2:**
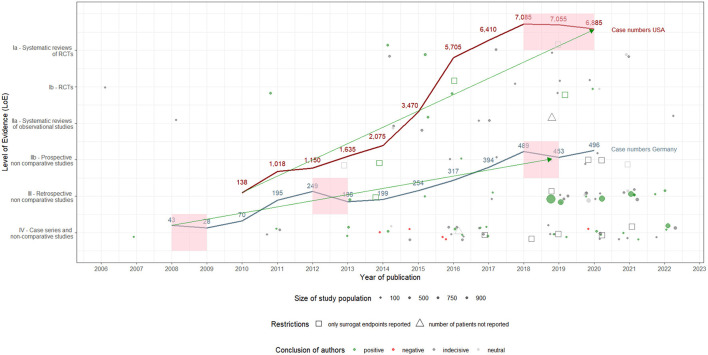
Utilization of drug-eluting stents in the upper leg in Germany and the USA: development of hospital case numbers (2008–2020) and evidence (2006–2022), including author assessments.

Color of row, color of article figure in Figure 2. Source: adapted from (11).

## 3 Results

### 3.1 Literature search in databases

A total of 4,829 hits (initial: *n* = 3,339; update: *n* = 1,490) were identified. After removing duplicates, 2,724 potentially relevant publications were included in the subsequent screening steps. The pool from the initial search in biomedical databases (2006–2017) comprised 1,753 hits resulting in 176 hits included in the random sample screening. After the full text screening, 123 articles remained and were included for further consideration. An overview of the hits in the different databases and literature searches (initial and update), and their selection process is shown in the flow chart in Figure 1.

### 3.2 Evidence and study results (author assessment) development

We identified 117 **publications on primary and secondary studies** of various study types that clearly or exclusively analyzed DES-UL. Over time, the number of these publications has steadily increased from 2013 through 2022. A particularly large number of articles were published in 2019 (*n* = 20) and 2020 (*n* = 21). Both the number of non-comparative studies (LoE IV) and studies with high LoE (< IV) increased, particularly from 2014 to 2016. The Zilver PTX stent, the first certified DES for femoral vessels, was investigated in many studies of the identified articles (*n* = 65 articles on primary studies). Most RCTs (7 out of 9) focused on the Zilver PTX stent. During the period from 2013 to 2022, there was also an increase in systematic reviews and meta-analyses, particularly since 2014. In six articles, differentiation between results for UL and lower leg (LL) vessels in the studies was insufficient. Therefore, these articles were excluded from data extraction and their results are only presented narratively. Of the identified publications, 41 articles received a “positive” assessment from the authors, and 58 articles were deemed “indecisive”. Of the 10 RCTs, conclusions of five were assessed as “positive”, four as “indecisive”, and one as “neutral”. The articles on studies with LoE IV were predominantly assessed as “indecisive” (*n* = 24 of 49), followed by “positive” (*n* = 18); five articles were assessed as “negative” and two as “neutral”. From 2013 through 2022, there was an increase in the number of publications on studies with large patient populations, as well as on studies with high LoE (Ia, Ib, and IIa), which began increasing in 2014. Among articles published from 2019 to 2022, “unclear” (*n* = 34 of 57) and “positive” (*n* = 19) author assessments dominated, and only one article received a “negative” assessment. Conclusions of the studies investigating both UL and LL areas were categorized either as “positive” or “indecisive” (*n* = 3 articles each). Of the analyzed studies, *n* = 8 were considered as long-term studies (53–60), mostly published toward the end of the observation period (2019–2021). Exceptions were Wooster et al. (59) (LoE IV: “indecisive”) and Dake et al. (56) (LoE Ib: “positive”), both published in 2016. Among the various endpoints assessed, QoL is the only one reflecting patient perspectives. It was reported in *n* = 4 studies (61–64), but all were conducted at the end of the observation period (2019–2021). A detailed description of the evidence and the study results (author assessment) development of the articles, including references, is given in Appendix A.5. Appendix A.6 contains an overview of the identified articles and extracted data for LoE Ib, IIb, III, and IV studies, and Appendix A.7 includes an overview for the LoE Ia & IIa studies. Appendix A.8 contains an overview of the publications identified in the HTA database searches. Results and a short description of the RCTs' assessment regarding potential RoB can be found in Appendix A.9.

In the **clinical trial registries**, 37 entries on studies investigating DES-UL were identified. First registry entries for DES-UL trials were made in 2005, investigating the Zilver PTX (65) and the S.M.A.R.T. stent (66). Most entries (*n* = 18) refer to the Zilver PTX stent. Other entries report on investigations of further DES-UL, e.g., the Eluvia and the Dynalink stent, or do not state a product name. We also found entries for the trials ILLUMINA (trial status: completed) (67), and G-streamPAD (trial status: recruiting) (68) investigating the two new drug-eluting self-expanding stents (SES) “NiTiDES” and “G-stream” for utilization in femoral vessels (10/2023). Another entry is the ELITE trial (69), investigating drug-coated stents in femoropopliteal artery lesions, and still recruiting participants (10/2023). An overview of the identified entries in clinical trial registries and relevant registry content can be found in Appendix A.10.

We identified three **clinical guidelines**: one focusing on the indication PAOD, published by the German Society for Angiology and the Society for Vascular Medicine in 2015 (70), one addressing peripheral arterial disease (PAD) by the European Society for Vascular Medicine (ESVM) (71), and one addressing chronic limb-threatening ischemia by the European Society for Vascular Surgery (ESVS) (72), both published in 2019. The guidelines do not provide a clear recommendation regarding utilization of DES-UL. Additionally, the supporting data basis used is small, referencing only *n* = 2 (*n* = 4 articles in total) (70), *n* = 2 (71), and *n* = 1 RCT articles (72). For example, the ESVM guideline reports a reduced risk for restenosis and target lesion revascularization over 5 years with DES-UL compared to provisional bare metal stents (BMS), based on an RCT by Dake et al. (73). In contrast, the ESVS guideline does not consider DES-UL as a distinct technology but rather groups it under “drug-eluting technologies” (72), citing only Dake et al. (73). An overview of the clinical guidelines and relevant guideline content can be found in Appendix A.11.

Many **safety notices & recalls** were identified for different products mentioned in this study. A total of 11 safety notices & recalls were published for the product Zilver PTX. Of these, 10 entries were published in 2023 in different countries (e.g., Poland, Germany, USA, Hong Kong, New Zealand, Australia) (74–80). Some entries refer to this incident as a “(serious) adverse event” (74–77). One of the recalls reports a death (76). We were unable to identify the full texts for a further three entries. A total of six safety notices & recalls were identified for the product Eluvia (five in 2017). In four of these entries, the content is similar, but date and country of publication differ (between 11/06 and 11/17/2017, in Germany, Australia, Poland, and the Netherlands) (81–84). We could not identify the full text for one entry. A safety notice published in 2020 in Germany refers to Paclitaxel-eluting stents for treatment of PAOD including the Zilver PTX and the Eluvia stent (85). This notice reports an increased risk of late mortality after DES-UL treatment, citing the results by Katsanos et al. (86). Safety notices & recalls were also published for the stents of other indication areas (S.M.A.R.T., Cypher, Xience V) (87–89). An overview of the results of our search for safety notices & recalls, including relevant content and their sources, along with a list of search sources and terms used (including product names), can be found in Appendix A.12.

We identified a total of three **DES products approved** for utilization in the UL: the Zilver PTX and the Eluvia stent were approved in the EU (CE certification) three (2009 vs. 2012) and two years (2016 vs. 2018) earlier than in the USA (FDA approval) (90–93). We did not find any approval data for the Dynalink-E stent. Two new drug-eluting SES-UL are the NiTiDES [CE certified in 2021 (94)] and the G-stream stent (68) (no information on approval). The other stents used in the studies are stents that were (originally) approved for other indication areas (primarily coronary stents): S.M.A.R.T. (95), Cypher (96, 97), Taxus Liberté (98, 99), and Xience V (100, 101). An overview of information regarding the DES-UL products' market approval in the EU and the USA, including manufacturer names and countries, and a graphical representation of the products' market approval dates over time alongside the case number curves is given in Appendices A.13, A.14. A detailed description of the development of the products' market approval over time can be found in Appendix A.15.

We found the following information on **financing instruments**: in Germany, utilization of DES-UL was reimbursed from 2009 to 2011 via NUB payments (102–104). No information was found for previous years. Since 2012, DES-UL have been compensated via the G-DRG system (105). The coding of DES-UL in the USA via MS-DRG is documented on the CMS website as of 2017 (106). DES for the utilization in the lower extremity arteries, including femoral arteries, were added to the CMS coding system in 10/2015 and utilization has been reimbursed since then (107). We were unable to identify any information on financing instruments for DES-UL in the US inpatient sector for years prior to this, searching the CMS website (see Section 2.1). An overview of the financing instruments identified for both countries, and details on the search process can be found in Appendix A.16.

Starting from 43 **hospital cases** in 2008, the utilization of DES-UL in Germany decreased by 35% to 28 cases in 2009. This decline was followed by a significant 150% increase to 70 cases in 2010 and an even sharper rise of 179% to 195 cases in 2011, continuing to grow to 249 in 2012. However, in 2013, there was a decrease of 25% to 186 cases. The trend then generally shifted upwards through 2018, peaking at 489 cases, with fluctuations in subsequent years (2019: a decrease to 453 cases; 2020: an increase to 496 cases). In the USA, utilization of DES-UL was first coded in 2010 with 138 cases, followed by an increase of 638% to 1,018 cases in 2011. The upward trend continued (2012: 1,150 cases; 2013: 1,635 cases) to 3,470 cases in 2015. In 2016, an annual growth rate of 64% was observed, with the number of cases rising to 5,705. The subsequent years saw more stabilized growth, with the peak of 7,085 cases occurring in 2018, followed by slight decreases in 2019 (7,055 cases) and 2020 (6,885 cases). The overall trend analysis shows an increasing utilization of DES-UL in both countries, with the USA recording a stronger increase, particularly between 2010 and 2016. In contrast, Germany experienced an uninterrupted and constant growth of case numbers starting 2014 to 2018. When adjusting both countries' cases to 100,000 inhabitants, the same picture emerges: case numbers for Germany and the USA increase overall over time. However, the case number curve for the USA rises more sharply since 2014 compared to Germany. Specifically, in 2016, the standardized case numbers in the USA were up to 4.7 times higher than in Germany [0.38 (Germany) vs. 1.77 (USA)]. The development of the case numbers for both countries is depicted in Figure 2. An overview of the standardized case numbers is given in Appendix A.4. A figure presenting the development curves for the standardized case numbers for Germany and the USA can be found in Appendix A.17.

### 3.3 Short synthesis: assumption of influence of evidence and further events on case numbers' development

**Figure 3:**
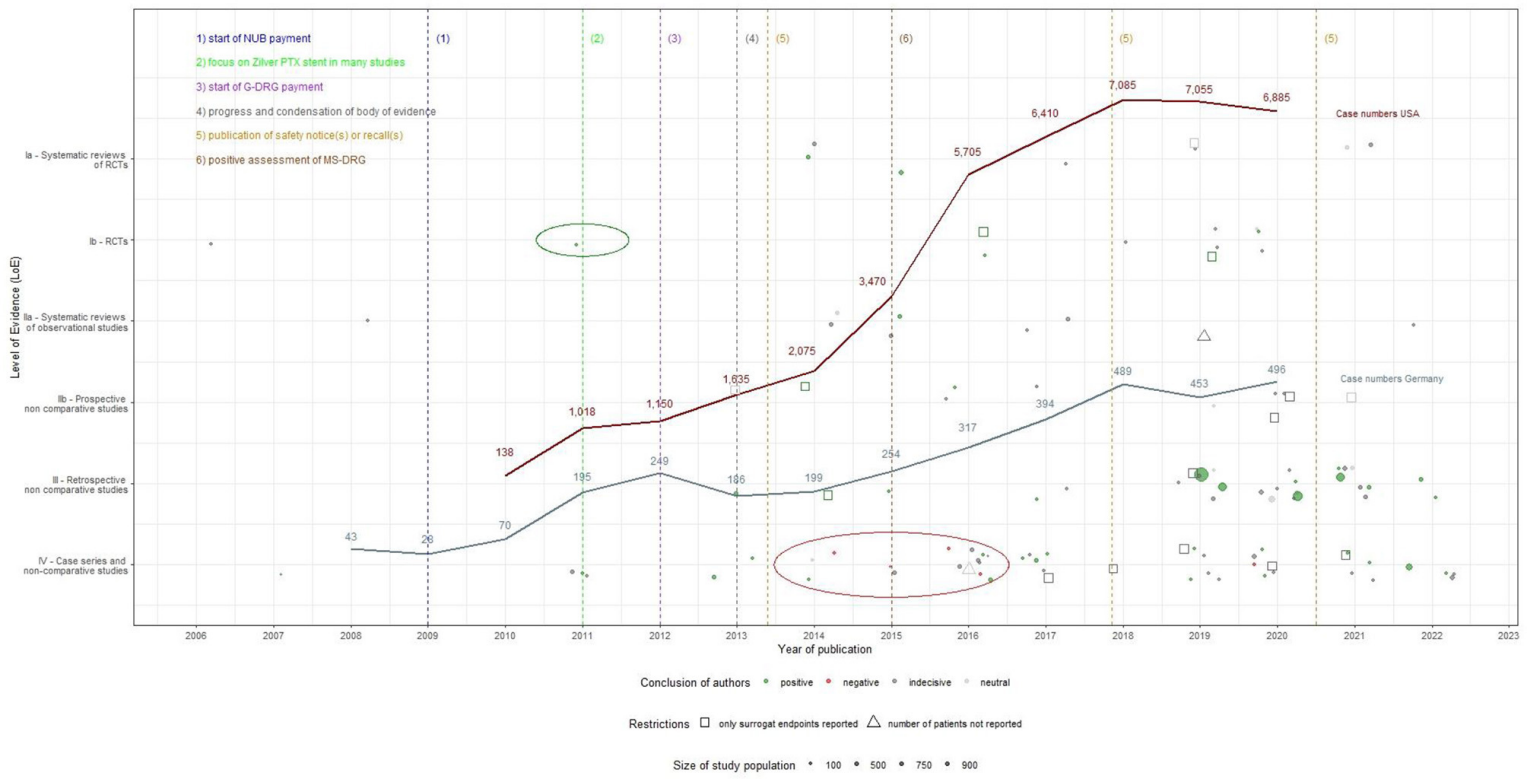
Utilization of drug-eluting stents in the upper leg in Germany and the USA: events over time (2006–2022).

The utilization of DES-UL started in Germany in 2008 and was initially met with restraint. However, case numbers increased from 2009, likely driven by the introduction of NUB payments and the CE certification of the Zilver PTX stent. In the USA, the utilization of DES-UL began in 2010 and increased slowly but steadily. The decrease in case numbers in Germany in 2013 could be attributed to published safety notices & recalls. Yet, from 2014 onwards, the number of cases increased again, possibly due to accumulating evidence, including articles with positive study results and one RCT with a long-term follow-up (56), as well as the availability of the Zilver PTX stent. In the USA, the increase in case numbers accelerated following a positive evaluation of DES-UL by CMS in 2015. Nevertheless, case numbers in both countries began to decrease from 2018, potentially due to the publication of articles with negative study results and safety concerns, particularly regarding the Eluvia stent. In 2020, the number of cases in Germany increased again, which may be explained by the increased publication of articles with positive study results and recommendations in international clinical guidelines. This increase, however, appeared to have had no effect in the USA. The products' approval may also have influenced the development of the case numbers, not only in their own country but also in the other country. In Germany, for example, case numbers increased again following both EU certification and FDA approval of the Eluvia stent. A detailed description of the synthesis, which makes assumptions about the influence of evidence and further events on case numbers' development, can be found in Appendix A.18. Figure 3 shows the development of the evidence and the different reported events alongside the case number curves for both countries.

## 4 Discussion

This is a comprehensive study considering case numbers of DES-UL utilization by hospitals in Germany and the USA over time, alongside various events that may have influenced the technology's adoption decision and utilization by physicians in routine care. These events include publications of evidence, which are scientific articles, clinical guidelines, safety notices & recalls, products' market approval dates, and the availability of financing instruments. Eckhardt et al. (11) have already shown that these events may affect utilization behavior regarding new technologies in hospitals and thus can determine development of case numbers. Overall, we observed a trend of an increasing utilization of DES-UL in both countries analyzed. Available evidence and published safety notices & recalls may be one reason for these developments but may also have triggered interruptions in trends. Country-specific variations in the case number curves are probably due to country-specific events, such as safety notices & recalls – particularly in 2013 –, changes in financing instruments (e.g., the shift from NUB to G-DRG funding in Germany in 2012), and market approval dates (e.g., FDA approval of the Eluvia stent in the USA in 2012). These factors may have played an important role in decision-making for or against the (initial) utilization of DES-UL.

As part of evidence synthesis, the extent to which the various events could have had an influence on the development of the case numbers in both countries is to be assumed. The body of evidence developed over time, i.e., at the beginning of the observation period there are a few publications on studies, mostly of low LoE and with small population sizes. Over the years, studies of higher LoE and with larger study population sizes were conducted and articles published. Particularly from 2013 onwards, the body of evidence further increased and has condensed. This development of evidence in the overall observation is also known for other new technologies (11). Nevertheless, especially in the early years of products' life cycles, decision-makers in hospitals and health care policy often lack evidence on efficacy/effectiveness and safety, which should be the basis for making decisions on the utilization, coverage, and reimbursement of innovative technologies (108, 109). Physicians may hesitate to use new technologies in everyday care immediately after they enter the market (110). On the one hand, this caution can prevent unknown harm to patients from new technologies (7). On the other hand, patients might miss out on possible health benefits of innovative treatments compared to standard treatment. Since 2016, this dilemma has been addressed in Germany on the basis of procedures for high-risk medical devices in inpatient care. Since then, evidence for funding decisions of NUB payments is required. Nevertheless, if neither the benefit nor the harmfulness or ineffectiveness of the technology can be considered sufficiently proven, the G-BA decides on a trial to gather further evidence using a CED approach (14). Still, only a few CED studies have been initiated or are in the process of being set up.

Regarding DES-UL, the Zilver PTX stent has been increasingly investigated in studies since 2011, providing research results for (clinical) decision-makers. Consequently, physicians were no longer forced to use technologies initially approved for another indication (e.g., coronary DES) in off-label use. According to our analysis, the Zilver PTX stent was often investigated in primary studies (*n* = 66), providing results for decisions. In contrast, other stents for utilization in the UL have been less frequently investigated in primary studies, e.g., the S.M.A.R.T. (*n* = 1) and the Dynalink-E stent (*n* = 1). The development of the body of evidence reveals the dilemma of newly introduced technologies: publications on primary studies of high LoE, i.e., RCTs, or cohort studies of large study population sizes are only conducted over the years and their results are published in later phases of products' life cycles (17). Especially the results from these high-quality studies are necessary for health care decisions from day one of their market entry. However, time and costs invested in these studies are very high. The Medical Research Act 2024 could provide a solution, aiming to improve the conditions for development, approval, and production of medical devices (and pharmaceuticals) in Germany. The law is intended to reduce bureaucratic barriers, accelerate innovation processes (e.g., through accelerated approval procedures), and also to ensure high standards of patient safety and care (111, 112).

During the whole observation period, there was insufficient consideration of DES-UL in publications with a “high weight” for routine care, e.g., studies of LoE Ia & IIa and clinical guidelines on PAD. In the latter, DES technologies were often summarized (e.g., DCB, DES), and the cited studies rather focused on standard treatments (e.g., BMS, balloon angioplasty) (71, 72). Consequently, it was not possible to draw any clear conclusions about DES-UL from these sources. However, clinical guidelines are systematically developed statements that reflect the current state of knowledge regarding the appropriate care for specific health issues, typically based on studies with a high LoE (usually RCTs) (113, 114), accordingly representing an important decision-making instrument for physicians in their daily clinical practice. Nonetheless, DES-UL were first addressed in 2015 in a clinical guideline on the treatment of PAD, but the guideline did not explicitly recommend DES-UL for this indication (70). Two further clinical guidelines followed in 2019, one reporting positive study results, based on a single RCT (71), another one recommending drug-eluting devices in the UL, but without explicitly addressing DES (72). It can be assumed that physicians lacked a simple and clear decision-making basis about DES-UL. This may have had an influence on DES-UL utilization in hospitals. Clinical guidelines should have focused on DES-UL, and been updated over time, especially as the body of evidence has evolved over the years to allow for this. According to registry entries, DES-UL are still being investigated in clinical studies, even if there was a reorientation to SES (e.g., NiTiDES). There is still research and development activity by the medical technology industry, especially in the USA. Almost all manufacturers of DES-UL are based in the USA [one exception: the new Alvimedica stent (Turkey) (115)]. Accordingly, there is a demand for DES-UL from hospitals, which is probably also due to an increasing disease burden related to peripheral vascular disease caused by obesity and diabetes. And obviously, the clinical need for DES in the indication area of the UL has existed for a long time, as can be seen in stents' off-label use and their development as drug-eluting technology (i.e., drug-coating of the S.M.A.R.T. stent, which is originally a BMS). It is important that needs in clinical practice are identified and communicated promptly to prevent reliance on off-label use and potentially unsafe treatment alternatives. The evidence base and overall positive (and “indecisive”) study results may be one reason for DES-UL utilization. Nonetheless, there is a need to conduct further studies in the future, particularly to investigate long-term results of the utilization of DES-UL and new products that may enhance the robustness of study findings. One example of such work included in our sample was the study by Dake et al. (56). Several safety notices & recalls (*n* = 17) for the Zilver PTX and the Eluvia stent reported (severe) adverse events during the observation period. In addition, the meta-analysis of Katsanos et al. (86) showed an increased long-term mortality. Both DES are approved for Europe/Germany and the USA and accordingly meet (safety) criteria of both systems, yet there have been these concerns. This underlines the need for long-term observations and appropriate post-market surveillance systems to timely identify risks. Fargen et al. (116) address this need by calling for revising the market approval processes to guarantee patient safety. With the introduction of the MDR, there have been changes to the procedures in Europe, but these only relate to the monitoring of medical devices' regulation processes (117). The results of our study confirm the importance of evidence generation through clinical trials and other studies during the entire utilization period of (innovative) technologies, and a timely and free accessible publication of study results that can be used by physicians, (clinical and political) decision-makers, and manufacturers.

Regarding financing instruments, in Germany, DES-UL were funded via NUB payments between 2009 and 2011. From 2012 onwards, the technology was financed as part of G-DRG flat rates. Funding in the inpatient sector in Germany aims to facilitate access to innovations (118, 119), and at the same time to ensure economic efficiency and quality in the health care system (120). Therefore, the integration of new technologies is supported by the German reimbursement system (which includes the various approaches, e.g., NUB and additional payments) that promotes continuous updating and adaptation to medical progress (34, 121). In 2012, the first year of G-DRG payments, the increase in case numbers continued as in previous years. The G-DRG system enables all hospitals to receive reimbursement, while NUB payments are only paid to those hospitals that have applied for them via a predefined process at the InEK and have negotiated an additional payment with the health insurance companies. It shows that the remuneration of technologies is probably a decision criterion for physicians, as already analyzed in one of our previous studies (14). Nevertheless, in 2013, the case numbers decreased, but this decrease could be attributed to several international safety notices & recalls (*n* = 10). According to Rice et al. (38), the USA is a global leader in the development and use of innovation payment systems to improve the value of services provided. However, the funding paradigm for medical technologies in the USA is complicated, with decisions often based on expected clinical benefit, costs analysis, and projected profitability (122, 123). To ensure that these technologies fulfill on their promises, there are formal evaluation processes that aim to rigorously assess technologies' effectiveness and overarching benefits prior to widespread adoption (116). However, we did not find information on the reimbursement of DES-UL for the entire observation period for the USA. Furthermore, regional disparities within the countries may have influenced the utilization of DES-UL. Hospitals in rural areas may face certain barriers to implementing advanced vascular technologies such as DES, including financial constraints, differences in health care structures, and variations in referral networks. Additionally, the availability of these technologies may differ between urban and rural centers (124–128). These challenges could contribute to differences in the adoption of new technologies, as urban centers often benefit from higher procedural volumes, greater cost efficiency, and better access to advanced medical infrastructure. Regional variations in the utilization of vascular interventions, as observed between federal states in Germany, suggest that economically weaker regions may perform fewer such procedures (129). However, differences in utilization do not necessarily indicate underuse in some regions but may also reflect variations in clinical decision-making or resource allocation. Similarly, in the USA, state-level disparities in health care policies and economic resources appear to play a role in the adoption of vascular interventions, potentially contributing to differences in the utilization of DES and other advanced technologies across regions (125, 130). Future studies should systematically investigate how these disparities affect long-term patient outcomes and access to care, ensuring that policy interventions support equitable and appropriate access.

However, the analysis of DES-UL utilization reflects challenges to be observed in the implementation of new medical technologies. Regulatory complexity can be seen as a barrier, with reimbursement decisions and liability concerns often delaying integration into clinical practice (14). For example, time-consuming economic evaluations to demonstrate cost-effectiveness before widespread adoption of technologies can slow the process (131). Beyond regulatory and economic factors, organizational structures of medical institutions may also influence technology adoption. Studies have shown that hospitals with strong leadership commitment and structured innovation strategies are more likely to successfully implement new technologies (132). Therefore, future research should consider the impact of hospital institutional characteristics, such as ownership, and efforts to adopt innovations, such as participation in clinical trials, on the utilization of new technologies like DES-UL. However, certain facilitators might support the adoption of new technologies. For example, hospital-based HTA processes enable a more systematic assessment of novel devices, which may improve the efficiency of technology diffusion (133–135). Moreover, hospitals that participate in collaborative networks or have access to specialized funding mechanisms are more likely to implement innovations successfully (136, 137). Economic incentives and investment strategies have also been identified as crucial drivers adopting technologies in hospitals, particularly in institutions with greater financial flexibility (138, 139).

Furthermore, the introduction and widespread utilization of DES-UL are shaped by perceptions and decisions of key stakeholders, including physicians, patients, policy makers, and manufacturers. Understanding these diverse stakeholder perspectives is essential for explaining trends in procedure volumes and identifying barriers that may affect broader adoption of DES-UL. However, various factors may influence these stakeholders in their decision-making. For example, clinical guidelines often serve as the primary reference point for physicians' treatment decisions (140). In addition, other determinants play a crucial role, including status of medical care, and institutional and financial constraints (14). For patients, treatment decisions are shaped by preferences such as avoiding limb amputation and prolonging life expectancy, economic considerations such as treatment costs and the ability to return to work, and the desire to improve their quality of life (141). In the articles analyzed in our study, patient perspectives were only considered through QoL, and only in a few studies. Information on satisfaction as a patient-reported outcome (PRO) and from the physician perspective could have contributed to a more comprehensive interpretation of the case numbers development. However, satisfaction is more likely to be captured in qualitative studies (e.g., interview studies), a study design that was not included in our analysis. Policy makers set the framework for the availability and affordability of new medical technologies by establishing safety regulations, reimbursement policies, and clinical practice guidelines (142, 143). Moreover, manufacturers contribute by driving innovation, meeting regulatory requirements, and shaping market access through pricing strategies and sales activities (14, 144, 145).

It is noteworthy that slow integration of evidence into clinical guidelines is a key barrier that can delay technology adoption. In the case of DES-UL, the most recent references cited in two of the clinical guidelines are at least three years old (70, 71), which aligns with the average guideline development time (146, 147). However, in the third DES-UL guideline, the only reference given dates back eight years (72). Nevertheless, it has been suggested that minor updates to clinical guidelines could be implemented within 6-15 months (147). Understanding contributing factors and implementing targeted solutions is crucial for ensuring that future technologies are incorporated efficiently into clinical guidelines. One such factor is the presence of bureaucratic delays, which often result from complex regulatory approval processes involving multiple advisory bodies and stakeholders. Overlapping responsibilities and procedural inefficiencies can extend the timeline for guideline updates by several years. This is particularly problematic in rapidly evolving fields like vascular interventions, where evidence is generated continuously, requiring frequent updates. Another critical factor is the reliance on high-level evidence, such as RCTs, to inform guideline updates (148). This approach enhances the robustness of evidence-based decisions but can also lead to delays when available data is limited. Additionally, there is a risk that the data may be inconclusive. Conflicting clinical trial results and concerns about the generalizability of findings often contribute to delays in guideline integration (149). Furthermore, unclear recommendations, education-related challenges, and organizational inefficiencies are recurring themes in the literature on guideline implementation (150). Additionally, resistance from stakeholders, including physicians, can hinder it (151). Wang et al. (152) emphasize that health care providers may be hesitant to adopt new recommendations if they perceive them as impractical or costly, particularly when resources for training or additional infrastructure are required. Several approaches have been proposed to address these barriers. First, the development of adaptive “living” guidelines could enable continuous updates as new evidence emerges. This dynamic approach would reduce delays associated with static guideline models (153). Second, fostering early collaboration among multidisciplinary teams, including clinicians, policy makers, and patient representatives, can help achieve consensus and ensure that guidelines are relevant to diverse clinical contexts (151). Third, integrating guidelines into digital decision-support systems within electronic health records can facilitate real-time dissemination and implementation, ensuring that health care providers have immediate access to updated recommendations (154).

During the observation period, other technologies for vascular treatment remained relevant despite the overall increasing utilization of DES. For example, DCB were introduced as an alternative to DES for specific indications, such as in-stent restenosis (155, 156). Also, the development of bioresorbable vascular scaffolds (BVS) gained attention, but the technology was later criticized due to high restenosis rates (157). The results of comparative studies across various dimensions may have played a role in shaping utilization: in terms of safety, long-term studies suggested similar mortality rates between DES and DCB, though DES tended to provide longer patency and lower reintervention rates (158). However, DES were associated with a higher risk of perioperative complications, and some meta-analyses had raised concerns about Paclitaxel-based devices, although subsequent research had not confirmed a clear mortality risk (159, 160). Despite these concerns, DES-UL remain a valuable option in specific clinical contexts. At the same time, its adoption has likely been influenced not only by clinical efficacy but also by economic considerations (161, 162). Therefore, it is essential to consider cost-effectiveness analyses of DES-UL vs. alternative treatments to better understand the economic drivers behind technology utilization trends. For example, a cost-effectiveness study assessing treatments for the superficial femoral artery (SFA) compared plain old balloon angioplasty (POBA), DCB, BMS, and DES. While DES achieved the highest one-year patency rate (79%), their incremental cost per patient limb ($38,549.80) was lower than that of BMS ($59,748.85) but significantly higher than that of DCB ($14,136.10), which was ultimately identified as the most cost-effective option over a two-year horizon (161). Similarly, a modeling study evaluated endovascular interventions for SFA disease, including biomimetic stents (BioMS), DCB, and DES. The analysis found that BioMS were the most cost-effective option, whereas DCB provided the highest efficacy but at a greater cost (163). A US-based simulation model analyzing the financial impact of transitioning from BMS to DES over five years indicated that DES reduced target lesion revascularization (TLR) rates and resulted in significant cost savings when a specific implementation strategy was applied. However, their higher upfront costs required careful evaluation in terms of long-term savings (124). Additionally, a more recent study on DES variants, such as the Eluvia stent, suggests that while these devices offer superior patency, their cost-effectiveness remains dependent on reimbursement policies and the economic burden of reintervention (162). Nevertheless, DCB were considered more cost-effective and BMS remained a treatment option for certain patient groups (161, 164, 165). From these conditions and trends, generalizable lessons can be derived, which include the fact that the choice of a technology is driven not only by clinical considerations but also by economic factors. The adoption of technology is likely influenced by innovations in related technologies, which can contribute to its diffusion. Current and future trends increasingly focus on artificial intelligence (AI)-assisted clinical decision support, digital health solutions, and value-based reimbursement models, which are expected to drive the diffusion of innovative vascular technologies in the long term (166–169). The introduction of new technologies has the potential to reshape and sustainably impact markets, influencing long-term adoption dynamics.

Some limitations of our study must be mentioned. Since we could not identify full texts of all literature search hits considered potentially relevant after tiab screening, we possibly did not include all relevant scientific articles in our analysis. In addition, it is possible that not all relevant publications were found in our literature search (e.g., due to missing search terms). Furthermore, the literature search for articles ended in 08/2022. Further relevant articles may have been published after this date. In addition, we did not find all entries on relevant studies in the clinical trial registries compared to trials mentioned in the identified articles [e.g., DRASTICO (170), GUIDE SFA (171)], eventually to our search not being sensitive enough. Moreover, information on financing instruments in the USA is incomplete, as comprehensive Medicare data was not readily available and not all health care programs and insurers (e.g., Medicaid, private) were considered. We exclusively have information on technology reimbursement (MS-DRG) for 2017 onwards. But we do not have information for the time before that and our information only concerns financing mechanisms for the Medicare program. Also, it needs to be mentioned that we only considered a selection of reasons assumed to influence utilization of new technologies. Other events and factors might have been decisive regarding the development of case numbers, such as higher utilization of DES-UL due to its cost-effectiveness compared to other treatments. Additionally, the ownership of hospitals (e.g., public, private, university hospitals) and their participation in clinical trials could be decisive factors, particularly in the context of financing decisions [e.g., a German research center was involved in the SIROCCO trial (66)]. Furthermore, the study may be affected by publication bias, as it relies primarily on published studies and therefore runs the risk of overreporting positive results and underreporting negative findings (172). Moreover, the variability in study designs, endpoints, and methodologies could obscure differences in evidence quality. Finally, the study's focus on the two countries limits the generalizability of the findings to other regions.

## 5 Conclusion

It can be assumed that the development of DES-UL case numbers in hospitals in Germany and the USA is influenced by evidence and other various factors, e.g., publication of safety notices & recalls, and financing. We observed a growing body of evidence on efficacy and safety over time, i.e., the number of articles, the LoE of studies, and the study population sizes increased, as did the number of articles reporting positive results. Physicians might have used the available evidence for decision-making regarding adoption and further utilization of DES-UL. The low evidence base at the start of utilization must be viewed critically, as patients were exposed to risks while benefits remained unclear. Studies of high LoE and with large population sizes should be conducted at the beginning of the life cycles of new technologies. Therefore, health policy makers should create an environment that promotes research and development activities regarding innovations. At the same time, it must be ensured that (efficacy/effectiveness and) safety of new technologies are appropriately evaluated in a timely and regular manner. Approaches such as “coverage with evidence development” offer the opportunity to balance timely access to new technologies and gather further evidence while hospitals receive reimbursement. However, evidence should be made (easily) accessible to clinical and health decision-makers (incl. insurers) as early as possible to ensure its integration into clinical and financing decisions. In addition, clinical guideline programs should consider options for systematically incorporating emerging insights, such as through the adaptive “living” guideline model, so that the latest study results are used in the best interest of patients.

## Footnotes

^1^Federal Institute for Drugs and Medical Devices [Bundesinstitut für Arzneimittel und Medizinprodukte]. Tasks of the BfArM. Differentiation and classification. URL: https://www.bfarm.de/DE/Medizinprodukte/Aufgaben/Abgrenzung-und-Klassifizierung/_node.html, last accessed: 07/25/2024.

^2^The Implant Files. International medical devices database. URL: https://medicaldevices.icij.org/, last accessed: 07/25/2024.

^3^Institute for the Hospital Remuneration System [Institut für das Entgeltsystem im Krankenhaus]. Archive. URL: https://www.g-drg.de/archiv, last accessed: 07/25/2024.

^4^Centers for Medicare & Medicaid Services. Search. URL: https://www.cms.gov/medicare-coverage-database/search.aspx, last accessed: 02/17/2025.

^5^German Institute for Medical Documentation and Information [Deutsches Institut für Medizinische Dokumentation und Information]. Classifications. URL: https://www.dimdi.de/static/de/klassifikationen/icd/icd-10-gm/kode-suche/htmlgm2020/, last accessed: 07/25/2024.

^6^Find-a-code. The Right Code, Right Now. URL: https://www.findacode.com/, last accessed, 07/25/2024.
