# Supplementary material for: Introducing and utilizing innovative technologies in health care systems: a country comparison for peripheral drug-eluting stents in Germany and the USA
Source: Front Public Health. 2025 Jun 19;13:1488091. doi: 10.3389/fpubh.2025.1488091 (PMC12222216; doi:10.3389/fpubh.2025.1488091)
Supplement: Supplementary file 1 [file Data_Sheet_1.zip › Supplement_Material/A.1_Biomedical_databases_search_concept_strings_dates_hits.docx]

**A.1 Biomedical databases: search concept, strings, dates and hits**

**I Search concept:**

1. Table of PICO items, search components, and filter settings:

| **PICO items** | | | | | | **Filter** | **NOT component** |
| --- | --- | --- | --- | --- | --- | --- | --- |
| **Patient/problem** | | | **Intervention** | | |  |  |
| **Component 1** | **Component 2** | **Component 3** | **Component 4** | **Component 5** | **Component 6** |  |  |
| "atherosclerosis"[Mesh] OR "Constriction, Pathologic"[Mesh] OR "Peripheral Arterial Disease"[Mesh] OR "Arterial Occlusive Diseases"[Mesh] | atherosclerosis[tiab] OR constrict*[tiab] OR stenos*[tiab] OR calcific*[tiab] OR blockage[tiab] OR arterial[tiab] OR occlu*[tiab] OR vascular[tiab] | peripher*[tiab] OR femor*[tiab] OR iliac[tiab] OR genicular*[tiab] OR poplit*[tiab] OR infrapoplit*[tiab] OR peroneal[tiab] OR tibia*[tiab] OR dorsal*[tiab] OR plantar[tiab] OR leg[tiab] OR legs[tiab] OR limb*[tiab] OR extremit*[tiab] | "Drug-Eluting Stents"[Mesh] | stent*[tiab] OR scaffold*[tiab] | drug[tiab] OR Paclitaxel[tiab] OR Sirolimus[tiab] OR Everolimus[tiab] OR limus[tiab] OR Cypher[tiab] OR XIENCE*[tiab] OR drug* OR paclitaxel* OR sirolimus* OR everolimus* OR Tacrolimus* OR limus* OR agent$1 OR ingredient$1 OR substance$1 | **PubMed:**  NOT medline[sb] | ((((("Animals"[Mesh:noexp]) NOT "Humans"[Mesh])) OR Historical Article [Publication Type]) OR Editorial[Publication Type]) OR letter[Publication Type] |
|  |  |  |  |  |  | **Publication type (MEDLINE, EMBASE):**  letter OR comment OR editorial OR festschrift |  |
|  |  |  |  |  |  | **Date / time frame**  **(all databases):**  2000 - current,  2019 - current |  |

I.2 Search string using components:

- ((Component1 OR (Component2 AND Component3)) AND (Component4 OR (Component5 AND Component6))) NOT Component

I.3 Search string using search terms:

- ((("atherosclerosis"[Mesh] OR "Constriction, Pathologic"[Mesh] OR "Peripheral Arterial Disease"[Mesh] OR "Arterial Occlusive Diseases"[Mesh]) OR ((atherosclerosis[tiab] OR constrict*[tiab] OR stenos*[tiab] OR calcific*[tiab] OR blockage[tiab] OR arterial[tiab] OR occlu*[tiab] OR vascular[tiab]) AND (peripher*[tiab] OR femor*[tiab] OR iliac[tiab] OR genicular*[tiab] OR poplit*[tiab] infrapoplit*[tiab] OR peroneal[tiab] OR tibia*[tiab] OR dorsal*[tiab] OR plantar[tiab] OR leg[tiab] OR legs[tiab] OR limb*[tiab] OR extremit*[tiab]))) AND ((("Drug-Eluting Stents"[Mesh]) OR ((stent*[tiab] OR scaffold*[tiab]) AND (drug[tiab] OR Paclitaxel[tiab] OR Sirolimus[tiab] OR Everolimus[tiab] OR limus[tiab] OR Cypher[tiab] OR XIENCE*[tiab] OR drug* or paclitaxel* or sirolimus* or everolimus* or Tacrolimus* or limus* or agent$1 or ingredient$1 or substance$1)))) NOT (((((("Animals"[Mesh:noexp]) NOT "Humans"[Mesh])) OR Historical Article[Publication Type]) OR Editorial[Publication Type]) OR letter[Publication Type])

**II Search strings and dates:**

II.1.1. MEDLINE (Ovid), initial search date: 05/18/2019

| **Search** | | |
| --- | --- | --- |
| **Step** | **String** | **Hits** |
| 1 | ((femor* or iliac or genicular* or poplit* or infrapoplit* or peroneal or tibia* or dorsal* or plantar or leg$1 or limb$1 or extremit* or ?knee$1 or peripher*) adj3 arter*).ab,ti. | 70,852 |
| 2 | exp drug eluting stent/ | 10,319 |
| 3 | *stent/ | 41,887 |
| 4 | (stent* or scaffold$1).ab,ti. | 167,813 |
| 5 | 3 or 4 | 171,765 |
| 6 | (drug* or paclitaxel* or sirolimus* or everolimus* or tacrolimus* or limus*).ab,ti. | 1,523,008 |
| 7 | (coat* or elut* or cover*).ab,ti. | 583,066 |
| 8 | 5 and 6 and 7 | 14,324 |
| 9 | 1 and (2 or 8) | 700 |
| 10 | exp Animals/ not exp Humans/ | 4,580,930 |
| 11 | (letter or comment or editorial or festschrift).pt. | 1,726,738 |
| 12 | 9 not 10 not 11 | 588 |
| 13 | limit 12 to yr="2000 -Current" | 584 |

II.1.2. MEDLINE (Ovid), update search date: 08/04/2022

| **Search** | | |
| --- | --- | --- |
| **Step** | **String** | **Hits** |
| 1 | ((femor* or iliac or genicular* or poplit* or infrapoplit* or peroneal or tibia* or dorsal* or plantar or leg$1 or limb$1 or extremit* or ?knee$1 or peripher*) adj3 arter*).ab,ti. | 8,306 |
| 2 | exp drug eluting stent/ | 13,274 |
| 3 | *stent/ | 46,338 |
| 4 | (stent* or scaffold$1).ab,ti. | 219,31 |
| 5 | 3 or 4 | 223,623 |
| 6 | (drug* or paclitaxel* or sirolimus* or everolimus* or tacrolimus* or limus*).ab,ti. | 1,863,528 |
| 7 | (coat* or elut* or cover*).ab,ti. | 735,871 |
| 8 | 5 and 6 and 7 | 17,49 |
| 9 | 1 and (2 or 8) | 1,075 |
| 10 | exp Animals/ not exp Humans/ | 5,034,203 |
| 11 | (letter or comment or editorial or festschrift).pt. | 2,084,376 |
| 12 | 9 not 10 not 11 | 931 |
| 13 | limit 12 to yr="2019 -Current" | 386 |

II.2.1. EMBASE (Ovid), initial search date: 05/18/2019

| **Search** | | |
| --- | --- | --- |
| **Step** | **String** | **Hits** |
| 1 | ((femor* or iliac or genicular* or poplit* or infrapoplit* or peroneal or tibia* or dorsal* or plantar or leg$1 or limb$1 or extremit* or ?knee$1 or peripher*) adj3 arter*).ab,ti. | 92,520 |
| 2 | exp drug eluting stent/ | 31,312 |
| 3 | *stent/ | 31,145 |
| 4 | (stent* or scaffold$1).ab,ti. | 245,318 |
| 5 | 3 or 4 | 246,872 |
| 6 | (drug* or paclitaxel* or sirolimus* or everolimus* or tacrolimus* or limus*).ab,ti. | 2,029,870 |
| 7 | (coat* or elut* or cover*).ab,ti. | 697,359 |
| 8 | 5 and 6 and 7 | 26,505 |
| 9 | 1 and (2 or 8) | 1,775 |
| 10 | exp animal/ not exp human/ | 4,048,810 |
| 11 | (editorial or erratum or letter or note or patent).pt. | 2,557,502 |
| 12 | 9 not 10 not 11 | 1,631 |
| 13 | limit 12 to yr="2000 -Current" | 1,625 |

II.2.2. EMBASE (Ovid), update search date: 08/04/2022

| **Search** | | |
| --- | --- | --- |
| **Step** | **String** | **Hits** |
| 1 | ((femor* or iliac or genicular* or poplit* or infrapoplit* or peroneal or tibia* or dorsal* or plantar or leg$1 or limb$1 or extremit* or ?knee$1 or peripher*) adj3 arter*).ab,ti. | 126,146 |
| 2 | exp drug eluting stent/ | 38,245 |
| 3 | *stent/ | 33,112 |
| 4 | (stent* or scaffold$1).ab,ti. | 313,724 |
| 5 | 3 or 4 | 315,376 |
| 6 | (drug* or paclitaxel* or sirolimus* or everolimus* or tacrolimus* or limus*).ab,ti. | 2,705,390 |
| 7 | (coat* or elut* or cover*).ab,ti. | 942,008 |
| 8 | 5 and 6 and 7 | 31,529 |
| 9 | 1 and (2 or 8) | 2,507 |
| 10 | exp animal/ not exp human/ | 5,766,788 |
| 11 | (editorial or erratum or letter or note or patent).pt. | 3,126,587 |
| 12 | 9 not 10 not 11 | 2,300 |
| 13 | limit 12 to yr="2019 -Current" | 739 |

II.3.1. PubMed (NLM), initial search date: 05/27/2019

| **Search** | | |
| --- | --- | --- |
| **Step** | **String** | **Hits** |
| 1 | (femor*[tiab] OR iliac[tiab] OR genicular*[tiab] OR poplit*[tiab] OR infrapoplit*[tiab] OR peroneal[tiab] OR tibia*[tiab] OR dorsal*[tiab] OR plantar[tiab] OR leg[tiab] OR legs[tiab] OR limb*[tiab] OR extremit*[tiab] OR knee*[tiab] OR peripher*[tiab]) | 1,333,741 |
| 2 | (stent*[tiab] OR scaffold*[tiab]) | 179,020 |
| 3 | (drug*[tiab] OR paclitaxel*[tiab] OR sirolimus*[tiab] OR everolimus*[tiab] OR tacrolimus*[tiab] OR limus*[tiab]) | 1,587,682 |
| 4 | (coat*[tiab] OR elut*[tiab] OR cover*[tiab]) | 585,589 |
| 5 | #1 AND #2 AND #3 AND #4 | 992 |
| 6 | medline[sb] | 25,850,951 |
| 7 | (#5 NOT #6) | 194 |
| 8 | ("2000/01/01"[PDat] : "2019/05/24"[PDat]) | 16,236,368 |
| 9 | (#7 AND #8) | 192 |

II.3.2. PubMed (NLM), update search date: 08/04/2022

| **Search** | | |
| --- | --- | --- |
| **Step** | **String** | **Hits** |
| 1 | (femor*[tiab] OR iliac[tiab] OR genicular*[tiab] OR poplit*[tiab] OR infrapoplit*[tiab] OR peroneal[tiab] OR tibia*[tiab] OR dorsal*[tiab] OR plantar[tiab] OR leg[tiab] OR legs[tiab] OR limb*[tiab] OR extremit*[tiab] OR knee*[tiab] OR peripher*[tiab]) | 1,563,852 |
| 2 | (stent*[tiab] OR scaffold*[tiab]) | 232,876 |
| 3 | (drug*[tiab] OR paclitaxel*[tiab] OR sirolimus*[tiab] OR everolimus*[tiab] OR tacrolimus*[tiab] OR limus*[tiab]) | 1,940,286 |
| 4 | (coat*[tiab] OR elut*[tiab] OR cover*[tiab]) | 739,167 |
| 5 | #1 AND #2 AND #3 AND #4 | 1,460 |
| 6 | medline[sb] | 29,642,573 |
| 7 | (#5 NOT #6) | 212 |
| 8 | Filters: from 2019/5/24 - 3000/12/12 | 12,134,987 |
| 9 | (#7 AND #8) | 124 |

II.4.1. The Cochrane Library, initial search date: 05/22/2019

| **Search** | | |
| --- | --- | --- |
| **Step** | **String** | **Hits** |
| 1 | ((femor* or iliac or genicular* or poplit* or infrapoplit* or peroneal or tibia* or dorsal* or plantar or leg or limb or extremit* or *knee or peripher*) NEAR/3 arter*):ab,ti,kw (Word variations have been searched) | 7,569 |
| 2 | MeSH descriptor: [Drug-Eluting Stents] explode all trees | 1,255 |
| 3 | MeSH descriptor: [Stents] this term only | 2,852 |
| 4 | (stent* or scaffold):ab,ti,kw (Word variations have been searched) | 14,619 |
| 5 | #3 OR #4 | 14,619 |
| 6 | (drug* or paclitaxel* or *limus*):ab,ti,kw | 573,947 |
| 7 | (coat* or elut* or cover*):ab,ti,kw | 29,800 |
| 8 | #5 AND #6 AND #7 | 4,510 |
| 9 | #1 AND (#2 OR #8) | 271 |
| 10 | #1 AND (#2 OR #8) with Cochrane Library publication date Between Jan 2000 and May 2019, in Cochrane Reviews, Trials | 270 |
| 11 | #1 AND (#2 OR #8) with Cochrane Library publication date Between Jan 2000 and May 2019, in Trials | 264 |

II.4.2. The Cochrane Library, update search date: 08/08/2022

| **Search** | | |
| --- | --- | --- |
| **Step** | **String** | **Hits** |
| 1 | ((femor* OR iliac OR genicular* OR poplit* OR infrapoplit* OR peroneal OR tibia* OR dorsal* OR plantar OR leg OR limb OR extremit* OR *knee OR peripher*) NEAR/3 arter*):ti,ab,kw (Word variations have been searched) | 9,562 |
| 2 | MeSH descriptor: [Drug-Eluting Stents] explode all trees | 1,593 |
| 3 | MeSH descriptor: [Stents] this term only | 3,204 |
| 4 | (stent* OR scaffold*):ti,ab,kw (Word variations have been searched) | 17,749 |
| 5 | #3 OR #4 | 17,749 |
| 6 | (drug* OR paclitaxel* OR *limus*):ti,ab,kw (Word variations have been searched) | 682,449 |
| 7 | (coat* OR elut* OR cover*):ti,ab,kw (Word variations have been searched) | 37,938 |
| 8 | #5 AND #6 AND #7 | 5,576 |
| 9 | #1 AND (#2 OR #8) with Cochrane Library publication date from Apr 2019 to present, in Cochrane Reviews, Trials | 135 |
