# Supplementary material for: Introducing and utilizing innovative technologies in health care systems: a country comparison for peripheral drug-eluting stents in Germany and the USA
Source: Front Public Health. 2025 Jun 19;13:1488091. doi: 10.3389/fpubh.2025.1488091 (PMC12222216; doi:10.3389/fpubh.2025.1488091)
Supplement: Supplementary file 1 [file Data_Sheet_1.zip › Supplement_Material/A.10_Clinical_trial_registries_search_hits.docx]

**A.10 Clinical trial registries: search hits and registry content**

| **No.** | **Registry no.** | **Study title** | **Study acronym** | **Study details** | | | | | | | **Source (URL), all last accessed: 10/20/2023** |
| --- | --- | --- | --- | --- | --- | --- | --- | --- | --- | --- | --- |
|  |  |  |  | **Status** | **Date of regis-tration** | **Date of completion (estimated)** | **Conditions** | **Evaluated technologies** | **DES product name (manufacturer)** | **Country focus** |  |
| 1 | NCT00120406 | Evaluation of Zilver PTX DES in the above-the-knee femoropopliteal artery | Zilver PTX | completed | 07/11/2005 | February, 2014 | peripheral vascular diseases | DES vs. angioplasty | Zilver PTX (Cook) | USA, Germany, Japan | <https://clinicaltrials.gov/study/NCT00120406> |
| 2 | NCT00232869 | A study of SMART stent in the treatment SFA disease | SIROCCO | completed | 10/04/2005 | May, 2009 | PAD | DES vs. BMS | DES (Cordis) | Germany | <https://clinicaltrials.gov/study/NCT00232869> |
| 3 | NCT00475566 | A safety and efficacy study of Dynalink-E Everolimus eluting peripheral stent system | STRIDES | completed | 05/16/2007 | February, 2010 | atherosclerosis, peripheral vascular disease | DES vs. DES | Dynalink (Abbott) | Austria, Belgium, Germany, Italy | <https://clinicaltrials.gov/study/NCT00475566> |
| 4 | NCT01094678 | Zilver PTX global registry | none | completed | 03/25/2010 | April, 2011 | PAD, peripheral vascular disease | DES | Zilver PTX (Cook) | Germany, Italy | <https://clinicaltrials.gov/study/NCT01094678> |
| 5 | NCT01348425 | Zilver PTX drug-eluting peripheral stent study | none | completed | 04/29/2011 | September, 2012 | PAD | longer DES vs. shorter DES | Zilver PTX (Cook) | Germany, Switzer-land | <https://clinicaltrials.gov/study/NCT01348425> |
| 6 | NCT01450722 | Paclitaxel eluting stent in long SFA obstruction: a prospective, randomized comparison with bypass surgery | finnptx | recruiting | 10/07/2011 | 12/31/2022 | chronic lower limb ischemia, peripheral atherial obstructive disease, critical ischemia, claudication | Bypass and stent vs. stent | DES | Finland | <https://clinicaltrials.gov/study/NCT01450722> |
| 7 | JPRN-UMIN 000008433 | Zilver PTX DES for the femoral artery and proximal popliteal artery – prospective multicenter registry | ZEPHYR | recruitment completed | 07/13/2012 | ns | PAD | endovascular therapy | Zilver PTX (Cook) | Japan | <https://center6.umin.ac.jp/cgi-open-bin/ctr_e/ctr_view.cgi?recptno=R000009885> |
| 8 | NCT01728441 | Evaluation of Paclitaxel Eluting Stent vs Paclitaxel eluting balloon treating Peripheral artery disease of the femoral artery | REAL PTX | completed | 11/13/2012 | May, 2014 | PAD | DES vs. DCB | DES | Europe | <https://clinicaltrials.gov/study/NCT01728441> |
| 9 | JPRN-UMIN 000010071 | DES implantation vs. BMS implantation in treatment of SFA | COMBAT-ISR | completed | 02/18/2013 | ns | PAD, femoropopliteal in-stent restenosis | DES vs. BMS | ns | Japan | <https://center6.umin.ac.jp/cgi-open-bin/ctr_e/ctr_view.cgi?recptno=R000011789> |
| 10 | NCT01820637 | Stenting of the superficial femoral and/or proximal popliteal artery project | MAJESTIC | completed | 03/22/2013 | 02/22/2017 | atherosclerosis of native arteries of the extremities | drug SES | DES (Boston Scientific) | Australia, Austria, Belgium, Germany, New Zealand | <https://clinicaltrials.gov/study/NCT01820637> |
| 11 | NCT01901289 | Zilver PTX V clinical study | none | completed | 07/08/2013 | 02/15/2021 | PAD | DES | Zilver PTX (Cook) | USA | <https://clinicaltrials.gov/study/NCT01901289> |
| 12 | NCT01952457 | Cook Zilver PTX DES vs. bypass surgery for the treatment, Cook Zilver PTX DES vs. bypass surgery of femoro-popliteal TASC C&D lesions | ZILVERPASS | unknown | 09/10/2013 | December, 2019 | peripheral vascular disease | DES vs. prosthetic bypass | Zilver PTX (Cook) | Belgium | <https://clinicaltrials.gov/study/NCT01952457> |
| 13 | NCT02004951 | BMS vs. Paclitaxel eluting stent in the setting of primary stenting of intermediate length femoropopliteal lesions | BATTLE | terminated | 12/03/2013 | 08/07/2020 | PAD, femoropopliteal lesions | SES vs. DES | Zilver PTX (Cook) | France | <https://clinicaltrials.gov/study/NCT02004951> |
| 14 | NCT02033135 | Paclitaxel eluting stent or exercise for thigh atherosclerosis | PESETA | unknown | 01/08/2014 | August, 2017 | PAD | Angioplasty and DES vs. best medical treatment vs. DES | Zilver PTX (Cook) | Denmark | <https://clinicaltrials.gov/study/NCT02033135> |
| 15 | ACTRN12614000504617 | Clinical evaluation of the thumbwheel delivery Zilver PTX drug-eluting peripheral stent for treatment of lesions of the above-the-knee femoropopliteal artery | none | completed | 05/13/2014 | ns | PAD, diseases of vasculature and circulation incl. lymphatic system, cardiovascular | DES | Zilver PTX (Cook) | Australia, Germany, New Zealand | <https://anzctr.org.au/ACTRN12614000504617.aspx> |
| 16 | NCT02171962 | Zilver PTX in China | none | completed | 06/18/2014 | 02/09/2017 | PAD | DES | Zilver PTX (Cook) | China | <https://clinicaltrials.gov/study/NCT02171962> |
| 17 | NCT02254837 | Zilver PTX post-market study in Japan | none | completed | 09/24/2014 | 06/30/2018 | PAD | DES | Zilver PTX (Cook) | ns | <https://clinicaltrials.gov/study/NCT02254837> |
| 18 | NCT02271529 | Zilver PTX delivery system | none | completed | 10/17/2014 | May, 2015 | peripheral vascular disease | DES | Zilver PTX (Cook) | Australia, Germany, New Zealand | <https://clinicaltrials.gov/study/NCT02271529> |
| 19 | NCT03671655 | Excellence in peripheral arterial disease treatment of superficial femoral artery disease with DES | XLPAD DES SFA | terminated | 10/21/2014 | 08/31/2015 | peripheral vascular diseases, chronic total occlusion of artery of the extremities | DES vs. BMS | DES | USA | <https://clinicaltrials.gov/study/NCT03671655> |
| 20 | NCT02574481 | Eluvia vs. Zilver PTX DES | IMPERIAL | completed | 09/22/2015 | 04/12/2022 | atherosclerosis of native arteries of the extremities | DES vs. DES | Eluvia (Boston Scientific) | USA, Austria, Belgium, Canada, Germany, Japan, New Zealand | <https://clinicaltrials.gov/study/NCT02574481> |
| 21 | NCT02701881 | Comparison of the primary long vs. short coverage with DES for long femoropopliteal artery disease: investigator-initiated clinical study | PARADE II | unknown | 02/29/2016 | August, 2021 | femoropopliteal artery disease | longer DES vs. spot stenting DES | Zilver PTX (Cook) | (South-) Korea | <https://clinicaltrials.gov/study/NCT02701881> |
| 22 | NCT02734836 | DES for the management of peripheral arterial disease of the SFA | DESPERADO | not recruiting | 04/06/2016 | 10/02/2018 | PAD | DES | Zilver PTX (Cook) | USA | <https://clinicaltrials.gov/show/NCT02734836> |
| 23 | NCT02921230 | Trial comparing Eluvia DES vs. BMS in treatment of superficial femoral and/or proximal popliteal artery | EMINENT | active (not recruiting) | 09/06/2016 | April, 2025 | arterial occlusive diseases, atherosclerosis, vascular diseases, arteriosclerosis | DES | Eluvia (Boston Scientific) | Austria, Belgium, France, Germany, Ireland, Italy, Nether-lands, Spain, Switzer-land, UK | <https://clinicaltrials.gov/study/NCT02921230> |
| 24 | NCT02936622 | Paclitaxel-coated peripheral stents used in the treatment of femoropopliteal stenoses | XPEDITE | active (not recruiting) | 10/12/2016 | January, 2024 | PAD | DES vs. slower-dissolving polymer-free DES vs. higher-dose polymer-free DES | Zilver PTX (Cook) | Germany, New Zealand | <https://clinicaltrials.gov/study/NCT02936622> |
| 25 | NCT03037411 | A real world evaluation of the Eluvia stent in subjects with lesions located in the femoropopliteal arteries | REGAL | completed | 12/14/2016 | 03/24/2022 | arterial occlusive diseases, atherosclerosis, vascular diseases, arteriosclerosis | DES | Eluvia (Boston Scientific) | Austria, Belgium, France, Italy, Spain | <https://clinicaltrials.gov/study/NCT03037411> |
| 26 | NCT03510676 | The ILLUMINA study | ILLUMINA | completed | 04/04/2018 | 03/07/2019 | PAD | DES | NiTiDES (Alvimedica) | France, Germany, Italy | <https://clinicaltrials.gov/study/NCT03510676> |
| 27 | NCT03505931 | Korean multicenter registry of Eluvia stent for femoropopliteal artery disease | K-ELUVIA | not recruiting | 04/13/2018 | April, 2021 | symptomatic PAD with femoropopliteal lesions, moderate or severe claudication, critical limb ischemia | DES | Eluvia (Boston Scientific) | (South-) Korea | <https://clinicaltrials.gov/show/NCT03505931> |
| 28 | JPRN-UMIN 000036174 | Usage of contemporary DES by Japanese interventional radiologists for femoropopliteal in-stent restenosis lesions - prospective multicenter study | COMBAT-ISR | recruitment completed | 03/12/2019 | 03/31/2021 | femoropopliteal in-stent restenosis | DES | Eluvia (Boston Scientific) | Japan | <https://center6.umin.ac.jp/cgi-open-bin/ctr_e/ctr_view.cgi?recptno=R000041202> |
| 29 | JPRN-UMIN 000036798 | Real DES study – Zilver PTX vs. Eluvia DES for femoropopliteal disease in multicenter prospective study | Real DES | recruiting | 05/20/2019 | ns | obstructive arteriosclerosis of SFA lesion | DES vs. DES | Zilver PTX (Cook), Eluvia (Boston Scientific) | Japan | <https://center6.umin.ac.jp/cgi-open-bin/ctr_e/ctr_view.cgi?recptno=R000041929> |
| 30 | JPRN-UMIN 000036499 | Vascular response to implantation of DES in femoropopliteal artery lesions | none | preinitiation | 07/01/2019 | ns | PAD | DES | ns | Japan | <https://center6.umin.ac.jp/cgi-open-bin/ctr_e/ctr_view.cgi?recptno=R000041580> |
| 31 | NCT04204564 | VISION and VQI Paclitaxel safety analysis | VISION-VQI | unknown | 12/12/2019 | 06/01/2020 | PAD | DCB vs. DES | Zilver PTX (Cook) | ns | <https://clinicaltrials.gov/study/NCT04204564> |
| 32 | NCT04674969 | Drug-eluting registry: real-world treatment of lesions in the peripheral vasculature | ELEGANCE | recruiting | 12/01/2020 | January, 2028 | peripheral vascular diseases | DES vs. other drug-eluting devices | DES (Boston Scientific) | USA, Austria, Australia, Canada, China, France, Germany, Spain, Taiwan, Thailand | <https://clinicaltrials.gov/study/NCT04674969> |
| 33 | NCT05296031 | Zilver PTX DES vs. Zilver Flex BMS | none | completed | 03/07/2022 | 08/31/2019 | vascular diseases | DES vs. BMS | Zilver PTX (Cook) | ns | <https://clinicaltrials.gov/study/NCT05296031> |
| 34 | NCT05575245 | DES vs. Excimer laser ablation combined drug-coated balloon to treat arteriosclerosis occlusive disease of lower extremity | none | not recruiting | 08/10/2022 | 10/20/2024 | atherosclerosis, ischemia | DES vs. Excimer laser ablation and DCB | ns | China | <https://clinicaltrials.gov/show/NCT05575245> |
| 35 | NCT05522218 | Eluvia DES for the patients with femoropopliteal artery lesions | none | recruiting | 08/24/2022 | 12/31/2025 | vascular diseases, stent complication | DES | Eluvia (Boston Scientific) | China | <https://clinicaltrials.gov/study/NCT05522218> |
| 36 | NCT05780359 | Evaluating the safety and efficacy of the G-stream DES in the above-the-knee femoropopliteal artery | G-streamPAD | recruiting | 03/08/2023 | 08/31/2026 | PAD | DES vs. DCB | G-stream (Alain Medical) | China | <https://clinicaltrials.gov/study/NCT05780359> |
| 37 | ChiCTR2300076236 | Efficacy of drug-coated BMS vs. heparin-coated overlay stents in lower extremity femoropopliteal artery lesions: a randomized controlled trial | ELITE | recruiting | 09/27/2023 | 12/01/2026 | atherosclerotic femoropopliteal occlusive disease | drug-coated BMS vs. Heparin-coated covered stent | Eluvia (Boston Scientific) | China | <https://www.chictr.org.cn/showproj.html?proj=197524> |
| **Legend:** BMS – bare metal stent, DCB – drug-coated balloon, DES – drug-eluting stent, ns – not stated, PAD – peripheral artery disease, SFA – superficial femoral artery, TASC C&D – Trans-Atlantic Inter-Society Consensus C and D (classification of complex and extensive arterial lesions of the lower extremities, where surgical treatment (revascularization) is generally preferred), vs. – versus | | | | | | | | | | | |
