# Supplementary material for: Introducing and utilizing innovative technologies in health care systems: a country comparison for peripheral drug-eluting stents in Germany and the USA
Source: Front Public Health. 2025 Jun 19;13:1488091. doi: 10.3389/fpubh.2025.1488091 (PMC12222216; doi:10.3389/fpubh.2025.1488091)
Supplement: Supplementary file 1 [file Data_Sheet_1.zip › Supplement_Material/A.11_Clinical_guidelines_search_hits.docx]

**A.11 Clinical guidelines: search hits and guideline content**

| **Reference & background** | | | **Targeted technology** | | **Recommendation** | | **Source (URL), all last accessed: 10/31/2023** |
| --- | --- | --- | --- | --- | --- | --- | --- |
| **Author (year) / institution** | **Title** | **Country focus** | **DES technology (product name)** | **Indication** | **Level of evidence (LoE)*, number of articles cited in clinical guideline (reference(s)) / grade of recommendation (GoR)** | **Statement** |  |
| Lawall et al. (2015) (1) / German Society for Angiology – Society for Vascular Medicine [Deutsche Gesellschaft für Angiologie – Gesellschaft für Gefäßmedizin] | S3 guideline on diagnosis, therapy and follow-up of peripheral arterial occlusive disease [S3-Leitlinie zur Diagnostik, Therapie und Nachsorge der peripheren arteriellen Verschluss-krankheit]. | Germany | Zilver PTX, S.M.A.R.T.  (+ Sirolimus) | peripheral arterial occlusive disease | Ib & IV, n=4 (2–5) / ns | no adequate assessment of clinical relevance possible after consensus decision | [https://register.awmf.org/assets/ guidelines/065-003l_S3_PAVK_ periphere_arterielle_Verschluss krankheit_2020-05.pdf](https://register.awmf.org/assets/guidelines/065-003l_S3_PAVK_periphere_arterielle_Verschlusskrankheit_2020-05.pdf) |
| Frank et al. (2019) (6) / European Society for Vascular Medicine (ESVM) | Guideline on peripheral  arterial disease. Volume 48. Supplement 102 / 2019. | Europe | Zilver PTX | peripheral arterial disease | Ib, n=2 (5, 7) / ns | study results represent a risk reduction for restenosis and target lesion revascularization through 5 years in comparison with provisional BMS | [https://www.vascular-medicine.org /wp-content/uploads/2021/04/PAD -Guideline.pdf](https://www.vascular-medicine.org/wp-content/uploads/2021/04/PAD-Guideline.pdf) |
| Conte et al. (2019) (8) / European Society for Vascular Surgery (ESVS) | Global vascular guidelines on the management of chronic limb-threatening ischemia. | Europe | Zilver PTX | chronic limb-threatening ischemia | Ib, n=1 (5) /  B (moderate) &  2 (weak) | drug-eluting technologies to be considered as adjuncts to balloon angioplasty | [https://www.ejves.com/action/ showPdf?pii=S1078-5884%2819%2930380-6](https://www.ejves.com/action/showPdf?pii=S1078-5884%2819%2930380-6) |
| **Legend:** * allocation to level of evidence (LoE) according to our assessment of identified primary studies, Ib – randomized controlled trial (RCT), DES – drug-eluting stent, ns – not stated; evaluation of technology in guideline: positive, neutral/unclear | | | | | | | |

**References**

1. Lawall H, Zemmrich C. *S3 guideline on diagnosis, therapy and follow-up of peripheral arterial occlusive disease [S3-Leitlinie zur Diagnostik, Therapie und Nachsorge der peripheren arteriellen Verschlusskrankheit]* (2015). 168 p.

2. Duda SH, Bosiers M, Lammer J, Scheinert D, Zeller T, Oliva V, et al. Drug-eluting and bare nitinol stents for the treatment of atherosclerotic lesions in the superficial femoral artery: long-term results from the SIROCCO trial. *Journal of Endovascular Therapy* (2006) **13**:701–10. doi:10.1583/05-1704.1

3. Bosiers M, Peeters P, Tessarek J, Deloose K, Strickler S. The Zilver PTX single arm study: 12-month results from the TASC C/D lesion subgroup. *Journal of Cardiovascular Surgery* (2013) **54**:115–22.

4. Dake MD, Scheinert D, Tepe G, Tessarek J, Fanelli F, Bosiers M, et al. Nitinol stents with polymer-free Paclitaxel coating for lesions in the superficial femoral and popliteal arteries above the knee: twelve-month safety and effectiveness results from the Zilver PTX single-arm clinical study. *Journal of Endovascular Therapy* (2011) **18**:613–23. doi:10.1583/11-3560.1

5. Dake MD, Ansel GM, Jaff MR, Ohki T, Saxon RR, Smouse HB, et al. Paclitaxel-eluting stents show superiority to balloon angioplasty and bare metal stents in femoropopliteal disease: twelve-month Zilver PTX randomized study results. *Circulation: Cardiovascular Interventions* (2011) **4**:495–504. doi:10.1161/CIRCINTERVENTIONS.111.962324

6. Frank U, Nikol S, Belch J. *Guideline on peripheral arterial disease. Volume 48. Supplement 102* (2019). 80 p.

7. Dake MD, Ansel GM, Jaff MR, Ohki T, Saxon RR, Smouse HB, et al. Durable clinical effectiveness with Paclitaxel-eluting stents in the femoropopliteal artery: 5-year results of the Zilver PTX randomized trial. *Circulation* (2016) **133**:1472–83. doi:10.1161/CIRCULATIONAHA.115.016900

8. Conte MS, Bradbury AW, Kolh P, White JV, Dick F, Fitridge R, et al. Global Vascular Guidelines on the Management of Chronic Limb-Threatening Ischemia. *European journal of vascular and endovascular surgery the official journal of the European Society for Vascular Surgery* (2019) **58**:S1-S109.e33. doi:10.1016/j.ejvs.2019.05.006
