# Supplementary material for: Introducing and utilizing innovative technologies in health care systems: a country comparison for peripheral drug-eluting stents in Germany and the USA
Source: Front Public Health. 2025 Jun 19;13:1488091. doi: 10.3389/fpubh.2025.1488091 (PMC12222216; doi:10.3389/fpubh.2025.1488091)
Supplement: Supplementary file 1 [file Data_Sheet_1.zip › Supplement_Material/A.12_Safety_notices_and_recalls.docx]

**A.12 Safety notices and recalls: overview and content, sources, and search terms**

**I Overview and content**

| **Product name*** | **Date** | **Type** | **Content** | **Country focus** | **Source (URL), all last accessed: 07/11/2023** |
| --- | --- | --- | --- | --- | --- |
| Zilver PTX | 01/14/2013 | safety notice | - changes and corrections to labels and labeling - **serious adverse event:** catheter rupture of the delivery system, where the tip breaks off after the stent has been inserted into the patient's body | Poland | <https://medicaldevices.icij.org/events/pol-peripheral-stent-zilver-ptx-drug-eluting-peripheral-stent> |
| Zilver PTX | 01/22/2013 | recall | ns | Spain | <https://medicaldevices.icij.org/events/esp-peripheral-drug-eluting-stent-zilver-ptx> |
| Zilver PTX | 02/08/2013 | safety notice | - **serious adverse event:** breakage of the tip of the stent delivery catheter system after stent release with separation of the tip | Germany | <https://www.bfarm.de/SharedDocs/Kundeninfos/DE/11/2013/08008-12_kundeninfo_de.pdf?__blob=publicationFile&v=4> |
| Zilver PTX | 04/18/2013 | recall | - fractures of the delivery system inner catheter after stent deployment, and separation of the inner catheter tip section - **adverse events**, including one death, occurred in cases with tip separation | USA | <https://medicaldevices.icij.org/events/usa-zilver-ptx-drugeluting-peripheral-stent> |
| Zilver PTX | 04/19/2013 | safety notice | - complaint reports relating to the delivery system for the Zilver PTX drug eluting stent - **serious adverse events:** involving fractures of the delivery system inner catheter after stent deployment, and separation of the inner catheter tip section - higher than expected potential of inner delivery catheter breakage due to inconsistencies in the catheter manufacturing process; this is a potential failure mode of the delivery system and does not affect the safety or efficacy of the implantable stent | Hong Kong | <https://medicaldevices.icij.org/events/hkg-zilver-ptx-drug-eluting-peripheral-stent> |
| Zilver PTX | 04/22/2013 | recall | - complaints: fractures of the delivery system inner catheter after stent deployment, and separation of the inner catheter tip section | New Zealand | <https://medicaldevices.icij.org/events/nzl-cook-zilver-ptx-drug-eluting-peripheral-stent> |
| Zilver PTX | 04/26/2013 | recall |  | Australia | <https://medicaldevices.icij.org/events/aus-zilver-ptx-drug-eluting-peripheral-stent> |
| Zilver PTX | 05/06/2013 | safety notice |  | Germany | <https://www.bfarm.de/SharedDocs/Kundeninfos/DE/11/2013/02316-13_kundeninfo_de.pdf?__blob=publicationFile> |
| Zilver PTX | 05/09/2013 | recall | ns | Spain | <https://medicaldevices.icij.org/events/esp-peripheral-drug-eluting-stent-zilver-ptx-632353d1-a6ed-4227-8ea7-9d0f9e0c643d> |
| Zilver PTX | 05/16/2013 | recall | ns | France | <https://medicaldevices.icij.org/events/fra-zilver-ptx-drug-eluting-device-stent> |
| Zilver PTX | 06/18/2020 | safety notice | - evidence of increased risk of late mortality after use of paclitaxel-eluting stents for peripheral arterial occlusive disease at approximately 2-3 years after treatment [Katsanos et al. (2018)]** | Germany | <https://www.bfarm.de/SharedDocs/Kundeninfos/DE/07/2020/08161-20_kundeninfo_de.pdf?__blob=publicationFile> |
| Eluvia | 11/06/2017 | safety notice | - partial stent deployment - part of the stent can become anchored in the vessel while the rest of the stent remains within the delivery system - most common reported injury has been additional medical or minor surgical intervention, vessel trauma or prolongation of the implant procedure - cases of major surgery to retrieve the stent/delivery system or to correct vascular compromise | Germany | <https://www.bfarm.de/SharedDocs/Kundeninfos/DE/11/2017/10918-17_kundeninfo_de.pdf?__blob=publicationFile&v=1> |
| Eluvia | 11/07/2017 | recall |  | Australia | <https://medicaldevices.icij.org/events/aus-eluvia-150mm-and-innova-200mm-stent-systems> |
| Eluvia | 11/10/2017 | safety notice |  | Poland | <https://medicaldevices.icij.org/events/pol-eluvia-150-mm-and-innova-180-mm-and-200-mm-stent-systems> |
| Eluvia | 11/13/2017 | recall | - ns | France | <https://medicaldevices.icij.org/events/fra-eluvia-150mm-and-innova-180mm-and-200mm-peripheral-stents> |
| Eluvia | 11/17/2017 | safety notice | - partial deployment - stent cannot be fully released from the delivery system - portion of the stent may become trapped in the blood vessel while the remainder stays within the delivery system | Netherlands | <https://medicaldevices.icij.org/events/nld-eluviatm-150-mm-en-innovatm-180-mm-en-200-mm-stentsystemen> |
| Eluvia | 06/18/2020 | safety notice | - evidence of increased risk of late mortality after use of paclitaxel-eluting stents for peripheral arterial occlusive disease at approximately 2-3 years after treatment [Katsanos et al. (2018)]** | Germany | <https://www.bfarm.de/SharedDocs/Kundeninfos/DE/07/2020/08161-20_kundeninfo_de.pdf?__blob=publicationFile> |
| S.M.A.R.T.*** | 08/22/2012 | recall | - may have sterile barrier impairments due to holes or tears on the packaging of certain lots | Germany | <https://www.bfarm.de/SharedDocs/Kundeninfos/DE/11/2012/02503-12_kundeninfo_de.pdf?__blob=publicationFile> |
| Cypher**** | 10/30/2003 | safety notice | - reactions of subacute thrombosis (sat) and hypersensitivity with the use of the cypher cordis coronary stent - associated with patient death and with injuries / injuries in the patient who required medical or surgical intervention - hypersensitivity reaction symptoms include: pain, hives, respiratory changes, fever, itching, and changes in blood pressure | Brazil | <https://medicaldevices.icij.org/events/bra-coronary-stents-cypher-sirolimus-eluting> |
| Xience V**** | 05/11/2009 | safety notice | - reports concerning the distal shaft of the catheter delivery system that has exhibited damage | Germany | <https://www.bfarm.de/SharedDocs/Kundeninfos/EN/11/2009/01103-09_kundeninfo_en.html> |
| **Legend:** ns – not stated / no report of reasons for safety notice or recall found; * order by date of publication, mode (drug-eluting stent, self-expanding stent, bare metal stent (BMS)), and site of action (upper leg, coronary); ** Katsanos K, Spiliopoulos S, Kitrou P, Krokidis M, Karnabatidis D. Risk of death following application of Paclitaxel-coated balloons and stents in the femoropopliteal artery of the leg: a systematic review and meta-analysis of randomized controlled trials. J Am Heart Assoc (2018) 7:e011245. doi:10.1161/JAHA.118.011245; *** originally a BMS coated with drug for study purposes; **** originally a coronary stent used off-label for study purposes | | | | | |

**II. Sources, and search terms**

II.1. Federal Institute for Drugs and Medical Devices [Bundesinstitut für Arzneimittel und Medizinprodukte (BfArM)] website (<https://www.bfarm.de/EN/Home/_node.html>, latest search: 07/08/2023):

1. Eluvia
2. Zilver
3. S.M.A.R.T. stent
4. Dynalink stent
5. Cypher stent
6. Taxus stent
7. Xience V
8. Medikament AND Stent
9. Drug-eluting stent
10. Stent AND Oberschenkel
11. Stent AND femoral

II.2 The Implant Files website (<https://medicaldevices.icij.org/>, latest search: 07/08/2023):

1. Eluvia
2. Zilver PTX
3. S.M.A.R.T.
4. Dynalink
5. Cypher
6. Taxus Liberté
7. Xience V
8. drug-eluting stent AND upper leg
9. drug-eluting stent AND femoral
