# Supplementary material for: Introducing and utilizing innovative technologies in health care systems: a country comparison for peripheral drug-eluting stents in Germany and the USA
Source: Front Public Health. 2025 Jun 19;13:1488091. doi: 10.3389/fpubh.2025.1488091 (PMC12222216; doi:10.3389/fpubh.2025.1488091)
Supplement: Supplementary file 1 [file Data_Sheet_1.zip › Supplement_Material/A.13_Market_approval_dates.docx]

**A.13 Market approval: overview of products’ CE certification and FDA approval dates**

| **Product name (manufacturer)*** | **Europe/Germany: date of CE certification** | **Source (URL), last accessed;**  **or publication** | **USA: date of FDA approval** | **Source (URL), last accessed** |
| --- | --- | --- | --- | --- |
| Zilver PTX (Cook Medical, USA) –  **DES-UL** | 08/12/2009** | <https://www.biospace.com/article/releases/cook-medical-announces-ce-mark-approval-and-european-launch-of-the-zilver-r-ptx-r-stent-/>, 01/08/2024 | 11/14/2012 | <https://www.accessdata.fda.gov/cdrh_docs/pdf10/P100022B.pdf>, 01/08/2024 |
| Eluvia (Boston Scientific, USA) –  **DES-UL** | 02/21/2016** | <https://www.tctmd.com/news/boston-scientific-receives-ce-mark-eluviatm-drug-eluting-vascular-stent-and-announces>, 01/08/2024 | 09/18/2018 | <https://www.accessdata.fda.gov/cdrh_docs/pdf18/P180011B.pdf>, 01/08/2024 |
| Dynalink-E (Abbott Medical Devices, USA) – **drug-eluting SES-UL** | CE certification is not pursued | Zechmeister-Koss I, Fischer S. Drug-eluting stents for peripheral arterial disease [Medikamentenfreisetzende Stents bei peripherer arterieller Verschlusskrankheit]. Decision Support Document No. 75; 2014. Wien: Ludwig Boltzmann Institute for Health Technology Assessment. URL: <https://eprints.aihta.at/1032/1/DSD_75.pdf>, 01/08/2024 | clinical investigation is still ongoing | <https://www.rivm.nl/bibliotheek/rapporten/360050024.pdf>, 01/08/2024 |
| NiTiDes (Alvimedica, Turkey) – **drug-eluting SES-UL** | 05/31/2021** | <https://evtoday.com/news/alvimedicas-nitides-amphilimus-eluting-stent-receives-ce-mark-approval>, 01/08/2024 | no information found | - |
| G-stream (Alain Medical, China) –  **drug-eluting SES-UL** | no information found | - | no information found | - |
| S.M.A.R.T. (Cordis / Johnson & Johnson, USA) – **self-expanding** **BMS (coronary**, UL***)** | probably no CE certification | Zechmeister-Koss I, Fischer S. Drug-eluting stents for peripheral arterial disease [Medikamentenfreisetzende Stents bei peripherer arterieller Verschlusskrankheit]. Decision Support Document No. 75; 2014. Wien: Ludwig Boltzmann Institute for Health Technology Assessment. URL: <https://eprints.aihta.at/1032/1/DSD_75.pdf>, 01/08/2024 | 08/12/2003*** | <https://www.accessdata.fda.gov/cdrh_docs/pdf2/P020036b.pdf>, 01/08/2024 |
|  |  |  | 11/08/2012**** | <https://www.jnj.com/media-center/press-releases/smart-vascular-stent-systems-receive-fda-approval-for-use-in-sfa>, 01/08/2024 |
| Cypher (Cordis / Johnson & Johnson, USA) – **coronary DES** | 05/15/2002** | <https://johnsonandjohnson.gcs-web.com/news-releases/news-release-details/cordis-cyphertm-sirolimus-eluting-stent-receives-ce-mark/>, 07/08/2023 | 04/24/2003** | <https://johnsonandjohnson.gcs-web.com/static-files/f7c74945-1dd7-4eef-87b6-47c0d4ea828b>, 07/08/2023 |
| Taxus Liberté (Boston Scientific, USA) – **coronary DES** | 12/26/2007** | <https://www.dicardiology.com/content/taxus-liberte-des-receives-ce-mark-use-diabetic-patients>, 01/08/2024 | 10/10/2008** | <https://www.dicardiology.com/product/fda-approves-boston-scientifics-second-generation-taxus-liberte-des>, 01/08/2024; <https://news.bostonscientific.com/news-releases?item=59094>, 01/08/2024 |
| Xience V (Abbott Medical Devices, USA) – **coronary DES** | 03/11/2008** | <https://www.dicardiology.com/content/abbotts-225-mm-xience-v-stent-gets-ce-mark-approval>, 01/08/2024 | 07/03/2008** | <https://www.meddeviceonline.com/doc/fda-approves-abbotts-xience-v-drug-eluting-0001>, 07/08/2023 |
| **Legend:** BMS – bare metal stent; CE – Conformité Européene; DES – drug-eluting stent; FDA – Food and Drug Administration; SES – self-expanding stent; UL – upper leg; * order by date of approval, mode (DES, SES, BMS), and site of action (UL, coronary); ** date of source publication; *** indication area: coronary; **** indication areas: superficial femoral artery (SFA) and proximal popliteal artery (PPA) | | | | |
