# Supplementary material for: Introducing and utilizing innovative technologies in health care systems: a country comparison for peripheral drug-eluting stents in Germany and the USA
Source: Front Public Health. 2025 Jun 19;13:1488091. doi: 10.3389/fpubh.2025.1488091 (PMC12222216; doi:10.3389/fpubh.2025.1488091)
Supplement: Supplementary file 1 [file Data_Sheet_1.zip › Supplement_Material/A.15._Market_approval_development_description.docx]

**A.15 Market approval: detailed description of the development of the products’ market approval over time**

We identified a total of three drug-eluting stent (DES) products approved for utilization in the upper leg (UL): Zilver PTX (manufacturer: Cook Medical, USA), Eluvia (Boston Scientific, USA), and Dynalink-E (Abbott Medical Devices, USA). The Zilver PTX stent was the first DES-UL CE certified for the EU market in 2009 (1) and approved by the FDA for the USA three years later (in 2012) (2). The Eluvia stent was CE certified in 2016 (3). The FDA approval followed in 2018 (4). The Dynalink-E stent is a drug-eluting self-expanding stent (SES) investigated in only one study. The results were published in 2011 (5). We did not find dates for FDA approval. For the EU, we found information from 2011 that CE certification of the Dynalink-E stent has not been pursued further (6). The other DES that were investigated in the studies are stents approved in the EU and USA for utilization in coronary vessels: Cypher (Cordis / Johnson & Johnson, USA) (7, 8), Taxus Liberté (Boston Scientific, USA) (9, 10), and Xience V (Abbott Medical Devices, USA) (11, 12). Another stent reported in the identified articles is the S.M.A.R.T. stent (Cordis / Johnson & Johnson, USA), originally developed as a self-expanding bare metal stent (BMS) for the UL, but coated with the drug Sirolimus for study purposes (13). The S.M.A.R.T. stent had already been approved as a BMS in the USA for utilization in the iliac artery in 2003 (14), and was also approved in 2012 for utilization in the superficial femoral artery and proximal popliteal artery indications (15). According to the information we have found, it can be assumed that the S.M.A.R.T. stent has not received CE certification (6). However, we identified the new drug-eluting SES-UL NiTiDES (Alvimedica, Turkey) as part of our research in clinical trial registries (16, 17). The NiTiDES stent was CE certified in 2021 (18). We have not found any information on its market approval in the USA. In the clinical trial registries, we also identified information on another drug-eluting SES-UL named G-stream (Alain Medical, China) (19). Unfortunately, we could not find any approval data for this product. An overview of information regarding the DES-UL products’ market approval in the EU and the USA, including manufacturer names and countries, and a graphical representation of the products’ market approval dates over time alongside the case number curves is given in **Appendix A.13 & A.14**.

**References**

1. Biospace.com. *Cook Medical announces CE mark approval and European launch of the Zilver PTX stent* [cited 2024 Jan 08]. Available from: https://www.biospace.com/article/releases/cook-medical-announces-ce-mark-approval-and-european-launch-of-the-zilver-r-ptx-r-stent-/

2. U.S. Food & Drug Administration. *Summary of Safety and Effectiveness Data (SSED). I. General information; device generic name: drug-eluting peripheral, stent device trade name: Zilver PTX drug-eluting peripheral stent* (2012) [cited 2024 Aug 01]. Available from: https://www.accessdata.fda.gov/cdrh_docs/pdf10/P100022B.pdf

3. tctmd.com. *Boston Scientific receives CE mark for Eluvia drug-eluting vascular stent and announces initiation of new clinical trial* (2016) [cited 2024 Jan 08]. Available from: https://www.tctmd.com/news/boston-scientific-receives-ce-mark-eluviatm-drug-eluting-vascular-stent-and-announces

4. U.S. Food & Drug Administration. *Summary of Safety and Effectiveness Data (SSED). I. General information; device generic name: stent, superficial femoral artery, drug eluting; device trade name: ELUVIA drug-eluting vascular stent system* (2018) [cited 2024 Jan 08]. Available from: https://www.accessdata.fda.gov/cdrh_docs/pdf18/P180011B.pdf

5. Lammer J, Bosiers M, Zeller T, Schillinger M, Boone E, Zaugg MJ, et al. First clinical trial of nitinol self-expanding everolimus-eluting stent implantation for peripheral arterial occlusive disease. *Journal of Vascular Surgery* (2011) **54**:394–401. doi:10.1016/j.jvs.2011.01.047

6. Zechmeister-Koss I, Fischer S. *Drug-eluting stents for peripheral arterial occlusive disease [Medikamentenfreisetzende Stents bei peripherer arterieller Verschlusskrankheit]*. Decision support document 75. Wien (2014).

7. Johnson&Johnson. *Cordis' CYPHER Sirolimus-eluting stent receives CE mark* (2002) [cited 2023 Jul 08]. Available from: https://johnsonandjohnson.gcs-web.com/news-releases/news-release-details/cordis-cyphertm-sirolimus-eluting-stent-receives-ce-mark/

8. Johnson&Johnson. *FDA approves landmark treatment for coronary artery disease* (2003) [cited 2023 Jul 08]. Available from: https://johnsonandjohnson.gcs-web.com/static-files/f7c74945-1dd7-4eef-87b6-47c0d4ea828b

9. Dicardiology.com. *TAXUS Liberte DES receives CE mark for use in diabetic patients* (2007) [cited 2024 Jan 08]. Available from: https://www.dicardiology.com/content/taxus-liberte-des-receives-ce-mark-use-diabetic-patients

10. Dicardiology.com. *FDA approves Boston Scientific's second-generation TAXUS Liberte DES* (2008) [cited 2024 Jan 08]. Available from: https://www.dicardiology.com/product/fda-approves-boston-scientifics-second-generation-taxus-liberte-des

11. Dicardiology.com. *Abbott's 2.25 mm XIENCE V stent gets CE mark approval* (2008) [cited 2024 Jan 08]. Available from: https://www.dicardiology.com/content/abbotts-225-mm-xience-v-stent-gets-ce-mark-approval

12. Meddeviceonline.com. *FDA approves Abbott's XIENCE V drug eluting stent* (2008) [cited 2024 Jan 08]. Available from: https://www.meddeviceonline.com/doc/fda-approves-abbotts-xience-v-drug-eluting-0001

13. Duda SH, Bosiers M, Lammer J, Scheinert D, Zeller T, Tielbeek A, et al. Sirolimus-eluting versus bare nitinol stent for obstructive superficial femoral artery disease: the SIROCCO II trial. *Journal of Vascular and Interventional Radiology* (2005) **16**:331–8. doi:10.1097/01.RVI.0000151260.74519.CA

14. U.S. Food & Drug Administration. *Summary of Safety and Effectiveness Data (SSED). I. General information; device generic name: iliac stent, device trade name: S.M.A.R.T. nitinol stent system, and S.M.A.R.T. Control nitinol stent system* (2003) [cited 2024 Jan 08]. Available from: https://www.accessdata.fda.gov/cdrh_docs/pdf2/P020036b.pdf

15. Johnson&Johnson. *S.M.A.R.T.® Vascular Stent Systems Receive FDA approval for use in SFA. First stent in the U.S. with both SFA and Iliac indications* (2012) [cited 2024 Jan 08]. Available from: https://www.jnj.com/media-center/press-releases/smart-vascular-stent-systems-receive-fda-approval-for-use-in-sfa

16. Clinicaltrials.gov. *The ILLUMINA study (ILLUMINA). Clinicaltrials.gov ID NCT03510676* (2018) [cited 2024 Jan 08]. Available from: https://clinicaltrials.gov/study/NCT03510676

17. Alvimedica. *Femoro-popliteal region / DES S.E. - NiTiDES* (2018) [cited 2024 Jan 08]. Available from: https://www.alvimedica.com/Product/1033/des-s-e-nitides

18. evtoday.com. *Alvimedica’s NiTides amphilimus-eluting stent receives CE mark approval* (2021) [cited 2024 Jan 08]. Available from: https://evtoday.com/news/alvimedicas-nitides-amphilimus-eluting-stent-receives-ce-mark-approval

19. Clinicaltrials.gov. *Evaluating the safety and efficacy of the G-stream drug-eluting stent in the Above-the-Knee femoropopliteal artery (G-streamPAD). Clinicaltrials.gov ID NCT05780359* (2023) [cited 2024 Jan 08]. Available from: https://clinicaltrials.gov/study/NCT05780359
