# Supplementary material for: Introducing and utilizing innovative technologies in health care systems: a country comparison for peripheral drug-eluting stents in Germany and the USA
Source: Front Public Health. 2025 Jun 19;13:1488091. doi: 10.3389/fpubh.2025.1488091 (PMC12222216; doi:10.3389/fpubh.2025.1488091)
Supplement: Supplementary file 1 [file Data_Sheet_1.zip › Supplement_Material/A.16_Financing_instruments.docx]

**A.16 Financing instruments**

| **Germany** | | | **USA (Medicare)** | | |
| --- | --- | --- | --- | --- | --- |
| **Time point / span** | **Financing instrument** | **Source (URL) by year,**  **all last accessed: 01/20/2024** | **Time point / span** | **Financing instrument** | **Source (URL) by year,**  **all last accessed: 02/17/2025** |
| 2009-2011 | NUB payment (innovation payment) | 2009: <https://www.g-drg.de/archiv/drg-systemjahr-2009-datenjahr-2007#sm9>,  2010: <https://www.g-drg.de/archiv/drg-systemjahr-2010-datenjahr-2008#sm9>,  2011: <https://www.g-drg.de/archiv/drg-systemjahr-2011-datenjahr-2009#sm9> | from 2017 | MS-DRG  (flat rate financing) | from 2017: <https://www.cms.gov/medicare/payment/prospective-payment-systems/acute-inpatient-pps/ms-drg-classifications-and-software> |
| from 2012 | G-DRG  (flat rate financing) | from 2012: <https://www.g-drg.de/archiv/drg-systemjahr-2012-datenjahr-2010#sm2> |  |  |  |
| **Legend:** NUB – New Diagnostic and Treatment Methods [Neue Untersuchungs- und Behandlungsmethoden], G-DRG – (German) Diagnosis Related Groups, MS-DRG – Medicare Severity Diagnosis Related Groups | | | | | |
