# Supplementary material for: Introducing and utilizing innovative technologies in health care systems: a country comparison for peripheral drug-eluting stents in Germany and the USA
Source: Front Public Health. 2025 Jun 19;13:1488091. doi: 10.3389/fpubh.2025.1488091 (PMC12222216; doi:10.3389/fpubh.2025.1488091)
Supplement: Supplementary file 1 [file Data_Sheet_1.zip › Supplement_Material/A.18_Synthesis_assumption_of_evidence_and_events_influence.docx]

**A.18 Detailed synthesis: assumption of influence of evidence and further events on case numbers’ development**

The first case numbers for the utilization of drug-eluting stents in the upper leg (DES-UL) were documented for Germany in 2008. At that time, evidence on efficacy and safety for the technology was already available, but to a very limited extent (level of evidence (LoE) Ib: n=1 article, author assessment: "indecisive" (1); LoE IV: n=1 article, author assessment: "positive" (2)). The initial evidence base may have prompted physicians and other decision-makers in hospitals to utilize DES-UL, however, to a moderate extent. In the following year, the case numbers in Germany decreased. Reasons for this could have been that, as before, only a few articles were published and primarily on studies of low LoE (exception: one HTA report from 2008 (3), which cited Duda et al. (2006) (1), author assessment: "indecisive"), insufficient reimbursement for the technology, and individual reasons of physicians and decision-makers in medical institutions (4, 5). In 2009, DES-UL was part of NUB payments in Germany. Hospitals who applied for this financing instrument had the opportunity to negotiate the payment with the health insurance companies. This could be one reason for increasing case numbers. Furthermore, in August 2009, the product Zilver PTX received CE certification and could be utilized by all hospitals in the EU. This may have further encouraged physicians and other clinical decision-makers to opt for the technology.

In the USA, the documentation on the utilization of DES-UL started in 2010 and case numbers increased continuously from then on, while the case numbers in Germany decreased in 2012/2013, before they increased again. One reason for this utilization growth in US hospitals could be that from 2011, the Zilver PTX, an indication-specific stent, has been increasingly investigated in studies. In addition, the first randomized controlled trial (RCT) on DES-UL was published in 2011 by Dake et al. (6), which reported positive results for the Zilver PTX stent, possibly encouraging physicians to adopt it. In the USA, the case numbers had increased sharply in the first year; in the following years (up to 2014), the increase in the case numbers was weaker, possibly also due to individual decisions made by physicians and other clinical decision-makers. The decrease in case numbers in Germany in 2013 could be due to many published safety notices & recalls (n=10) for the Zilver PTX stent. For example, two safety notices, one including a serious adverse event warning, were published on the website^[[1]](#footnote-1)^ of the German Federal Institute for Drugs and Medical Devices [Bundesinstitut für Arzneimittel und Medizinprodukte (BfArM)] regarding incidents in Germany (7, 8). From the following year 2014, the case numbers in Germany increased continuously. Also, the body of evidence for DES-UL steadily increased from this year on, both in terms of quantity of articles on studies with low and high LoE, and size of the study populations. With a few exceptions, the body of evidence contained publications with "positive" and "indecisive" author assessments. The meta-analysis by Katsanos et al. (2014), the first study of highest LoE (Ia), considering a great population size (total: n=2,532 patients; DES-UL: n=572 patients), reported positive results regarding the Zilver PTX stent. Articles on studies with similar characteristics and conclusions followed in the subsequent years. This development in terms of results and the emergence of a solid evidence base could be another reason for the further increase in case numbers in Germany and the USA. However, the case numbers in Germany continued to rise only moderately, maybe due to a clinical guideline for Germany published in 2015 that does not explicitly recommend the utilization of DES-UL, but instead discusses the utilization of other technologies for peripheral arterial disease (9). In contrast, the case numbers in the USA had increased sharply from 2014 onwards, particularly in the period from 2015 to 2016: it can be assumed that the approval of DES-UL products has led to an increased utilization of DES-UL in hospitals. This assumption is supported by the standardized case numbers: in the years following FDA approval of the first stents for the upper leg (UL) – the S.M.A.R.T. and the Zilver PTX stent (both approved in 2012) – case numbers in the USA increased more than in Germany, reaching up to 4.7 times higher in 2016. Furthermore, the greater utilization of DES-UL in the USA might have been due to a high burden of disease in the USA with regard to peripheral vascular diseases caused by obesity and diabetes (10). Also, in 2015, the DES-UL technology was positively evaluated by the Centers for Medicare & Medicaid Services (CMS), so that reimbursement via Medicare Severity Diagnosis Related Groups (MS-DRG/flat rates) from then on was possible. But from 2016, the US case numbers no longer increased quite as sharply. From 2018, the case numbers even decreased for both countries. This could be due to the long-term effects of articles (n=4) producing "negative" author assessments, published between 2014 and 2016. Also, in the period thereafter (until 2019), less articles were published compared to the previous years. Furthermore, a safety notice for the Eluvia stent was published in 11/2017. At that time, the Eluvia stent had not yet been approved in the USA (FDA approval in 09/2018). The case numbers in the USA continued to decrease until the end of the observation period (in 2020), while in Germany, the case numbers increased again between 2019 and 2020. This could be due to an increased number of publications from 2019 onwards, which report positive results from studies with great population sizes, particularly in 2019 (e.g., Freisinger et al. (2019) (11); all authors attributed to German research centers; LoE III; population: n=64,771). In addition, two international clinical guidelines were published in 2019. These report positive study results and recommend the utilization of drug-eluting devices used in the UL (12, 13). However, this apparently had no influence in the USA. Furthermore, the approval of DES-UL products in the EU and the USA may have had an influence on the development of the case numbers, not only in their own country but also in the other country. In Germany, for example, case numbers increased after EU and FDA approval of the Eluvia stent. The development of evidence and the different reported events are shown in **Fig.3** in the article alongside the case number curves for both countries. The standardized case numbers, data sources and formula for their calculation can be found in **Appendix A.4**. A figure presenting the development curves for the standardized case numbers for Germany and the USA can be found in **Appendix A.17**.

**References**

1. Duda SH, Bosiers M, Lammer J, Scheinert D, Zeller T, Oliva V, et al. Drug-eluting and bare nitinol stents for the treatment of atherosclerotic lesions in the superficial femoral artery: long-term results from the SIROCCO trial. *Journal of Endovascular Therapy* (2006) **13**:701–10. doi:10.1583/05-1704.1

2. Feiring AJ, Wesolowski AA. Antegrade popliteal artery approach for the treatment of critical limb ischemia in patients with occluded superficial femoral arteries. *Catheterization and Cardiovascular Interventions* (2007) **69**:665–70. doi:10.1002/ccd.21069

3. Balk E, Cepeda MS, Ip S, Trikalinos T, O'Donnell T. “Horizon scan of invasive interventions for lower extremity peripheral artery disease and systematic review of studies comparing stent placement to other interventions,”. In: *Horizon scan of invasive interventions for lower extremity peripheral artery disease and systematic review of studies comparing stent placement to other interventions*. Rockville (MD): Agency for Healthcare Research and Quality (US) (2008).

4. Felgner S, Ex P, Henschke C. Physicians' decision making on adoption of new technologies and role of coverage with evidence development: a qualitative study. *Value Health* (2018) **21**:1069–76. doi:10.1016/j.jval.2018.03.006

5. Greenberg D, Pliskin JS. Adoption and use of new medical technology at the hospital level. Risk management. *Health Management* (2008) **10**.

6. Dake MD, Ansel GM, Jaff MR, Ohki T, Saxon RR, Smouse HB, et al. Paclitaxel-eluting stents show superiority to balloon angioplasty and bare metal stents in femoropopliteal disease: twelve-month Zilver PTX randomized study results. *Circulation: Cardiovascular Interventions* (2011) **4**:495–504. doi:10.1161/CIRCINTERVENTIONS.111.962324

7. Federal Institute for Drugs and Medical Devices [Bundesinstitut für Arzneimittel und Medizinprodukte (BfArM)]. *2013/02/08. Zilver PTX: safety notice. Germany.* (2013) [cited 2023 Jul 11]. Available from: https://www.bfarm.de/SharedDocs/Kundeninfos/DE/11/2013/08008-12_kundeninfo_de.pdf?__blob=publicationFile&v=4

8. Federal Institute for Drugs and Medical Devices [Bundesinstitut für Arzneimittel und Medizinprodukte (BfArM)]. *2013/05/06. Zilver PTX: safety notice. Germany.* (2013) [cited 2023 Jul 11]. Available from: https://www.bfarm.de/SharedDocs/Kundeninfos/DE/11/2013/02316-13_kundeninfo_de.pdf?__blob=publicationFile

9. Lawall H, Zemmrich C. *S3 guideline on diagnosis, therapy and follow-up of peripheral arterial occlusive disease [S3-Leitlinie zur Diagnostik, Therapie und Nachsorge der peripheren arteriellen Verschlusskrankheit]* (2015). 168 p.

10. Wilcox T, Newman JD, Maldonado TS, Rockman C, Berger JS. Peripheral vascular disease risk in diabetic individuals without coronary heart disease. *Atherosclerosis* (2018) **275**:419–25. doi:10.1016/j.atherosclerosis.2018.04.026

11. Freisinger E, Koeppe J, Gerss J, Goerlich D, Malyar NM, Marschall U, et al. Mortality after use of Paclitaxel-based devices in peripheral arteries: a real-world safety analysis. *European Heart Journal* (2020) **41**:3732–9. doi:10.1093/eurheartj/ehz698

12. Frank U, Nikol S, Belch J. *Guideline on peripheral arterial disease. Volume 48. Supplement 102* (2019). 80 p.

13. Conte MS, Bradbury AW, Kolh P, White JV, Dick F, Fitridge R, et al. Global Vascular Guidelines on the Management of Chronic Limb-Threatening Ischemia. *European journal of vascular and endovascular surgery the official journal of the European Society for Vascular Surgery* (2019) **58**:S1-S109.e33. doi:10.1016/j.ejvs.2019.05.006

1. Federal Institute for Drugs and Medical Devices [Bundesinstitut für Arzneimittel und Medizinprodukte (BfArM)]. Tasks of the BfArM. Differentiation and classification. URL: <https://www.bfarm.de/DE/Medizinprodukte/Aufgaben/Abgrenzung-und-Klassifizierung/_node.html>, last accessed: 07/25/2024. [↑](#footnote-ref-1)
