# Supplementary material for: Introducing and utilizing innovative technologies in health care systems: a country comparison for peripheral drug-eluting stents in Germany and the USA
Source: Front Public Health. 2025 Jun 19;13:1488091. doi: 10.3389/fpubh.2025.1488091 (PMC12222216; doi:10.3389/fpubh.2025.1488091)
Supplement: Supplementary file 1 [file Data_Sheet_1.zip › Supplement_Material/A.2_Registries_guideline_HTA_sources_search_screening.docx]

**A.2 Clinical trial registries, HTA databases, and clinical guideline databases: sources and search/screening terms**

| **Sources for literature and website links (URL)** | **Search and screening terms (used in website searches)** | **Date of latest search** |
| --- | --- | --- |
| **Clinical trial registries** | | |
| International clinical trials registry platform (<https://trialsearch.who.int/AdvSearch.aspx>) | Search terms in sections (1.) "Title" and (2.) "Condition" combined with Boolean operator "AND":   1. Eluvia OR Zilver OR Cypher OR Dynalink OR Xience OR Taxus OR SMART OR "S.M.A.R.T." OR "drug-eluting stent" 2. peripheral OR femor* OR knee OR vascular OR limb OR venous OR arterial OR ischemia | 10/20/2023 |
| ClinicalTrials.gov (<https://clinicaltrials.gov/>) | Search terms in sections (1.) "Condition or disease" and (2.) "Intervention/Treatment" combined without specification of any Boolean operator:   1. peripheral OR femor* OR knee OR vascular OR limb OR venous OR arterial OR ischemia 2. Eluvia OR Zilver OR Cypher OR Dynalink OR Xience OR Taxus OR SMART OR "S.M.A.R.T." OR "drug-eluting stent" | 10/20/2023 |
| **Health technology assessment (HTA) databases** | | |
| HTA Austria: Austrian Institute for Health Technology Assessment (AIHTA) / former Ludwig Boltzmann Institut for Health Technology Assessment (LBI-HTA) (<https://eprints.hta.lbg.ac.at/>) | Search terms:  "drug-eluting stent" OR "medikamentenfreisetzender Stent" [*German* for "drug-eluting stent"] | 10/20/2023 |
| Institute for Quality and Efficiency in Health Care [Institut für Qualität und Wirtschaftlichkeit im Gesundheitswesen (IQWiG)] (<https://www.iqwig.de/projekte/projekte-und-ergebnisse/#searchQuery=query=*&page=1&rows=10&sortBy=score&sortOrder=desc&facet.filter.language=de>) | Search terms:  "medikamentenfreisetzender Stent" [*German* for "drug-eluting stent"] | 10/20/2023 |
| German Agency for Health Technology Assessment [Deutsche Agentur für Health Technology Assessment (DAHTA)], incl. the database of the Federal Institute for Drugs and Medical Devices [Bundesinstitut für Arzneimittel und Medizinprodukte (BfArM)] & German Institute for Medical Documentation and Information [Deutsches Institut für Medizinische Dokumentation und Information (DIMDI)] (<https://www.dimdi.de/dynamic/de/weitere-fachdienste/health-technology-assessment/>) | Search terms:  "medikamentenfreisetzender Stent" [*German* for "drug-eluting stent"] | 10/20/2023 |
| University of York: Centre for Reviews and Dissemination & International Network of Agencies for Health Technology Assessment (CRD/INAHTA) (<https://www.crd.york.ac.uk/CRDWeb/>) | Search terms:  "drug-eluting stent" OR "medikamentenfreisetzender Stent" [*German* for "drug-eluting stent"] | 10/20/2023 |
| European Network for Health Technology Assessment (EUnetHTA) [<https://www.eunethta.eu/rapid-reas/>; Assessment Archive (2006-2015): <https://www.eunethta.eu/assessment-archive-2006-2015/>] | Screening term:  "stent" | 10/20/2023 |
| (German) Federal Joint Committee [Gemeinsamer Bundesausschuss  (G-BA)] (<https://www.g-ba.de/beschluesse>) | Search terms (individually entered in separate searches and in this order):   1. "drug-eluting stent" 2. "medikamentenfreisetzender Stent" [*German* for "drug-eluting stent"] | 10/20/2023 |
| Publicly available reports of the (German) Federal Medical Service [Medizinischer Dienst Bund (MDS)] (<https://md-bund.de/index.html>) | Search term:  "stent" | 10/20/2023 |
| **Clinical guideline databases** | | |
| Association of the Scientific Medical Societies e.V. [Arbeitsgemeinschaft der Wissenschaftlichen Medizinischen Fachgesellschaften e.V. (AWMF)] (<https://www.awmf.org/leitlinien/aktuelle-leitlinien.html> ) | Search terms (individually entered in separate searches and in this order):   1. Drug-eluting stent 2. Medikamentenfreisetzender Stent [*German* for "drug-eluting stent"] 3. Periphere arterielle Verschlusskrankheit [*German* for "Peripheral arterial occlusive disease", *short form:* "PAKV"] 4. PAVK | 10/16/2023 |
| European Society for Vascular Surgery (ESVS) (<http://www.esvs.org/journal/guidelines/>) | Screening terms:   1. Peripheral 2. Vascular 3. Limb 4. Venous | 10/16/2023 |
| European Society for Vascular Medicine (ESVM) (<http://vascular-medicine.org/guidelines/> ) | Screening terms:   1. Peripheral 2. Vascular 3. Limb 4. Venous | 10/16/2023 |
