# Supplementary material for: Introducing and utilizing innovative technologies in health care systems: a country comparison for peripheral drug-eluting stents in Germany and the USA
Source: Front Public Health. 2025 Jun 19;13:1488091. doi: 10.3389/fpubh.2025.1488091 (PMC12222216; doi:10.3389/fpubh.2025.1488091)
Supplement: Supplementary file 1 [file Data_Sheet_1.zip › Supplement_Material/A.3_Procedure_codes.docx]

**A.3 Procedure codes**

| **Procedure codes for drug-eluting stents in the upper leg; used for identification of hospital case numbers, per country** | | |
| --- | --- | --- |
| **Country: Germany** | | |
| Time frame | Procedure codes | Source (URL) by year, all last accessed: 07/04/2023 |
| 2008-2016 | 8-8410b, 8-8411b, 8-8412b, 8-8413b, 8-8414b, 8-8415b | 2008: <https://www.dimdi.de/static/de/klassifikationen/ops/kode-suche/opshtml2008/>,  2009: <https://www.dimdi.de/static/de/klassifikationen/ops/kode-suche/opshtml2009/>,  2010: <https://www.dimdi.de/static/de/klassifikationen/ops/kode-suche/opshtml2010/block-8-80...8-85.htm>,  2011: <https://www.dimdi.de/static/de/klassifikationen/ops/kode-suche/opshtml2011/block-8-80...8-85.htm>,  2012: <https://www.dimdi.de/static/de/klassifikationen/ops/kode-suche/opshtml2012/block-8-80...8-85.htm>,  2013: <https://www.dimdi.de/static/de/klassifikationen/ops/kode-suche/opshtml2013/block-8-80...8-85.htm>,  2014: <https://www.dimdi.de/static/de/klassifikationen/ops/kode-suche/opshtml2014/block-8-80...8-85.htm>,  2015: <https://www.dimdi.de/static/de/klassifikationen/ops/kode-suche/opshtml2015/block-8-80...8-85.htm>,  2016: <https://www.dimdi.de/static/de/klassifikationen/ops/kode-suche/opshtml2016/block-8-80...8-85.htm>, |
| 2017-2020 | 8-8410s, 8-8411s, 8-8412s,  8-8413s, 8-8414s, 8-8415s,  8-8410t, 8-8411t, 8-8412t,  8-8413t, 8-8414t, 8-8415t | 2017: <https://www.dimdi.de/static/de/klassifikationen/ops/kode-suche/opshtml2017/block-8-80...8-85.htm>,  2018: <https://www.dimdi.de/static/de/klassifikationen/ops/kode-suche/opshtml2018/block-8-80...8-85.htm>,  2019: <https://www.dimdi.de/static/de/klassifikationen/ops/kode-suche/opshtml2019/block-8-80...8-85.htm>,  2020: <https://www.dimdi.de/static/de/klassifikationen/ops/kode-suche/opshtml2020/block-8-80...8-85.htm> |
| **Country: USA** | | |
| 2006-2014 | 0060 | 2006-2014: <https://www.findacode.com/icd-9/icd-9-v3-procedure-codes.html> |
| 2015-2020 | X27H385, X27H395, X27H3B5, X27H3C5, X27J385, X27J395, X27J3B5, X27J3C5, X27K385, X27K395, X27K3B5, X27K3C5, X27L385, X27L395, X27L3B5, X27L3C5, X27M385, X27M395, X27M3B5, X27M3C5, X27N385, X27N395, X27N3B5, X27N3C5, 047K34Z, 047K35Z, 047K36Z, 047K37Z, 047L34Z, 047L35Z, 047L36Z, 047L37Z, 047M34Z, 047M35Z, 047M36Z, 047M37Z, 047N34Z, 047N35Z, 047N36Z, 047N37Z | 2015-2020: <https://www.findacode.com/code-set.php?set=ICD10PCS> |
