# Supplementary material for: Introducing and utilizing innovative technologies in health care systems: a country comparison for peripheral drug-eluting stents in Germany and the USA
Source: Front Public Health. 2025 Jun 19;13:1488091. doi: 10.3389/fpubh.2025.1488091 (PMC12222216; doi:10.3389/fpubh.2025.1488091)
Supplement: Supplementary file 1 [file Data_Sheet_1.zip › Supplement_Material/A.4_Standardization_overview_sources_formula.docx]

**A.4 Standardization of case numbers: tabular overview of population numbers per year, data sources, and formula**

**I Tabular overview:**

| **Year** | **Germany** | | | **USA** | | |
| --- | --- | --- | --- | --- | --- | --- |
|  | **Case numbers** | **Population** | **Case numbers: 100,000 inhabitants/ population** | **Case numbers** | **Population** | **Case numbers: 100,000 inhabitants/ population** |
| **2008** | 43 | 82,002,356 | 0,0524 | 0 | 304,093,966 | 0,0000 |
| **2009** | 28 | 81,802,257 | 0,0342 | 0 | 306,771,529 | 0,0000 |
| **2010** | 70 | 81,751,602 | 0,0856 | 138 | 309,327,143 | 0,0446 |
| **2011** | 195 | 80,327,900* | 0,2428 | 1018 | 311,583,481 | 0,3267 |
| **2012** | 249 | 80,523,746 | 0,3092 | 1150 | 313,877,662 | 0,3664 |
| **2013** | 186 | 80,767,463 | 0,2303 | 1635 | 316,059,947 | 0,5173 |
| **2014** | 199 | 81,197,537 | 0,2451 | 2075 | 318,386,329 | 0,6517 |
| **2015** | 254 | 82,175,684 | 0,3091 | 3470 | 320,738,994 | 1,0819 |
| **2016** | 317 | 82,521,653** | 0,3841 | 5705 | 323,071,755 | 1,7659 |
| **2017** | 394 | 82,792,351** | 0,4759 | 6410 | 325,122,128 | 1,9716 |
| **2018** | 489 | 83,019,213 | 0,5890 | 7085 | 326,838,199 | 2,1677 |
| **2019** | 453 | 83,166,711 | 0,5447 | 7055 | 328,329,953 | 2,1488 |
| **2020** | 496 | 83,155,031 | 0,5965 | 6885 | 331,511,512 | 2,0769 |
| **Legend:** * from 2011 results of the population update based on the 2011 German population census; ** the development of the German population for 2016 and 2017 is comparable with the previous year's number to a limited extent, there are limitations in the accuracy of the results in 2016 according to information on the website of the German Federal Statistical Office (source: see below) | | | | | | |

**II Sources (URL):**

1. German Federal Statistical Office. Population status: population by nationality and gender. Population as of December 31 of the respective year. Source: Population update based on last census data 2011 [Statistisches Bundesamt (Destatis). Bevölkerungsstand: Bevölkerung nach Nationalität und Geschlecht. Bevölkerung am 31. Dezember des Jahres. Quelle: Fortschreibung des Bevölkerungsstandes auf Grundlage des letzten Zensus 2011]. URL: <https://www.destatis.de/DE/Themen/Gesellschaft-Umwelt/Bevoelkerung/Bevoelkerungsstand/Tabellen/deutsche-nichtdeutsche-bevoelkerung-nach-geschlecht-deutschland.html#fussnote-2-249820>**,** last accessed: 12/03/2023
2. The World Bank. Databank. Population estimates and projections: United States. Midyear estimates. Source: Total population estimates and projections based on the de facto definition of population (all residents regardless of legal status or citizenship). URL: <https://databank.worldbank.org/source/population-estimates-and-projections/Type/TABLE/preview/on>**,** last accessed: 12/03/2023

**III Formula for standardization calculation:**

$$Standardization rate per 100,000 inhabitants=\left( \frac{{case numbers}_{GER, USA}}{{populations size}_{GER, USA}} \right)\times100,000$$
