# Supplementary material for: Introducing and utilizing innovative technologies in health care systems: a country comparison for peripheral drug-eluting stents in Germany and the USA
Source: Front Public Health. 2025 Jun 19;13:1488091. doi: 10.3389/fpubh.2025.1488091 (PMC12222216; doi:10.3389/fpubh.2025.1488091)
Supplement: Supplementary file 1 [file Data_Sheet_1.zip › Supplement_Material/A.5_Evidence_and_study_results_development_description.docx]

**A.5** **Detailed description of the evidence & study results (author assessment) development**

For the observation period 2006 to 2022, n=100 publications on primary studies were identified: n=10 articles on nine randomized controlled trials (RCTs, level of evidence (LoE) Ib), n=12 articles on prospective comparative cohort studies (LoE IIb), n=29 articles on retrospective comparative studies (LoE III), and n=49 publications on non-comparative studies (LoE IV). In addition, n=17 articles on systematic reviews, meta-analyses, and HTA reports (LoE Ia & IIa) were identified.

Until 2010, only a few articles on studies investigating drug-eluting stents (DES) in upper leg (UL) vessels with small population sizes had been published, e.g., in Feiring et al. (2007) (n=5 patients, LoE IV) (1). From the year 2013 onwards, the number of publications increased and the evidence base consolidated. Most publications were identified for the years 2019 (n=20 articles) and 2020 (n=21 articles). Both, the number of articles on non-comparative studies (including case series), and on studies with high LoE (<IV) have increased over the years. Also, results on studies with high patient numbers were published, e.g., in Dake et al. (2011) (2) (n=787 patients, LoE IV) and in Dake et al. (2013) (3) (n=1,023 patients; LoE III). In particular in the years 2014 to 2016, more publications on studies of high LoE were published, i.e.,
LoE Ia: n=3 articles [e.g., Jens (2014) (4), Katsanos (2014) (5)], LoE Ib: n=2 articles (6, 7), and LoE IIa: n=4 articles [e.g., Marmagkiolis (2014) (8), Falkenberg (2015) (9)]. Articles on primary studies show a focus on the Zilver PTX stent, the first DES approved for utilization in femoral vessels (10, 11). During the observation period, the Zilver PTX stent was evaluated in n=65 articles on primary studies [e.g., (7), (12), (13)]. Results on the Eluvia stent (one of the other DES used in the UL) are reported in n=20 articles [e.g., (14), (15), (16)] [Cypher: n=2 articles (1, 17), S.M.A.R.T.: n=1 article (18), Dynalink-E: n=1 article (19), Taxus Liberté: n=1 article (20), and Xience V: n=1 article (17)]. In n=22 articles on primary studies, no product names of investigated DES-UL were given [e.g., (21), (22), (23)]. The vast majority of the RCTs identified (n=7 out of nine) investigated the Zilver PTX. One RCT is the ZILVER PTX trial reported in articles by Dake et al. (2011, 2013, 2016, and 2019) (3, 6, 11, 24). Other RCTs include REAL PTX (25), DRASTICO (26), ZILVERPASS (27), BATTLE (28), and the trials by Miki et al. (2016) (7) and Falkowski et al. (2020) (29). The SIROCCO trial was the first RCT evaluating a coronary DES (S.M.A.R.T.) for utilization in femoral vessels. As the SIROCCO trial is divided into two phases, a distinction is made between "SIROCCO I" and "SIROCCO II". In SIROCCO I patients were implanted with a maximum of three DES, and in SIROCCO II with a maximum of two DES. An overview of the identified articles of LoE Ib, IIb, III, and IV studies, and extracted data can be found in **Appendix A.6**.

The first publication of a study within this category of highest LoE (Ia & IIa) published within our observation period is the HTA report (LoE IIa) of Balk et al. from 2008 (30), in which results of the SIROCCO I trial (18), and a prospective study for DES in the infrapopliteal vessels [lower leg (LL)] (31) are discussed. Other HTA reports that followed are, for example, the one on DES in femoral vessels that has been published by the institute Health Quality Ontario in 2015 (LoE IIa) (32). The first relevant systematic reviews and meta-analyses of the highest LoE Ia (n=4 articles, without HTA reports) were published in the years 2014 (4, 5), 2015 (33), and 2017 (34). Further publications of systematic reviews and meta-analyses followed in 2014 (n=4 articles), 2015 (n=3 articles), and 2017 (n=3 articles); and at the end of our observation period in 2019 (n=3 articles), 2021 (n=2 articles), and 2022 (n=1 article). For example, in the publication by Nugraha et al. (2022) (35) four studies investigating the ZILVER PTX stent [n=2 RCTs: REAL PTX (25) and DRASTICO (26), and two prospective cohort studies (36, 37)] are addressed. In three publications of LoE Ia & IIa studies DES for transtibial vessels in the LL were considered in addition to DES-UL (9, 10, 30). In these, there was a differentiated reporting of the results regarding the location/indication area of the DES, so that these publications could be included in our analysis. An overview of the identified LoE Ia & IIa studies and extracted data can be found in **Appendix A.7**. **Appendix A.8** contains an overview of publications identified in the HTA database searches. In another six identified publications on LoE Ia & IIa studies, the reported results in the conclusion or results sections of the articles did not clearly differentiate between DES utilization in vessels in the UL or LL. These include five systematic reviews and meta-analyses of RCTs [LoE Ia (38–42)] and one systematic review of observational studies [LoE IIa (43)], which we consequently did not include in the data extraction; their results are presented narratively only.

Of all identified publications, n=41 articles have a "positive" author assessment. For many articles, conclusions and reported results were classified as "indecisive" (n=58 articles). For n=13 articles the author assessment is "neutral" and for five articles it is "negative". Most articles on RCTs (LoE Ib) were assessed as "positive" (n=5 articles) and "indecisive" (n=4 articles); one was considered as "neutral" (n=1 article). Author assessments of LoE IV articles (n=49 articles) were predominantly assessed as "indecisive" (n=24 articles), followed by "positive" (n=18 articles), "negative" (n=5 articles), and "neutral" (n=2 articles) author assessments. "Indecisive" author assessments were found in most articles on LoE IIb (n=6 articles) and LoE Ia & IIa studies (n=11 articles). Most articles on LoE III studies were rated "positive" (n=13 articles). "Negative" author assessments were only concluded for articles on primary studies of LoE IV [n=5 articles (44–48)]. None of the LoE Ia & IIa study articles concluded with a "negative" author assessment. In the first seven years of the observation period (2006 to 2012), there was a low publication density [n=7 articles; of which "indecisive": n=4 (2, 18, 19, 30) and "positive": n=3 (1, 11, 17)]. The articles with "positive" author assessments are on two studies of LoE IV by Feiring et al. (2007) (1) and Karnabatidis et al. (2012) (17) (already published online in 2011), and to the RCT reported by Dake et al. (2011) (11). The latter article was the first one on a LoE Ib trial to date reporting positive study results of
DES-UL regarding safety and vessel openness rates compared to the control intervention after a 1-year follow-up. Publications from 2013 onwards, including articles on studies of all LoE
(Ia-IV) considered represent all variations of author assessments. Many publications conclude with an "indecisive" or a "positive" assessment (n=54 and n=38 articles; "neutral": n=13 articles). "Negative" author assessments (n=5 articles) are given in only a few articles: for n=5 non-comparative studies and case series (LoE IV) (44–48). For example, Tomoi et al. (2015) (45) (n=5 patients) report an in-stent thrombus and assumed a delayed vessel healing and persistent inflammation 12 months after Zilver PTX implantation. In addition, Kang et al. (2016) (47) (n=87 patients) report lower patency rates when using the Zilver PTX stent after one year of implantation compared to clinical trial data. Since 2013, there has been an increase of articles on studies with large population sizes, e.g., n=1,261 patients reported in Dake et al. (2013) (3) (LoE III), and from 2014 onwards on studies of high LoE (Ia, Ib, and IIa) (n = 25 articles) with only "positive" (n=7 articles), "indecisive" (n=14 articles), and "neutral" (n=4 articles) author assessments. For example, the articles on the RCT ZILVER-PTX (6) and by Miki et al. (7), both published in 2016, report positive results for the Zilver PTX stent. In the latter the authors state that the utilization of DES would result in a reduced restenosis rate. A high density of publications continued in 2017 but articles on high LoE studies only produced "indecisive" author assessments. While there were just a few publications on studies (n=2 articles) investigating DES-UL in 2018, many articles were published in the following years, particularly in 2019 (n=20 articles), 2020 (n=21 articles), and 2021 (n=15 articles). In these three years, the articles published were primarily on retrospective non-comparative studies (LoE III, n=22 articles), and on the studies with the highest population numbers in primary studies of the overall analysis (2006-2022). In this context, the articles by Behrendt et al. (2020) (49), Secemsky et al. (2021) (50), and Bertges et al. (2020) (51) (e.g., n=21,546; n=16,796; and n=8,376 patients) need to be mentioned, all concluding with "positive" author assessments. In the period from 2019 to 2022, only one article with a "negative" author assessment was published. In this article, Miki et al. (2020) (48) report on neointimal hyperplasia in superficial femoral artery lesions after implantation of drug-eluting self-expanding stents (SES). Overall, however, the picture at the end of the observation period (since 2019) is clearly characterized by "indecisive" (n=34 articles) and "positive" (n=19 articles) author assessments ("neutral": n=9 articles). However, it should be noted that the literature research was conducted only up to 08/2022.

The six publications that do not allow a separate consideration of DES by indication area – the UL or the LL – conclude "positive" in the middle of the observation period (2013 and 2014, n=3 articles), and with "indecisive" author assessments at its end (2020 and 2021, n=3 articles). One article with a "positive" author assessment is that of Simpson et al. (2013) (38). The authors consider DES, alongside drug-eluting balloons and SES, to be the most promising technology for the treatment of peripheral arterial disease but also address the need for further research activity in this field. Furthermore, Amlani et al. (2021) (40) (author assessment: "indecisive") report incomplete follow-up data in studies and ongoing investigations on DES-UL, the results of which remain to be seen.

**References**

1. Feiring AJ, Wesolowski AA. Antegrade popliteal artery approach for the treatment of critical limb ischemia in patients with occluded superficial femoral arteries. *Catheterization and Cardiovascular Interventions* (2007) **69**:665–70. doi:10.1002/ccd.21069

2. Dake MD, Scheinert D, Tepe G, Tessarek J, Fanelli F, Bosiers M, et al. Nitinol stents with polymer-free Paclitaxel coating for lesions in the superficial femoral and popliteal arteries above the knee: twelve-month safety and effectiveness results from the Zilver PTX single-arm clinical study. *Journal of Endovascular Therapy* (2011) **18**:613–23. doi:10.1583/11-3560.1

3. Dake MD, Ansel GM, Jaff MR, Ohki T, Saxon RR, Smouse HB, et al. Sustained safety and effectiveness of Paclitaxel-eluting stents for femoropopliteal lesions: 2-year follow-up from the Zilver PTX randomized and single-arm clinical studies. *Journal of the American College of Cardiology* (2013) **61**:2417–27. doi:10.1016/j.jacc.2013.03.034

4. Jens S, Conijn AP, Koelemay MJ, Bipat S, Reekers JA. Randomized trials for endovascular treatment of infrainguinal arterial disease: systematic review and meta-analysis (Part 1: above the knee). *European journal of vascular and endovascular surgery the official journal of the European Society for Vascular Surgery* (2014) **47**:524–35. doi:10.1016/j.ejvs.2014.02.011

5. Katsanos K, Spiliopoulos S, Karunanithy N, Krokidis M, Sabharwal T, Taylor P. Bayesian network meta-analysis of nitinol stents, covered stents, drug-eluting stents, and drug-coated balloons in the femoropopliteal artery. *Journal of Vascular Surgery* (2014) **59**:1123. doi:10.1016/j.jvs.2014.01.041

6. Dake MD, Ansel GM, Jaff MR, Ohki T, Saxon RR, Smouse HB, et al. Durable clinical effectiveness with Paclitaxel-eluting stents in the femoropopliteal artery: 5-year results of the Zilver PTX randomized trial. *Circulation* (2016) **133**:1472–83. doi:10.1161/CIRCULATIONAHA.115.016900

7. Miki K, Fujii K, Shibuya M, Fukunaga M, Imanaka T, Tamaru H, et al. Comparing the vascular response in implantation of self-expanding, bare metal nitinol stents or Paclitaxel-eluting nitinol stents in superficial femoral artery lesions: a serial optical frequency domain imaging study. *EuroIntervention* (2016) **12**:1551–8. doi:10.4244/EIJ-D-15-00399

8. Marmagkiolis K, Hakeem A, Choksi N, Al-Hawwas M, Edupuganti MM, Leesar MA, et al. 12-month primary patency rates of contemporary endovascular device therapy for femoro-popliteal occlusive disease in 6,024 patients: beyond balloon angioplasty. *Catheterization and Cardiovascular Interventions* (2014) **84**:555–64. doi:10.1002/ccd.25510

9. Falkenberg M, Carlson P, Nordanstig J, Pettersson J, Smidfelt K, Svanberg T, et al. *Drug eluting balloons and stents for symptomatic peripheral arterial disease*. Gothenburg: The Regional Health Technology Assessment Centre (HTA-centrum) Region Vastra Gotaland (2015).

10. Zechmeister-Koss I, Fischer S. *Drug-eluting stents for peripheral arterial occlusive disease [Medikamentenfreisetzende Stents bei peripherer arterieller Verschlusskrankheit]*. Decision support document 75. Wien (2014).

11. Dake MD, Ansel GM, Jaff MR, Ohki T, Saxon RR, Smouse HB, et al. Paclitaxel-eluting stents show superiority to balloon angioplasty and bare metal stents in femoropopliteal disease: twelve-month Zilver PTX randomized study results. *Circulation: Cardiovascular Interventions* (2011) **4**:495–504. doi:10.1161/CIRCINTERVENTIONS.111.962324

12. Mori S, Hirano K, Yamauchi Y, Hayashi E, Doijiri T, Takamura T, et al. Penetration rate of the placement of a drug-eluting stent for the treatment of superficial femoral artery lesions in Japan. *Heart and Vessels* (2017) **32**:1093–8. doi:10.1007/s00380-017-0982-7

13. Hoyt T, Feldman MD, Okutucu S, Lendel V, Marmagkiolis K, McIntosh V, et al. Assessment of vascular patency and inflammation with intravascular optical coherence tomography in patients with superficial femoral artery disease treated with Zilver PTX stents. *Cardiovascular Revascularization Medicine* (2020) **21**:101–7. doi:10.1016/j.carrev.2019.07.009

14. Saratzis A, Rudarakanchana N, Patel S, Diamantopoulos A, Lea T, Corbo B, et al. Interwoven nitinol stents versus drug eluting stents in the femoro-popliteal segment: a propensity matched analysis. *European journal of vascular and endovascular surgery the official journal of the European Society for Vascular Surgery* (2019) **58**:719–27. doi:10.1016/j.ejvs.2019.06.012

15. Kobayashi T, Hamamoto M, Okazaki T, Hasegawa M, Fujiwara T, Takahashi S. Effectiveness of combined superficial femoral artery endovascular therapy with popliteal-to-distal bypass: a paradigm shift in surgical open bypass for chronic limb-threatening ischemia. *Vascular* (2021) **29**:905–12. doi:10.1177/1708538120981224

16. Kwon LM, Hur S, Jae HJ, Min SK, Min SI, Ahn S, et al. One-year outcomes of two different Paclitaxel-eluting stents (Zilver PTX and Eluvia) for trans-atlantic inter-society consensus document (TASC) C/D obstructive femoropopliteal lesions. *Iranian Journal of Radiology* (2022) **19**:e111918. doi:10.5812/iranjradiol.111918

17. Karnabatidis D, Spiliopoulos S, Pastromas G, Katsanos K, Siablis D. Endovascular management of the arteria profunda femoralis: long-term angiographic and clinical outcomes. *CardioVascular and Interventional Radiology* (2012) **35**:1016–22. doi:10.1007/s00270-011-0284-x

18. Duda SH, Bosiers M, Lammer J, Scheinert D, Zeller T, Oliva V, et al. Drug-eluting and bare nitinol stents for the treatment of atherosclerotic lesions in the superficial femoral artery: long-term results from the SIROCCO trial. *Journal of Endovascular Therapy* (2006) **13**:701–10. doi:10.1583/05-1704.1

19. Lammer J, Bosiers M, Zeller T, Schillinger M, Boone E, Zaugg MJ, et al. First clinical trial of nitinol self-expanding everolimus-eluting stent implantation for peripheral arterial occlusive disease. *Journal of Vascular Surgery* (2011) **54**:394–401. doi:10.1016/j.jvs.2011.01.047

20. Gahide G, Phaneuf SC, Cossette M, Banine A, Budimir M, Maghsoudloo K, et al. Paclitaxel and mortality in patients with claudication and de novo femoropopliteal lesions: a historical cohort study. *CVIR Endovascular* (2021) **4**:65. doi:10.1186/s42155-021-00255-1

21. Giordano A, Ferraro P, Corcione N, Messina S, Maresca G, Coscioni E, et al. Comparison of Biolimus versus Everolimus for drug-eluting stents in the percutaneous treatment of infra-inguinal arterial disease. *Current Vascular Pharmacology* (2017) **15**:257–64. doi:10.2174/1570161115666170123094523

22. Secemsky EA, Kundi H, Weinberg I, Jaff MR, Krawisz A, Parikh SA, et al. Association of survival with femoropopliteal artery revascularization with drug-coated devices. *JAMA Cardiology* (2019) **4**:332–40. doi:10.1001/jamacardio.2019.0325

23. Suzuki K, Ueshima D, Higashitani M, Yamauchi Y, Hozawa K, Hayakawa N, et al. Two-year results of endovascular therapy for femoropopliteal artery disease in Japan during the introduction of drug-eluting devices. *Cardiovascular Intervention and Therapeutics* (2022). doi:10.1007/s12928-022-00873-z

24. Dake MD, Ansel GM, Bosiers M, Holden A, Iida O, Jaff MR, et al. Paclitaxel-coated Zilver PTX drug-eluting stent treatment does not result in increased long-term all-cause mortality compared to uncoated devices. *CardioVascular and Interventional Radiology* (2020) **43**:8–19. doi:10.1007/s00270-019-02324-4

25. Bausback Y, Wittig T, Schmidt A, Zeller T, Bosiers M, Peeters P, et al. Drug-eluting stent versus drug-coated balloon revascularization in patients with femoropopliteal arterial disease. *Journal of the American College of Cardiology* (2019) **73**:667–79. doi:10.1016/j.jacc.2018.11.039

26. Liistro F, Angioli P, Porto I, Ducci K, Falsini G, Ventoruzzo G, et al. Drug-eluting balloon versus drug-eluting stent for complex femoropopliteal arterial lesions: the DRASTICO study. *Journal of the American College of Cardiology* (2019) **74**:205–15. doi:10.1016/j.jacc.2019.04.057

27. Bosiers M, Setacci C, Donato G de, Torsello G, Silveira PG, Deloose K, et al. ZILVERPASS study: Zilver PTX stent vs bypass surgery in femoropopliteal lesions. *Journal of Endovascular Therapy* (2020) **27**:287–95. doi:10.1177/1526602820902014

28. Gouëffic Y, Sauguet A, Desgranges P, Feugier P, Rosset E, Ducasse E, et al. A polymer-free Paclitaxel-eluting stent versus a bare-metal stent for de novo femoropopliteal lesions: the BATTLE trial. *JACC. Cardiovascular interventions* (2020) **13**:447–57. doi:10.1016/j.jcin.2019.12.028

29. Falkowski A, Bogacki H, Szemitko M. Assessment of mortality and factors affecting outcome of use of Paclitaxel-coated stents and bare metal stents in femoropopliteal pad. *Journal of clinical medicine* (2020) **9**:1–11.

30. Balk E, Cepeda MS, Ip S, Trikalinos T, O'Donnell T. “Horizon scan of invasive interventions for lower extremity peripheral artery disease and systematic review of studies comparing stent placement to other interventions,”. In: *Horizon scan of invasive interventions for lower extremity peripheral artery disease and systematic review of studies comparing stent placement to other interventions*. Rockville (MD): Agency for Healthcare Research and Quality (US) (2008).

31. Siablis D, Kraniotis P, Karnabatidis D, Kagadis GC, Katsanos K, Tsolakis J. Sirolimus-eluting versus bare stents for bailout after suboptimal infrapopliteal angioplasty for critical limb ischemia: 6-month angiographic results from a nonrandomized prospective single-center study. *Journal of Endovascular Therapy* (2005) **12**:685–95. doi:10.1583/05-1620MR.1

32. Health Quality Ontario. Paclitaxel drug-eluting stents in peripheral arterial disease: a health technology assessment. *Ontario health technology assessment series* (2015) **15**:1–62.

33. Baerlocher MO, Kennedy SA, Rajebi MR, Baerlocher FJ, Misra S, Liu D, et al. Meta-analysis of drug-eluting balloon angioplasty and drug-eluting stent placement for infrainguinal peripheral arterial disease. *Journal of Vascular and Interventional Radiology* (2015) **26**:459. doi:10.1016/j.jvir.2014.12.013

34. Antonopoulos CN, Mylonas SN, Moulakakis KG, Sergentanis TN, Sfyroeras GS, Lazaris AM, et al. A network meta-analysis of randomized controlled trials comparing treatment modalities for de novo superficial femoral artery occlusive lesions. *Journal of Vascular Surgery* (2017) **65**:234. doi:10.1016/j.jvs.2016.08.095

35. Nugraha HG, Hilman S, Santiana L, Dewi DK, Raffaelo WM, Wibowo A, et al. Drug-coated balloon versus drug-eluting stent in patients with femoropopliteal artery disease: a systematic review and meta-analysis. *Vascular and Endovascular Surgery* (2022) **56**:385–92. doi:10.1177/15385744211051491

36. Zeller T, Rastan A, Macharzina R, Tepe G, Kaspar M, Chavarria J, et al. Drug-coated balloons vs. drug-eluting stents for treatment of long femoropopliteal lesions. *J Endovasc Ther* (2014) **21**:359–68. doi:10.1583/13-4630MR.1

37. Lee YJ, Kook H, Ko YG, Yu CW, Joo HJ, Ahn CM, et al. Drug eluting stent vs. drug coated balloon for native femoropopliteal artery disease: a two centre experience. *European Journal of Vascular and Endovascular Surgery* (2021) **61**:287–95. doi:10.1016/j.ejvs.2020.10.008

38. Simpson EL, Michaels JA, Thomas SM, Cantrell AJ. Systematic review and meta-analysis of additional technologies to enhance angioplasty for infrainguinal peripheral arterial occlusive disease. *British Journal of Surgery* (2013) **100**:1128–37. doi:10.1002/bjs.9196

39. Canaud L, Ozdemir BA, Belli A-M, Loftus IM, Thompson MM, Hinchliffe RJ. Infrainguinal angioplasty with drug-eluting stents and balloons. *Journal of Vascular Surgery* (2014) **59**:1721–36. doi:10.1016/j.jvs.2014.03.235

40. Amlani V, Falkenberg M, Nordanstig J. The current status of drug-coated devices in lower extremity peripheral artery disease interventions. *Progress in Cardiovascular Diseases* (2021) **65**:23–8. doi:10.1016/j.pcad.2021.02.002

41. Kuno T, Ueyama H, Mikami T, Takagi H, Numasawa Y, Anzai H, et al. Mortality in patients undergoing revascularization with Paclitaxel eluting devices for infrainguinal peripheral artery disease: insights from a network meta-analysis of randomized trials. *Catheterization and Cardiovascular Interventions* (2020) **96**:E467-E478. doi:10.1002/ccd.29125

42. Qureshi MI, Li HL, Ambler GK, Wong KH, Dawson S, Chaplin K, et al. Antiplatelet and anticoagulant use in randomised trials of patients undergoing endovascular intervention for peripheral arterial disease: systematic review and narrative synthesis. *European Journal of Vascular and Endovascular Surgery* (2020) **60**:77–87. doi:10.1016/j.ejvs.2020.03.010

43. Pietzsch JB, Geisler BP, Garner AM, Zeller T, Jaff MR. Economic analysis of endovascular interventions for femoropopliteal arterial disease: A systematic review and budget impact model for the United States and Germany. *Catheterization and Cardiovascular Interventions* (2014) **84**:546–54. doi:10.1002/ccd.25536

44. Fujihara M, Utsunomiya M, Higashimori A, Yokoi Y, Nakamura M. Outcomes of Zilver PTX stent implantation for the treatment of complex femoropopliteal artery disease. *Heart and Vessels* (2016) **31**:152–7. doi:10.1007/s00380-014-0596-2

45. Tomoi Y, Kuramitsu S, Soga Y, Aihara H, Ando K, Nobuyoshi M. Vascular response after Zilver PTX stent implantation for superficial femoral artery lesions: serial optical coherence tomography findings at 6 and 12 months. *Journal of Endovascular Therapy* (2015) **22**:41–7. doi:10.1177/1526602814566577

46. Iida O, Takahara M, Soga Y, Hirano K, Yamauchi Y, Zen K, et al. The characteristics of in-stent restenosis after drug-eluting stent implantation in femoropopliteal lesions and 1-year prognosis after repeat Eendovascular therapy for these lesions. *JACC: Cardiovascular Interventions* (2016) **9**:828–34. doi:10.1016/j.jcin.2016.01.007

47. Kang WY, Campia U, Didier RJ, Kiramijyan S, Koifman E, Negi SI, et al. A single center experience of Zilver PTX for femoro-popliteal lesions. *Cardiovascular Revascularization Medicine* (2016) **17**:399–403. doi:10.1016/j.carrev.2016.02.004

48. Miki K, Tanaka T, Yanaka K, Yoshihara N, Kimura T, Imanaka T, et al. Influence of self-expanding Paclitaxel-eluting stent sizing on neointimal hyperplasia in superficial femoral artery lesions. *Circulation Journal* (2020). doi:10.1253/circj.CJ-20-0470

49. Behrendt CA, Sedrakyan A, Peters F, Kreutzburg T, Schermerhorn M, Bertges DJ, et al. Long term survival after femoropopliteal artery revascularisation with Paclitaxel coated devices: a propensity score matched cohort analysis. *European Journal of Vascular and Endovascular Surgery* (2020) **59**:587–96. doi:10.1016/j.ejvs.2019.12.034

50. Secemsky EA, Barrette E, Bockstedt L, Bonaca MP, Hess CN, Hanson T, et al. Long-term safety of drug-coated devices for peripheral revascularisation. *EuroIntervention* (2021) **17**:590–8. doi:10.4244/EIJ-D-20-01018

51. Bertges DJ, Sedrakyan A, Sun T, Eslami MH, Schermerhorn M, Goodney PP, et al. Mortality after Paclitaxel coated balloon angioplasty and stenting of superficial femoral and popliteal artery in the vascular quality initiative. *Circulation: Cardiovascular Interventions* (2020):e008528. doi:10.1161/CIRCINTERVENTIONS.119.008528
