# Supplementary material for: Introducing and utilizing innovative technologies in health care systems: a country comparison for peripheral drug-eluting stents in Germany and the USA
Source: Front Public Health. 2025 Jun 19;13:1488091. doi: 10.3389/fpubh.2025.1488091 (PMC12222216; doi:10.3389/fpubh.2025.1488091)
Supplement: Supplementary file 1 [file Data_Sheet_1.zip › Supplement_Material/A.6_Extraction_table_LoE_Ib_IIb_III_and_IV_studies.docx]

**A.6 Extraction table: LoE Ib, IIb, III, and IV studies**

| **No.** | **Author (year)** | **Publ. date** (online, if earliest) | **Study acronym** | **Evaluated technologies (DES product name)** | **Number of patients (total / DES patients^1^ / intervention vs. control group^1,2^ /**  **in analysis^3^ /**  **drop-outs^3^)** | **Median follow-up** | **Endpoints** | **Reported endpoints** | | | | | | **Author assessment** |
| --- | --- | --- | --- | --- | --- | --- | --- | --- | --- | --- | --- | --- | --- | --- |
|  |  |  |  |  |  |  |  | **M** | **AE** | **CM** | **QoL** | **SURE** | **Other** |  |
| **Studies of LoE Ib** | | | | | | | | | | | | | | |
| Ib.1 | Duda et al. (2006) (1) | 2006 | SIROCCO I&II | DES (S.M.A.R.T.) vs. BMS | 93 / 47 / 47 vs. 46 / ns / ns | 24 months | in-lesion segment, restenosis, hemodynamic failure of the stented lesion, ABI, clinical patency, technical success, incidence of serious adverse events | x | x | x |  |  | x | indecisive |
| Ib.2 | Dake et al. (2011) (2) | 2011 | ZILVER PTX | DES (Zilver PTX) vs. PTA, DES vs. BMS | 474 / 241 / 241 vs. 238 / 222 /  19 vs. 17 | 12 months | 12-month rates of event-free survival, primary patency, patency in the primary DES and PTA groups, clinical benefit, primary patency, stent fracture rate, stent integrity | x | x | x |  | x | x | positive |
| Ib.3 | Dake et al. (2016) (3) | 2016 | ZILVER PTX | DES (Zilver PTX) vs. PTA, DES vs. BMS | 474 / 236 / 236 vs. 238 / 127 / 109 vs. 79 | 60 months | 12-month rates of event-free survival, primary patency, patency in the primary DES and PTA groups, clinical benefit, primary patency, stent fracture rate, stent integrity | x | x | x |  | x | x | positive |
| Ib.4 | Miki et al. (2016) (4) | 2016 | none | DES (Zilver PTX) vs. BMS | 26 / 14 / 14 vs. 12 / ns / ns | ns | late lumen area loss at six-month follow-up |  |  |  |  | x |  | positive |
| Ib.5 | Bausback et al. (2019) (5) | 2019 | REAL PTX | DES (Zilver PTX) vs. DCB (and bailout stenting) | 150 / 75 / 75 vs. 75 / 75 / 0 | 6, 12, 24, 36 months | primary patency, major adverse events (death, major amputations, clinically driven target lesion revascularization), clinical outcomes | x | x | x |  | x |  | indecisive |
| Ib.6 | Dake et al. (2020) (6) | 2019 | ZILVER PTX | DES (Zilver PTX) vs. BMS | 479 / 305* / 242 vs. 237 / 479 / ns | 5 years | all-cause mortality | x |  |  |  |  |  | positive |
| Ib.7 | Liistro et al. (2019) (7) | 2019 | DRASTICO | DES (Zilver PTX) vs. DCB | 192 / 96 / 96 vs. 96 | 12 months | 12-month target lesion binary restenosis, TLR, freedom from major amputation | x | x | x |  | x | x | indecisive |
| Ib.8 | Bosiers et al. (2020) (8) | 2020 | ZILVER-PASS | DES (Zilver PTX) vs. bypass graft by surgery | 205 / 105 / 100 vs. 105 / 205 / 15 | 1, 6, 12, 24, 36, 60 months | primary patency, device malfunction or serious device-related or other adverse events, technical success, 30-day complications, primary assisted and secondary patency rates, TLR rate, clinical success | x | x | x |  | x | x | positive |
| Ib.9 | Falkowski et al. (2020) (9) | 2020 | none | DES (Zilver PTX) vs. BMS | 256 / 126 / 126 vs. 130 / ns / ns | 36 months | clinical outcome, restenosis frequency, safety (complications, mortality) | x | x | x |  | x |  | indecisive |
| Ib.10 | Goueffic et al. (2020) (10) | 2020 | BATTLE | DES (Zilver PTX) vs. BMS | 181 / 90 / 90 vs. 91 / 181 / 14 | 2 years | freedom of ISR, technical success, all-cause mortality, major amputation, primary sustained clinical improvement, secondary sustained clinical improvement, primary patency, limb salvage, ABI, TER, TLR, stent fracture, QoL, economic analysis | x | x | x | x | x | x | neutral |
| **Studies of LoE IIb** | | | | | | | | | | | | | | |
| IIb.1 | Ishihara et al. (2013) (11) | 2013 | none | DES (Zilver PTX) vs. BMS | 19 / 10 / 10 vs. 9 | ns | angioscopic findings |  |  |  |  | x |  | neutral |
| IIb.2 | Ishihara et al. (2014) (12) | 2014 | none | DES vs. BMS | 27 / 18 / 18 vs. 9 | ns | angioscopic findings |  |  |  |  | x |  | positive |
| IIb.3 | Kozuki et al. (2016) (13) | 2016 | none | DES (Zilver PTX) vs. BMS | 25 / 14 / 14 vs. 18 | ns | neointimal proliferation, arterial healing |  |  | x |  | x |  | positive |
| IIb.4 | Murata et al. (2016) (14) | 2016 | none | DES (Zilver PTX) | 116 / 112 / 112 vs. 116 | ns | incidence of recurrent restenosis, MALE |  | x | x |  |  |  | indecisive |
| IIb.5 | Mori et al. (2017) (15) | 2017 | none | DES (Zilver PTX) | 314 / 27 / 27 vs. 279 | ns | measurement of penetration rate of DES | x | x | x |  |  |  | indecisive |
| IIb.6 | Björkman et al. (2018) (16) | 2018 | none | DES (Zilver PTX) vs. BSX | 41 / 23 / 23 vs. 18 / 23 / 5 | 24 months | overall patency, primary patency, primary assisted patency, change in ankle-brachial index, amputation-free survival | x | x |  |  | x |  | indecisive |
| IIb.7 | Saratzis et al. (2019) (17) | 2019 | none | DES (Zilver PTX, Eluvia) vs. BMS | 296 / 160 / 160 vs. 136 | 2 years | occlusion, restenosis | x | x | x |  | x |  | neutral |
| IIb.8 | Aihara et al. (2020) (18) | 2020 | none | DES (Zilver PTX) vs. BMS vs. stent graft vs. PTA | 220 / 20 / 20 vs. 36 vs. 204 | 1 year (mean 358±166 days) | angiographic success |  |  |  |  | x |  | indecisive |
| IIb.9 | Iida et al. (2020) (19) | 2020 | none | DES (Zilver PTX, Eluvia) vs. BMS vs. plain balloon vs. stent graft | 1578 / 221 / 221 vs. 566 vs. 929 vs. 83 | 1 year ±2 months | patency |  |  |  |  | x |  | indecisive |
| IIb.10 | Kobayashi et al. (2020) (20) | 2020 | none | DES (Eluvia) vs. PTA vs. SES vs. DCB vs. covered stent vs. woven nitinol stent | 49 / 15 limbs / 15 vs. 4 vs. 18 vs. 4 vs. 10 vs. 1 (limbs) | 1, 2, 3, 4, 6 months, +every  3 months thereafter (mean 17 +-17 months) | primary and secondary patency of graft, primary patency, CD-TLR, limb salvage, survival, wound healing | x | x | x |  | x | x | indecisive |
| IIb.11 | Lee et al. (2020) (21) | 2020 | none | DES (Zilver PTX) vs. DCB | 242 / 93 / 93 vs. 149 | median 19.6 months | primary clinical patency, TLR, MALE | x | x |  |  | x |  | indecisive |
| IIb.12 | Yoshioka et al. (2021) (22) | 2021 | none | DES (Eluvia) vs. DCB | 151 / 65 / 65 vs. 86 | 1 year | primary patency,  CD-TLR |  |  |  |  | x |  | neutral |
| **Studies of LoE III** | | | | | | | | | | | | | | |
| III.1 | Dake et al. (2013) (23) | 2013 | none | DES (Zilver PTX) vs. PTA vs. BMS | 474 (RCT), 787 (single-arm study) / 1,023 / 1,023 vs. 238 | 24 months | lesions, patency | x |  |  |  |  |  | positive |
| III.2 | Miki et al. (2014) (24) | 2014 | none | DES vs. BMS | 32 / 11 / 11 vs. 21 | ns | stent volume, neointimal volume, and late lumen loss at 6-month follow-up |  |  |  |  | x |  | positive |
| III.3 | Kay et al. (2015) (25) | 2015 | none | DES (Zilver PTX) vs. BMS vs. other | 205 / 10 / 10 vs. 195 | 11 months | patency rates | x | x | x |  | x |  | positive |
| III.4 | Ansel et al. (2017) (26) | 2017 | none | DES (Zilver PTX) vs. BMS | 33 / 19 / 19 vs. 14 | 12 months | Rutherford Index, ABI, TLR |  |  | x |  |  | x | positive |
| III.5 | Giordano et al. (2017) (27) | 2017 | none | DES | 80 / 80 / ns | 12 months | death, amputation, revascularization, their composite, change in Fontaine class, bypass | x | x | x |  |  |  | indecisive |
| III.6 | Freisinger et al. (2019) (28) | 2019 | none | DES vs. DCB vs. BMS vs. POBA | 64,771 / 676 / 676 vs. 2,648 vs. 28,290 vs. 33,157 | median  92 months | long-term mortality | x |  |  |  |  |  | positive |
| III.7 | Haine et al. (2019) (29) | 2019 | none | DES vs. DCB vs. BMS vs. PTA | 234 / 110 / 110 vs. 124 | 12 months | CD-TLR, time to death, amputation/composite of death, amputation/CD-TLR | x |  | x |  | x |  | indecisive |
| III.8 | Katsuki et al. (2019) (30) | 2019 | none | DES (Zilver PTX) vs. BMS vs. angioplasty | 1,535 / 285 / 285 vs. 1,250 | median  3.4 years (IQR 2.1, 5.7) | mortality | x |  |  |  |  |  | indecisive |
| III.9 | Kibrik et al. (2019) (31) | 2019 | none | DES (Zilver PTX) vs. BMS | 170 / 46 / 46 vs. 124 | mean 7.18+-7.96 months | stent thrombosis, restenosis, re-inter-vention, limb loss |  |  | x |  | x |  | neutral |
| III.10 | Kumins et al. (2019) (32) | 2019 | none | DES (Zilver PTX) vs. DCB vs. PTA vs. stenting and atherectomy | 1,170 / 159* / 159 vs. 295 vs. 311 vs. 176 vs. 33 vs. 182 vs. 14 | mean 27 months (range,  0.1-102 months) | mortality | x |  |  |  |  |  | indecisive |
| III.11 | Phair et al. (2019) (33) | 2019 | none | DES (Zilver PTX) vs. DCB vs. DES and DCB | 97 / 63* / 63 vs. 21 vs. ns | median  13 months (IQR 7–16) | primary patency |  |  |  |  | x |  | indecisive |
| III.12 | Secemsky et al. (2019) (34) | 2019 | none | DES vs.  DES and DCB vs. DCB vs. PTA vs. BMS vs. adjunctive atherectomy | 16,560 / 2,553* / 2,553 vs. 2,709 vs. 5,796 vs. 727 vs. 4,775 vs. 3,503 | median  389 days (IQR 277-508 days) | all-cause mortality | x |  |  |  |  |  | positive |
| III.13 | Behrendt et al. (2020) (35) | 2020 | none | DES vs. PTA vs. BMS vs. DCB | 21,546 / 3902 / 1,283 vs. 2,619 vs. 2,161 vs. 1,473 vs. 3,764 vs. 3,375 vs. 2,351 vs. 4,520 | median 983 days (IQR 412-1777 days [4.9 years])^4^ | overall survival, amputation free survival, major cardiovascular events | x | x |  |  |  |  | positive |
| III.14 | Bertges et al. (2020) (36) | 2020 | none | DES vs. PTA vs. DCB vs. BMS | 8,376 / 684 / 684 vs. 2,104 vs. 3,543 vs. 2,045 | median 16.8 months (IQR 10.1-22.1 months), mean 15.3 months (SD 8.7 months) | mortality | x |  |  |  |  |  | neutral |
| III.15 | Cho et al. (2020) (37) | 2020 | none | DES (Zilver PTX) vs. BMS vs. SES | 27 / 1 limb / 1 vs. 1 vs. 12 vs. 15 (limbs) | 1 year | technical success rate, TLR, patency rates, any complications |  |  |  |  | x | x | indecisive |
| III.16 | Draxler et al. (2020) (38) | 2020 | none | DES vs. BMS vs. stent grafts | 247 / 2 / 2 vs. 234 vs. 11 | 12, 36, 56 months | stent patency, MALE, limb salvage, mortality |  | x | x |  | x |  | indecisive |
| III.17 | Mathlouthi et al. (2020) (39) | 2020 | none | DES (Zilver PTX) vs. BMS | 296 / 101 / 101 vs. 195 | median  23 months (IQR 7-40) | all-cause mortality,  limb salvage, primary patency, primary-assisted patency, secondary patency | x |  | x |  | x |  | indecisive |
| III.18 | Rocha-Singh et al. (2020) (40) | 2020 | none | DES (Zilver PTX) vs. BMS vs. DCB | 2,185 / 1,382 / 1,382 vs. 803 | median  4 years | all-cause mortality | x |  |  |  |  |  | indecisive |
| III.19 | Saratzis et al. (2020) (41) | 2020 | none | DES (Zilver PTX, Eluvia) vs. DCB | 885* / 335 / 335 vs. 550 | median  24 months | major amputation, MACE, death | x | x | x |  | x |  | positive |
| III.20 | Weissler et al. (2020) (42) | 2020 | none | DES vs. DCB vs. PTA vs. BMS vs. other | 1,493 / 105 procedures / 105 vs. 783 vs. 468 vs. 241 vs. 69 | 6, 12, 24 months | all-cause mortality, second surgical, endovascular revascularization, major amputation, minor amputation, all-cause readmission | x |  | x |  |  |  | neutral |
| III.21 | Altaf et al. (2021) (43) | 2021 | none | DES (Eluvia, Zilver PTX) | 465 / 465 / ns | 24 months | CD-TLR, percutaneous intervention, surgery, major amputation, hospitalization outcomes (hospital days for all AE) |  | x |  |  | x | x | positive |
| III.22 | Barenbrock et al. (2021) (44) | 2021 | none | DES vs. BMS vs. DCB vs. PTA | 602 / 47 (unclear) / 47 vs. 226 vs. 377 vs. 572 (unclear) | 6, 12 months | technical success rate, complications, all-cause mortality, MALE  (re-EVR, bypass, or amputation of the lower limb), MCE (acute coronary syndrome, percutaneous coronary intervention, coronary artery bypass graft, stroke), patients’ vital status | x | x | x |  |  | x | indecisive |
| III.23 | Choi et al. (2021) (45) | 2021 | none | DES vs. PTA vs. BMS vs. DCB | 1,724 / 101 / 101 vs. 476 vs. 865 vs. 282 | median 552 days (inter-quartile range, 404-688 days) | mortality | x |  |  |  |  |  | neutral |
| III.24 | Gahide et al. (2021) (46) | 2021 | none | DES (Zilver PTX, Eluvia, Taxus) vs. PTA vs. DCB | 664 / 39 / 39 vs. ns | 1, 2, 5 years^4^ | mortality | x |  |  |  |  |  | indecisive |
| III.25 | Kumins et al. (2021) (47) | 2021 | none | DES (Zilver PTX) vs. BMS vs. PTA vs.  PTA and atherectomy vs. adjunctive atherectomy | 983 / 146* / 146 vs. 242 vs. 300 vs. 133 vs. 24 vs. 128 vs. 10 | 4 years (mean 2.28 +- 1.83 years) | overall survival, amputation-free survival, ff-MA, ff-TVR | x | x | x |  | x |  | positive |
| III.26 | Nakamura et al. (2021) (48) | 2021 | none | DES (Zilver PTX, Eluvia) vs. DCB vs. BMS vs. balloon | 2,581 / 1,001 / 1,001 vs. 388 vs. 1,192 vs. 991 vs. 201 | median 3.0 years | all-cause death, 5-year cumulative mortality, major adverse events, deaths, target limb amputations | x | x | x |  | x |  | indecisive |
| III.27 | Secemsky et al. (2021) (49) | 2021 | none | DES vs. DCB vs. PTA/BMS | 16,796 / 827 / 827 vs. 3,600 vs. 12,369 | median 2.66 years (IQR 2.02–3.52) | all-cause mortality | x |  |  |  |  | x | positive |
| III.28 | Miura et al. (2022) (50) | 2022 | ZERO/DCBATE SFA | DES (Zilver PTX) vs. DES and medication vs. BMS vs. BMS and medication | 337 / 85 / 85 vs. 85 vs. 85 vs. 82 | 1, 3, 6, 12 months | 12-month patency rate | x | x |  |  | x |  | positive |
| III.29 | Suzuki et al. (2022) (51) | 2022 | GUIDE SFA | DES vs. PTA vs. BMS vs. covered stent vs. DCB | 999 / 55 / 55 vs. 387 vs. 348 vs. 120 vs. 89 | 1 year | freedom from clinically driven TVR, maximum walking distance, severe adverse event within 30-days (death, myocardial infarction, stroke, major amputation, ALI, surgical re-intervention) | x | x | x |  | x |  | positive |
| **Studies of LoE IV** | | | | | | | | | | | | | | |
| IV.1 | Feiring et al. (2007) (52) | 2007 | none | DES (Cypher) | 5 / 5 | 29 months | angiographic restenosis, death, amputation, target lesion revascularization | x | x | x |  | x |  | positive |
| IV.2 | Dake et al. (2011) (53) | 2011 | none | DES (Zilver PTX) | 787 / 787 | 12 months | event free survival, patency rate, freedom from target lesion revascularization | x | x | x |  |  | x | indecisive |
| IV.3 | Karnabatidis et al. (2011) (54) | 2011 | none | DES (Cypher, Xience V) vs. PTA | 20 / 12 lesions / 12 vs. 11 lesions | ns | technical success, angiographic lesion primary patency, angiographic binary  in-lesion restenosis, TLR rates |  | x |  |  |  | x | positive |
| IV.4 | Lammer et al. (2011) (55) | 2011 | STRIDES | Dynalink-E | 104 / 104 | ns | rate of in-stent binary restenosis |  |  | x |  | x |  | indecisive |
| IV.5 | Bosiers et al. (2013) (56) | 2013 | Zilver PTX | DES (Zilver PTX) | 135 lesions / 134 | 12 months | device-related death, clinically driven target lesion revascularization, target limb ischemia requiring surgical intervention (bypass or amputation), surgical repair of the target vessel | x | x | x |  | x |  | positive |
| IV.6 | Fanelli et al. (2013) (57) | 2013 | none | DES (Zilver PTX) | 787 / 787 | 12 months | mean lesion length or lesion calcification, procedural success, patency | x | x | x |  |  | x | positive |
| IV.7 | Fujihara et al. (2014) (58) | 2014 | none | DES (Zilver PTX) | 60 / 60 | 13.2 months | primary patency, technical success rate, freedom from TLR rate, AFS rate, Rutherford, ABI, amputation, death | x | x | x |  | x | x | negative |
| IV.8 | Leopardi et al. (2014) (59) | 2014 | none | DES (Zilver PTX) | 69 / 69 | ns | limb salvage, primary patency, intraoperative success, secondary patency rates, stent integrity, post-treatment clinical benefits | x |  | x |  |  | x | neutral |
| IV.9 | Ohki et al. (2014) (60) | 2014 | Zilver PTX | DES | 474 / 236 / 236 vs. 238 | 24 months | event-free survival, freedom from TLR, patency, stent fracture, sustained clinical benefit | x | x | x |  | x | x | positive |
| IV.10 | Iida et al. (2015) (61) | 2015 | ZEPHYR | DES (Zilver PTX) | 690 / 690 | up to 12 | 1-year restenosis rate, MALE, stent thrombosis |  | x | x |  | x |  | indecisive |
| IV.11 | Tomoi et al. (2015) (62) | 2015 | none | DES (Zilver PTX) | 5 / 5 | ns | neointimal thickness and apposition on each strut, incidence of ESL, PLIA, neovascularization at  1-mm intervals |  |  | x |  |  |  | negative |
| IV.12 | Banerjee et al. (2016) (63) | 2016 | XLPAD | DES (Zilver PTX) vs. BMS vs. stent grafts vs. mimetic stents | 604 / 604 | 6 months | MALE, major adverse cardiovascular or cerebrovascular events | x | x | x |  |  |  | indecisive |
| IV.13 | Iida et al. (2016) (64) | 2016 | ZEPHYR | DES | 495 / 495 | ns | re-re-EVT (undergoing additional EVT more than once during the  2-year follow-up after DES implantation),  re-EVT (undergoing at least one additional EVT during the follow-up) |  |  | x |  |  |  | indecisive |
| IV.14 | Iida et al. (2016) (65) | 2016 | ZEPHYR | DES | 210 / 210 | ns | characteristics of ISR after drug-eluting stent |  | x | x |  |  |  | negative |
| IV.15 | Ishihara et al. (2016) (66) | 2016 | ZEPHYR | DES | 202 lesions / ns | ns | 2-year restenosis rate |  |  | x |  | x |  | neutral |
| IV.16 | Ishihara et al. (2016) (67) | 2016 | none | DES (Zilver PTX) vs. BMS (SMART) | 5 / 5 | 36.1 months | thrombus presence, recurrence of symptoms, presence and location of organic stenosis |  |  | x |  | x |  | indecisive |
| IV.17 | Kang et al. (2016) (68) | 2016 | none | DES (Zilver PTX) | 87 / 78 | ns | primary patency | x |  | x |  | x |  | negative |
| IV.18 | Müller-Hülsbeck et al. (2016) (69) | 2016 | MAJESTIC | DES (Eluvia) | 57 / 57 | ns | duplex ultrasound peak systolic velocity ratio ≤2.5, absence of target lesion revascularization or bypass | x | x | x |  |  |  | positive |
| IV.19 | Takahara et al. (2016) (70) | 2016 | ZEPHYR | DES (Zilver PTX) | 495 / 495 | 24 months | re-re-EVT, re-EVT | x | x | x |  |  |  | indecisive |
| IV.20 | Wooster et al. (2016) (71) | 2016 | none | DES (Zilver PTX) | 30 / 30 | 62 months | primary and secondary patency | x | x | x |  |  |  | indecisive |
| IV.21 | Yokoi et al. (2016) (72) | 2016 | none | DES (Zilver PTX) | 907 / 907 | ns | target lesion revascularization, clinical benefit, patency | x | x | x |  |  |  | positive |
| IV.22 | Miki et al. (2017) (73) | 2017 | none | DES | 20 (20 lesions) / 10 / 10 vs. 10 | 6 months | lumen VI, minimum lumen diameter, amputations, deaths, TLR | x |  | x |  | x |  | positive |
| IV.23 | Müller-Hülsbeck et al. (2017) (74) | 2017 | MAJESTIC | DES (Eluvia) | 57 / 57 | ns | primary patency |  | x | x |  |  |  | positive |
| IV.24 | Oberto et al. (2017) (75) | 2017 | none | DES (Zilver PTX) | 67 / 67 | ns | primary patency | x |  | x |  |  |  | indecisive |
| IV.25 | Ogawa et al. (2017) (76) | 2017 | none | DES (Zilver PTX) | 905 / 905 | ns | freedom from TLR, patency | x |  | x |  |  |  | positive |
| IV.26 | Schumann et al. (2017) (77) | 2017 | none | DES (Zilver PTX) vs. SES | 18 / 18 | before  and after angioplasty | reconstruction accuracy |  |  |  |  | x |  | indecisive |
| IV.27 | Zen et al. (2017) (78) | 2017 | none | DES (Zilver PTX) | 475 / 475 | ns | 12-month restenosis rate, MALE | x | x | x |  | x |  | indecisive |
| IV.28 | Kichikawa et al. (2018) (79) | 2018 | Japan PMS | DES (Zilver PTX) | 905 / 905 | 2 years | patency, TLR |  |  |  |  | x |  | indecisive |
| IV.29 | Hoyt et al. (2019) (80) | 2019 | DESPERADO-SFA | DES (Zilver PTX) | 13 / 13 | 12 months | patency | x | x |  |  | x |  | indecisive |
| IV.30 | Huntress et al. (2019) (81) | 2019 | none | DES | 32 / 32 | mean 19.3 months (IQR 7-27.1 months) | changes in WIfI scores, patency rates, freedom from major amputation, target lesion recurrence, all-cause mortality | x |  | x |  | x |  | positive |
| IV.31 | Ichihashi et al. (2019) (82) | 2019 | none | DES (Zilver PTX) | 220 / 220 | median  19 months (IQR 1.1, 70.9) | primary patency, TLR, overall survival | x |  | x |  | x |  | indecisive |
| IV.32 | Kuntz et al. (2019) (83) | 2019 | none | DES (Eluvia, Zilver PTX) vs. SES vs. BMS | 9 / 2* / 2* vs. 10* vs. 2* | <90 days, >=90 days | radiography, micro–computed tomography, histological assessment |  |  |  |  | x |  | indecisive |
| IV.33 | Soga et al. (2019) (84) | 2019 | IMPERIAL | DES (Eluvia, Zilver PTX) | 56 / 56 | 1, 12 months | primary vessel patency, TLR, MAE, ABI, WIQ, QoL, technical success | x | x | x | x | x | x | positive |
| IV.34 | Tsukiyama et al. (2019) (85) | 2019 | SHIMEJI | DES (Zilver PTX) | 57 / 57 | 2, 6, 12 months | dominant NIC grade, expansion of thrombus (IS-Th grade), YP underneath stent | x |  | x |  | x |  | indecisive |
| IV.35 | Dake et al. (2020) (86) | 2020 | none | DES (Zilver PTX) | 2,227 / 2,227 | median 23.9 months (range: 0.03-60.8) | TLR |  |  |  |  | x |  | indecisive |
| IV.36 | Ducci et al. (2020) (87) | 2020 | none | DES (Zilver PTX) and medication | 40 / 40 | 12 months | clinical outcomes (TLR, mortality, subdural hematoma, major ischemic stroke, major amputation) | x | x |  |  | x | x | indecisive |
| IV.37 | Fanelli et al. (2020) (88) | 2020 | 3D (Double Drug Dose) | DES (Zilver PTX) vs. DCB | 15 / 15 | 1, 6, 12, 24 months | primary patency |  |  |  |  | x |  | indecisive |
| IV.38 | Golzar et al. (2020) (89) | 2020 | IMPERIAL | DES (Eluvia) vs. DCB | 50 / 50 | 12 months | clinical outcome, QoL, freedom from MAE  (all cause death, major amputation, TLR, stent thrombosis), procedural/technical success with no MAE (angiographic stenosis) | x | x | x | x | x |  | positive |
| IV.39 | Miki et al. (2020) (90) | 2020 | none | DES (Zilver PTX) | 40 / 40 | 6 months | OCT-derived neointimal VI, procedural success, procedure-related complications, angiography, OCT, TLR, primary patency | x |  | x |  | x |  | negative |
| IV.40 | Müller-Hülsbeck et al. (2020) (91) | 2020 | IMPERIAL | DES (Eluvia, Zilver PTX) | 465 / 465 | 24 months | primary patency, patient outcome assessment, safety, clinical outcomes (Rutherford, TLR), ultrasound imaging | x |  | x |  | x |  | positive |
| IV.41 | Iida et al. (2021) (92) | 2021 | IMPERIAL | DES (Eluvia, Zilver PTX) | 84 / 84 | 24 months (730 ± 30 days) | patency, TLR, CD-TLR, target limb amputation, stent thrombosis, death, ABI, WIQ, QoL, medication use | x |  | x | x | x |  | positive |
| IV.42 | Kum et al. (2021) (93) | 2021 | none | DES (Eluvia) | 64 / 64 | mean 11.3 ± 7.8 months | primary patency,  30-days complication rate, technical success, CD-TLR, limb salvage, survival, AFS, wound healing, clinical improvement | x | x | x |  | x | x | indecisive |
| IV.43 | Kwon et al. (2021) (94) | 2021 | none | DES (Eluvia, Zilver PTX) | 34 / 34 | 3, 6, 12 months | primary patency, TLR, adverse events (stroke, sudden cardiac arrest) |  | x | x |  | x |  | positive |
| IV.44 | Marples et al. (2021) (95) | 2021 | none | DES (Zilver PTX) vs. stent graft | 61 procedures / 25 procedures / 25 vs. 36 (procedures) | stent graft group: mean 27.25 months, stent graft + DES group: mean 18.46 months | primary patency |  |  |  |  | x |  | indecisive |
| IV.45 | Stavroulakis et al. (2021) (96) | 2021 | none | DES (Eluvia) | 130 / 130 | mean  24 months (IQR 16-34 months) | primary patency, secondary patency,  CD-TLR, freedom from major amputation, freedom from major adverse cardiac and cerebrovascular events, freedom from surgical conversion, incidence of arterial wall degeneration | x | x | x |  | x |  | indecisive |
| IV.46 | Cheban et al. (2022) (97) | 2022 | none | DES (Zilver PTX) vs. DES (Zilver PTX) and fasciotomy of Gunter's canal | 60 / 60 | 6, 12 months | primary patency, primary-assisted patency, secondary patency, stent fracture, technical success, TLR, MACE, MALE |  | x |  |  | x | x | positive |
| IV.47 | Giannopoulos et al. (2022) (98) | 2022 | none | DES (Eluvia) and DCB | 22 / 22 | mean 15.2 months | procedural success, MALE, TLR, all-cause mortality, limb loss | x | x | x |  | x | x | indecisive |
| IV.48 | Gray et al. (2022) (99) | 2022 | IMPERIAL | DES (Eluvia, Zilver PTX) | 10,000 / 10,000 | 24 months | patency, TLR, amputation, death | x |  |  |  | x | x | positive |
| IV.49 | Vu et al. (2022) (100) | 2022 | XLPAD | DES vs. BMS vs. DCB vs. covered stent | 2,895 / 239 | 6, 12 months | MALE, all-cause death, target limb repeat revascularization, major amputation | x |  | x |  | x |  | indecisive |
| **Legend:** 1 – after recruitment; 2 – in comparative studies; 3 – only in RCTs; 4 – study explicitly described as "longitudinal" in article; ABI – ankle-brachial index; AE – adverse events; AFS – amputation free survival; AFS – amputation-free survival; ALI – acute limb ischemia; BES – balloon expandable stent; BMS – bare metal stent; BSX – prosthetic bypass graft; CD-TLR – clinical-driven target lesion revascularization; CM – (co)morbidity; DCB – drug-coated balloon; DES – drug-eluting stent; ESL – extrastent lumen; EVR – endovascular revascularization; EVT – endovascular therapy;  ff-MA – freedom from major amputation; ff-TVR – freedom from target vessel revascularization; IQR – inter-quartile range; ISR – in-stent restenosis;  IS-Th – intra-stent thrombus; M – mortality; MACE – major adverse cardiovascular event; MALE – major adverse limb events; MCE – severe adverse cardiac event; NIC – neointimal coverage; ns – not stated; OCT – optical coherence tomography; PLIA – peristrut low-intensity area; PTA – percutaneous transluminal angioplasty / plain balloon angioplasty; QoL – quality of life; SES – self-expandable stent; SURE – surrogate endpoints; VI – volume index; WIfI – Wound, Ischemia, and foot Infection; WiQ – Walking Impairment Questionnaire | | | | | | | | | | | | | | |

**References**

1. Duda SH, Bosiers M, Lammer J, Scheinert D, Zeller T, Oliva V, et al. Drug-eluting and bare nitinol stents for the treatment of atherosclerotic lesions in the superficial femoral artery: long-term results from the SIROCCO trial. *Journal of Endovascular Therapy* (2006) **13**:701–10. doi:10.1583/05-1704.1

2. Dake MD, Ansel GM, Jaff MR, Ohki T, Saxon RR, Smouse HB, et al. Paclitaxel-eluting stents show superiority to balloon angioplasty and bare metal stents in femoropopliteal disease: twelve-month Zilver PTX randomized study results. *Circulation: Cardiovascular Interventions* (2011) **4**:495–504. doi:10.1161/CIRCINTERVENTIONS.111.962324

3. Dake MD, Ansel GM, Jaff MR, Ohki T, Saxon RR, Smouse HB, et al. Durable clinical effectiveness with Paclitaxel-eluting stents in the femoropopliteal artery: 5-year results of the Zilver PTX randomized trial. *Circulation* (2016) **133**:1472–83. doi:10.1161/CIRCULATIONAHA.115.016900

4. Miki K, Fujii K, Shibuya M, Fukunaga M, Imanaka T, Tamaru H, et al. Comparing the vascular response in implantation of self-expanding, bare metal nitinol stents or Paclitaxel-eluting nitinol stents in superficial femoral artery lesions: a serial optical frequency domain imaging study. *EuroIntervention* (2016) **12**:1551–8. doi:10.4244/EIJ-D-15-00399

5. Bausback Y, Wittig T, Schmidt A, Zeller T, Bosiers M, Peeters P, et al. Drug-eluting stent versus drug-coated balloon revascularization in patients with femoropopliteal arterial disease. *Journal of the American College of Cardiology* (2019) **73**:667–79. doi:10.1016/j.jacc.2018.11.039

6. Dake MD, Ansel GM, Bosiers M, Holden A, Iida O, Jaff MR, et al. Paclitaxel-coated Zilver PTX drug-eluting stent treatment does not result in increased long-term all-cause mortality compared to uncoated devices. *CardioVascular and Interventional Radiology* (2020) **43**:8–19. doi:10.1007/s00270-019-02324-4

7. Liistro F, Angioli P, Porto I, Ducci K, Falsini G, Ventoruzzo G, et al. Drug-eluting balloon versus drug-eluting stent for complex femoropopliteal arterial lesions: the DRASTICO study. *Journal of the American College of Cardiology* (2019) **74**:205–15. doi:10.1016/j.jacc.2019.04.057

8. Bosiers M, Setacci C, Donato G de, Torsello G, Silveira PG, Deloose K, et al. ZILVERPASS study: Zilver PTX stent vs bypass surgery in femoropopliteal lesions. *Journal of Endovascular Therapy* (2020) **27**:287–95. doi:10.1177/1526602820902014

9. Falkowski A, Bogacki H, Szemitko M. Assessment of mortality and factors affecting outcome of use of Paclitaxel-coated stents and bare metal stents in femoropopliteal pad. *Journal of clinical medicine* (2020) **9**:1–11.

10. Gouëffic Y, Sauguet A, Desgranges P, Feugier P, Rosset E, Ducasse E, et al. A polymer-free Paclitaxel-eluting stent versus a bare-metal stent for de novo femoropopliteal lesions: the BATTLE trial. *JACC. Cardiovascular interventions* (2020) **13**:447–57. doi:10.1016/j.jcin.2019.12.028

11. Ishihara T, Iida O, Awata M, Nanto K, Nanto S, Uematsu M. Angioscopic assessment of early phase arterial repair after Paclitaxel-coated nitinol drug-eluting stent implantation in the superficial femoral artery. *Circulation Journal* (2013) **77**:1838–43. doi:10.1253/circj.CJ-13-0012

12. Ishihara T, Iida O, Awata M, Nanto K, Shiraki T, Okamoto S, et al. Extensive arterial repair one year after Paclitaxel-coated nitinol drug-eluting stent vs. bare-metal stent implantation in the superficial femoral artery. *Cardiovascular Intervention and Therapeutics* (2014) **30**:51–6. doi:10.1007/s12928-014-0287-z

13. Kozuki A, Shinke T, Otake H, Kijima Y, Masano T, Nagoshi R, et al. Optical coherence tomography study of chronic-phase vessel healing after implantation of bare metal and Paclitaxel-eluting self-expanding nitinol stents in the superficial femoral artery. *Journal of Cardiology* (2016) **67**:424–9. doi:10.1016/j.jjcc.2015.06.011

14. Murata N, Takahara M, Soga Y, Nakano M, Yamauchi Y, Zen K, et al. Drug-eluting stent vs percutaneous transluminal angioplasty for treatment of femoropopliteal in-stent restenosis: results from a retrospective 1-year multicenter study. *Journal of endovascular therapy. 23 (4) (pp 642-647), 2016. Date of publication: august 2016.* (2016). doi:10.1177/1526602816642195

15. Mori S, Hirano K, Yamauchi Y, Hayashi E, Doijiri T, Takamura T, et al. Penetration rate of the placement of a drug-eluting stent for the treatment of superficial femoral artery lesions in Japan. *Heart and Vessels* (2017) **32**:1093–8. doi:10.1007/s00380-017-0982-7

16. Björkman P, Auvinen T, Hakovirta H, Romsi P, Turtiainen J, Manninen H, et al. Drug-eluting stent shows similar patency results as prosthetic bypass in patients with femoropopliteal occlusion in a randomized trial. *European Journal of Vascular and Endovascular Surgery* (2019) **58**:e353‐e354. doi:10.1016/j.ejvs.2019.06.980

17. Saratzis A, Rudarakanchana N, Patel S, Diamantopoulos A, Lea T, Corbo B, et al. Interwoven nitinol stents versus drug eluting stents in the femoro-popliteal segment: a propensity matched analysis. *European journal of vascular and endovascular surgery the official journal of the European Society for Vascular Surgery* (2019) **58**:719–27. doi:10.1016/j.ejvs.2019.06.012

18. Aihara H, Higashitani M, Takimura H, Tobita K, Jujo K, Hozawa K, et al. Differences in intravascular ultrasound measurement values between treatment modalities for restenosis in femoropopliteal lesions. *Circulation Journal* (2020) **84**:1320–9. doi:10.1253/circj.CJ-20-0218

19. Iida O, Takahara M, Soga Y, Fujihara M, Kawasaki D, Hirano K, et al. A novel angiographic risk score for femoropopliteal interventions. *Journal of Endovascular Therapy* (2020). doi:10.1177/1526602820948472

20. Kobayashi T, Hamamoto M, Okazaki T, Hasegawa M, Fujiwara T, Takahashi S. Effectiveness of combined superficial femoral artery endovascular therapy with popliteal-to-distal bypass: a paradigm shift in surgical open bypass for chronic limb-threatening ischemia. *Vascular* (2021) **29**:905–12. doi:10.1177/1708538120981224

21. Lee YJ, Kook H, Ko YG, Yu CW, Joo HJ, Ahn CM, et al. Drug eluting stent vs. drug coated balloon for native femoropopliteal artery disease: a two centre experience. *European Journal of Vascular and Endovascular Surgery* (2021) **61**:287–95. doi:10.1016/j.ejvs.2020.10.008

22. Yoshioka N, Tokuda T, Koyama A, Yamada T, Nishikawa R, Shimamura K, et al. Clinical outcomes and predictors of restenosis in patients with femoropopliteal artery disease treated using polymer-coated Paclitaxel-eluting stents or drug-coated balloons. *Heart and Vessels* (2022) **37**:555–66. doi:10.1007/s00380-021-01941-9

23. Dake MD, Ansel GM, Jaff MR, Ohki T, Saxon RR, Smouse HB, et al. Sustained safety and effectiveness of Paclitaxel-eluting stents for femoropopliteal lesions: 2-year follow-up from the Zilver PTX randomized and single-arm clinical studies. *Journal of the American College of Cardiology* (2013) **61**:2417–27. doi:10.1016/j.jacc.2013.03.034

24. Miki K, Fujii K, Kawasaki D, Fukunaga M, Nishimura M, Horimatsu T, et al. Effect of bare-metal nitinol stent implantation and Paclitaxel-eluting nitinol stent implantation on vascular response in the superficial femoral artery lesion assessed on intravascular ultrasound. *Circulation Journal* (2014) **78**:1451–8. doi:10.1253/circj.CJ-13-1508

25. Kay M, Rogoveanu R, Hodson J, Tallowin S, Hopkins J, Duddy M, et al. Factors affecting the results of superficial femoral artery stenting. *Vascular and Endovascular Surgery* (2015) **49**:228–35. doi:10.1177/1538574415614405

26. Ansel GM, Jaff MR, Popma JJ, Battisti AJ, Lottes AE, Harnish P, et al. A quantitative angiographic comparison of restenotic tissue following placement of drug-eluting stents and bare metal stents in symptomatic patients with femoropopliteal disease. *Journal of Endovascular Therapy* (2017) **24**:499–503. doi:10.1177/1526602817708778

27. Giordano A, Ferraro P, Corcione N, Messina S, Maresca G, Coscioni E, et al. Comparison of Biolimus versus Everolimus for drug-eluting stents in the percutaneous treatment of infra-inguinal arterial disease. *Current Vascular Pharmacology* (2017) **15**:257–64. doi:10.2174/1570161115666170123094523

28. Freisinger E, Koeppe J, Gerss J, Goerlich D, Malyar NM, Marschall U, et al. Mortality after use of Paclitaxel-based devices in peripheral arteries: a real-world safety analysis. *European Heart Journal* (2020) **41**:3732–9. doi:10.1093/eurheartj/ehz698

29. Haine A, Schmid MJ, Schindewolf M, Lenz A, Bernhard SM, Drexel H, et al. Comparison between interwoven nitinol and drug eluting stents for endovascular treatment of femoropopliteal artery disease. *European Journal of Vascular and Endovascular Surgery* (2019) **58**:865–73. doi:10.1016/j.ejvs.2019.09.002

30. Katsuki T, Takahara M, Soga Y, Okamoto S, Iida O, Fujihara M, et al. Mortality risk following application of a Paclitaxel-coated stent in femoropopliteal lesions. *Journal of Endovascular Therapy* (2019) **26**:593–9. doi:10.1177/1526602819870309

31. Kibrik P, Victory J, Patel R, Chait J, Alsheekh A, Aurshina A, et al. A real-world experience of drug eluting and non-drug eluting stents in lower extremity peripheral arterial disease. *Vascular* (2019) **27**:648–52. doi:10.1177/1708538119850445

32. Kumins NH, King AH, Ambani RN, Thomas JP, Bose S, Shishehbor MH, et al. Paclitaxel-coated peripheral artery devices are not associated with increased mortality. *Journal of Vascular Surgery* (2020) **72**:968–76. doi:10.1016/j.jvs.2019.10.100

33. Phair J, Carnevale M, Lipsitz EC, Shariff S, Scher L, Garg K. Primary patency of long-segment femoropopliteal artery lesions in patients with peripheral arterial occlusive disease treated with Paclitaxel-eluting technology. *Annals of Vascular Surgery* (2020) **66**:595–600. doi:10.1016/j.avsg.2019.11.044

34. Secemsky EA, Kundi H, Weinberg I, Jaff MR, Krawisz A, Parikh SA, et al. Association of survival with femoropopliteal artery revascularization with drug-coated devices. *JAMA Cardiology* (2019) **4**:332–40. doi:10.1001/jamacardio.2019.0325

35. Behrendt CA, Sedrakyan A, Peters F, Kreutzburg T, Schermerhorn M, Bertges DJ, et al. Long term survival after femoropopliteal artery revascularisation with Paclitaxel coated devices: a propensity score matched cohort analysis. *European Journal of Vascular and Endovascular Surgery* (2020) **59**:587–96. doi:10.1016/j.ejvs.2019.12.034

36. Bertges DJ, Sedrakyan A, Sun T, Eslami MH, Schermerhorn M, Goodney PP, et al. Mortality after Paclitaxel coated balloon angioplasty and stenting of superficial femoral and popliteal artery in the vascular quality initiative. *Circulation: Cardiovascular Interventions* (2020):e008528. doi:10.1161/CIRCINTERVENTIONS.119.008528

37. Cho S, Han A, Ahn S, Min S, Ha J, Jae HJ, et al. Directional atherectomy for treating in-stent restenosis of the superficial femoral artery. *Vascular Specialist International* (2020) **36**:136–43. doi:10.5758/vsi.200017

38. Draxler MS, Al-Adas Z, Abbas D, Kavousi Y, Kabbani LS, Lin JC, et al. Outcome benefit of arterial duplex stent imaging after superficial femoral artery stent implantation. *Journal of Vascular Surgery* (2021) **73**:179–88. doi:10.1016/j.jvs.2020.02.055

39. Mathlouthi A, Yei KS, Naazie I, Bertges DJ, Malas MB. Increased mortality with Paclitaxel-eluting stents is driven by lesion length. *Journal of Vascular Surgery* (2021) **73**:548-553.e2. doi:10.1016/j.jvs.2020.05.061

40. Rocha-Singh KJ, Duval S, Jaff MR, Schneider PA, Ansel GM, Lyden SP, et al. Mortality and Paclitaxel-coated devices: an individual patient data meta-analysis. *Circulation* (2020) **141**:1859–69. doi:10.1161/CIRCULATIONAHA.119.044697

41. Saratzis A, Lea T, Yap T, Batchelder A, Thomson B, Saha P, et al. Paclitaxel and mortality following peripheral angioplasty: an adjusted and case matched multicentre analysis. *European Journal of Vascular and Endovascular Surgery* (2020) **60**:220–9. doi:10.1016/j.ejvs.2020.04.008

42. Weissler EH, Annapureddy A, Wang Y, Secemsky EA, Shishehbor MH, Mena-Hurtado C, et al. Paclitaxel-coated devices in the treatment of femoropopliteal stenosis among patients >=65 years old: an ACC PVI Registry Analysis. *American Heart Journal* (2021) **233**:59–67. doi:10.1016/j.ahj.2020.12.004

43. Altaf N, Ariyaratne TV, Peacock A, Deltetto I, El-Hoss J, Thomas S, et al. A budget impact model for the use of drug-eluting stents in patients with symptomatic lower-limb peripheral arterial disease: an Australian perspective. *CardioVascular and Interventional Radiology* (2021) **44**:1375–83. doi:10.1007/s00270-021-02848-8

44. Barenbrock H, Feld J, Lakomek A, Volkery K, Koppe J, Makowski L, et al. Sex-related differences in outcome after endovascular revascularization for lower extremity artery disease: a single-centre analysis of a specialized vascular unit. *Vasa - European Journal of Vascular Medicine* (2022) **51**:29–36. doi:10.1024/0301-1526/a000978

45. Choi H, Lee H, Lee S-S, Ahn J, Joh JH, Lee M-Y. Association of mortality with drug-coated devices in femoropopliteal artery based on the nationwide data. *Annals of surgical treatment and research* (2021) **101**:20–7. doi:10.4174/astr.2021.101.1.20

46. Gahide G, Phaneuf SC, Cossette M, Banine A, Budimir M, Maghsoudloo K, et al. Paclitaxel and mortality in patients with claudication and de novo femoropopliteal lesions: a historical cohort study. *CVIR Endovascular* (2021) **4**:65. doi:10.1186/s42155-021-00255-1

47. Kumins NH, King AH, Ambani RN, Cho JS, Harth KC, Wong VL, et al. Paclitaxel-coated peripheral arterial devices are associated with improved overall survival and limb salvage in patients with chronic limb-threatening ischemia. *Journal of Vascular Surgery* (2021) **74**:1682-1688.e1. doi:10.1016/j.jvs.2021.05.035

48. Nakamura M, Takata M, Yokoi H, Ueno T, Suzuki Y, Ikeda K, et al. An individual-level meta-analysis using real-world and pivotal studies on mortality from the use of Paclitaxel-containing devices in Japanese femoropopliteal disease patients. *Circulation Journal* (2021) **85**:2137–45. doi:10.1253/circj.CJ-21-0171

49. Secemsky EA, Barrette E, Bockstedt L, Bonaca MP, Hess CN, Hanson T, et al. Long-term safety of drug-coated devices for peripheral revascularisation. *EuroIntervention* (2021) **17**:590–8. doi:10.4244/EIJ-D-20-01018

50. Miura T, Miyashita Y, Hozawa K, Doijiri T, Kato T, Hayakawa N, et al. Cilostazol effectiveness in reducing drug-coated stent restenosis in the superficial femoral artery: the ZERO study. *PloS one* (2022) **17**:e0270992. doi:10.1371/journal.pone.0270992

51. Suzuki K, Ueshima D, Higashitani M, Yamauchi Y, Hozawa K, Hayakawa N, et al. Two-year results of endovascular therapy for femoropopliteal artery disease in Japan during the introduction of drug-eluting devices. *Cardiovascular Intervention and Therapeutics* (2022). doi:10.1007/s12928-022-00873-z

52. Feiring AJ, Wesolowski AA. Antegrade popliteal artery approach for the treatment of critical limb ischemia in patients with occluded superficial femoral arteries. *Catheterization and Cardiovascular Interventions* (2007) **69**:665–70. doi:10.1002/ccd.21069

53. Dake MD, Scheinert D, Tepe G, Tessarek J, Fanelli F, Bosiers M, et al. Nitinol stents with polymer-free Paclitaxel coating for lesions in the superficial femoral and popliteal arteries above the knee: twelve-month safety and effectiveness results from the Zilver PTX single-arm clinical study. *Journal of Endovascular Therapy* (2011) **18**:613–23. doi:10.1583/11-3560.1

54. Karnabatidis D, Spiliopoulos S, Pastromas G, Katsanos K, Siablis D. Endovascular management of the arteria profunda femoralis: long-term angiographic and clinical outcomes. *CardioVascular and Interventional Radiology* (2012) **35**:1016–22. doi:10.1007/s00270-011-0284-x

55. Lammer J, Bosiers M, Zeller T, Schillinger M, Boone E, Zaugg MJ, et al. First clinical trial of nitinol self-expanding Everolimus-eluting stent implantation for peripheral arterial occlusive disease. *Journal of Vascular Surgery* (2011) **54**:394–401. doi:10.1016/j.jvs.2011.01.047

56. Bosiers M, Peeters P, Tessarek J, Deloose K, Strickler S. The Zilver PTX single arm study: 12-month results from the TASC C/D lesion subgroup. *Journal of Cardiovascular Surgery* (2013) **54**:115–22.

57. Fanelli F, Di Primo M, Boatta E, Johnston K, Sapoval M. Drug-eluting nitinol stent treatment of the superficial femoral artery and above-the-knee popliteal artery (the Zilver PTX single-arm clinical study): a comparison between diabetic and nondiabetic patients. *CardioVascular and Interventional Radiology* (2013) **36**:1232–40. doi:10.1007/s00270-012-0543-5

58. Fujihara M, Utsunomiya M, Higashimori A, Yokoi Y, Nakamura M. Outcomes of Zilver PTX stent implantation for the treatment of complex femoropopliteal artery disease. *Heart and Vessels* (2016) **31**:152–7. doi:10.1007/s00380-014-0596-2

59. Leopardi M, Houbballah R, Becquemin JP. Effectiveness of Zilver PTX eluting stent in TASC C/D lesions and restenosis. *Journal of Cardiovascular Surgery* (2014) **55**:229–34.

60. Ohki T, Yokoi H, Kichikawa K, Kimura T, Snyder SA, Ragheb AO, et al. Two-year analysis of the Japanese cohort from the Zilver PTX randomized controlled trial supports the validity of multinational clinical trials. *Journal of Endovascular Therapy* (2014) **21**:644–53. doi:10.1583/14-4753.1

61. Iida O, Takahara M, Soga Y, Nakano M, Yamauchi Y, Zen K, et al. 1-year results of the ZEPHYR registry (Zilver PTX for the femoral artery and proximal popliteal artery) predictors of restenosis. *JACC: Cardiovascular Interventions* (2015) **8**:1105–12. doi:10.1016/j.jcin.2015.03.022

62. Tomoi Y, Kuramitsu S, Soga Y, Aihara H, Ando K, Nobuyoshi M. Vascular response after Zilver PTX stent implantation for superficial femoral artery lesions: serial optical coherence tomography findings at 6 and 12 months. *Journal of Endovascular Therapy* (2015) **22**:41–7. doi:10.1177/1526602814566577

63. Banerjee S, Sarode K, Mohammad A, Gigliotti O, Baig MS, Tsai S, et al. Femoropopliteal artery stent thrombosis: report from the excellence in peripheral artery disease registry. *Circulation: Cardiovascular Interventions* (2016) **9**:e002730. doi:10.1161/CIRCINTERVENTIONS.115.002730

64. Iida O, Takahara M, Soga Y, Hirano K, Yamauchi Y, Zen K, et al. Incidence and its characteristics of repetition of reintervention after drug-eluting stent implantation for femoropopliteal lesion. *Journal of Vascular Surgery* (2016) **64**:1691-1695.e1. doi:10.1016/j.jvs.2016.05.074

65. Iida O, Takahara M, Soga Y, Hirano K, Yamauchi Y, Zen K, et al. The characteristics of in-stent restenosis after drug-eluting stent implantation in femoropopliteal lesions and 1-year prognosis after repeat endovascular therapy for these lesions. *JACC: Cardiovascular Interventions* (2016) **9**:828–34. doi:10.1016/j.jcin.2016.01.007

66. Ishihara T, Takahara M, Iida O, Soga Y, Hirano K, Yamauchi Y, et al. Comparable 2-year restenosis rates following subintimal and intraluminal drug-eluting stent implantation for femoropopliteal chronic total occlusion. *Journal of Endovascular Therapy* (2016) **23**:889–95. doi:10.1177/1526602816666261

67. Ishihara T, Iida O, Okamoto S, Fujita M, Masuda M, Nanto K, et al. Potential mechanisms of in-stent occlusion in the femoropopliteal artery: an angioscopic assessment. *Cardiovascular Intervention and Therapeutics* (2017) **32**:313–7. doi:10.1007/s12928-016-0411-3

68. Kang WY, Campia U, Didier RJ, Kiramijyan S, Koifman E, Negi SI, et al. A single center experience of Zilver PTX for femoro-popliteal lesions. *Cardiovascular Revascularization Medicine* (2016) **17**:399–403. doi:10.1016/j.carrev.2016.02.004

69. Müller-Hülsbeck S, Keirse K, Zeller T, Schroe H, Diaz-Cartelle J. Twelve-month results from the MAJESTIC trial of the Eluvia Paclitaxel-eluting stent for treatment of obstructive femoropopliteal disease. *Journal of Endovascular Therapy* (2016) **23**:701–7. doi:10.1177/1526602816650206

70. Takahara M, Yokoi H, Uematsu M, Azuma N, Nakahama M, Tazaki J, et al. Incidence and its characteristics of repetition of reintervention after drug-eluting stent implantation for femoropopliteal lesion. *Journal of Vascular Surgery* (2016) **64**:1691. doi:10.1016/j.jvs.2016.05.074

71. Wooster M, Dansey K, Shames M. Early post-registry experience with drug-eluting stents in the superficial femoral artery. *Vascular and Endovascular Surgery* (2016) **50**:80–3. doi:10.1177/1538574416628651

72. Yokoi H, Ohki T, Kichikawa K, Nakamura M, Komori K, Nanto S, et al. Zilver PTX post-market surveillance study of Paclitaxel-eluting stents for treating femoropopliteal artery disease in Japan: 12-month results. *JACC: Cardiovascular Interventions* (2016) **9**:271–7. doi:10.1016/j.jcin.2015.09.035

73. Miki K, Fujii K, Shibuya M, Fukunaga M, Imanaka T, Kawai K, et al. Impact of stent diameter on vascular response after self-expanding Paclitaxel-eluting stent implantation in the superficial femoral artery. *Journal of Cardiology* (2017) **70**:346–52. doi:10.1016/j.jjcc.2016.12.011

74. Müller-Hülsbeck S, Keirse K, Zeller T, Schroe H, Diaz-Cartelle J. Long-term results from the MAJESTIC trial of the Eluvia Paclitaxel-eluting stent for femoropopliteal treatment: 3-year follow-up. *CardioVascular and Interventional Radiology* (2017) **40**:1832–8. doi:10.1007/s00270-017-1771-5

75. Oberto S, Cetta F, Trabattoni P, Zoli S, Tavano D, Rossi F, et al. Comparison of SFA lesion treatment with Zilver PTX in diabetics vs. non-diabetics: 2-year clinical and functional results. *Journal of Cardiovascular Surgery* (2017) **58**:565–73. doi:10.23736/S0021-9509.16.08563-3

76. Ogawa Y, Yokoi H, Ohki T, Kichikawa K, Nakamura M, Komori K, et al. Impact of chronic renal failure on safety and effectiveness of Paclitaxel-eluting stents for femoropopliteal artery disease: subgroup analysis from Zilver PTX post-market surveillance study in Japan. *CardioVascular and Interventional Radiology* (2017) **40**:1669–77. doi:10.1007/s00270-017-1673-6

77. Schumann S, Gokgol C, Diehm N, Buchler P, Zheng G. Effect of stent implantation on the deformations of the superficial femoral artery and popliteal artery: in vivo three-dimensional deformational analysis from two-dimensional radiographs. *Journal of Vascular and Interventional Radiology* (2017) **28**:142–6. doi:10.1016/j.jvir.2016.04.023

78. Zen K, Takahara M, Iida O, Soga Y, Kawasaki D, Nanto S, et al. Drug-eluting stenting for femoropopliteal lesions, followed by Cilostazol treatment, reduces stent restenosis in patients with symptomatic peripheral artery disease. *Journal of Vascular Surgery* (2017) **65**:720–5. doi:10.1016/j.jvs.2016.10.098

79. Kichikawa K, Ichihashi S, Yokoi H, Ohki T, Nakamura M, Komori K, et al. Zilver PTX post-market surveillance study of Paclitaxel-eluting stents for treating femoropopliteal artery disease in Japan: 2-year results. *CardioVascular and Interventional Radiology* (2019) **42**:358–64. doi:10.1007/s00270-018-2110-1

80. Hoyt T, Feldman MD, Okutucu S, Lendel V, Marmagkiolis K, McIntosh V, et al. Assessment of vascular patency and inflammation with intravascular optical coherence tomography in patients with superficial femoral artery disease treated with Zilver PTX stents. *Cardiovascular Revascularization Medicine* (2020) **21**:101–7. doi:10.1016/j.carrev.2019.07.009

81. Huntress LA, Fereydooni A, Dardik A, Nassiri N. Endovascular revascularization incorporating infrapopliteal coronary drug-eluting stents improves clinical outcomes in patients with critical limb ischemia and tissue loss. *Annals of Vascular Surgery* (2020) **63**:234–40. doi:10.1016/j.avsg.2019.07.011

82. Ichihashi S, Shibata T, Fujimura N, Nagatomi S, Yamamoto H, Kyuragi R, et al. Vessel calcification as a risk factor for in-stent restenosis in complex femoropopliteal lesions after Zilver PTX Paclitaxel-coated stent placement. *Journal of Endovascular Therapy* (2019) **26**:613–20. doi:10.1177/1526602819860124

83. Kuntz SH, Torii S, Jinnouchi H, Cornelissen A, Sakamoto A, Sato Y, et al. Pathology and multimodality imaging of acute and chronic femoral stenting in humans. *JACC: Cardiovascular Interventions* (2020) **13**:418–27. doi:10.1016/j.jcin.2019.10.060

84. Soga Y, Fujihara M, Iida O, Kawasaki D, Hirano K, Yokoi H, et al. Japanese patients treated in the IMPERIAL randomized trial comparing Eluvia and Zilver PTX stents. *CardioVascular and Interventional Radiology* (2020) **43**:215–22. doi:10.1007/s00270-019-02355-x

85. Tsukiyama Y, Shinke T, Ishihara T, Otake H, Terashita D, Kozuki A, et al. Vascular response to Paclitaxel-eluting nitinol self-expanding stent in superficial femoral artery lesions: post-implantation angioscopic findings from the SHIMEJI trial (Suppression of vascular wall Healing after IMplantation of drug Eluting peripheral stent in Japanese patients with the Infra inguinal lesion: serial angioscopic observation). *The international journal of cardiovascular imaging* (2019) **35**:1777–84. doi:10.1007/s10554-019-01638-1

86. Dake MD, Fanelli F, Lottes AE, O'Leary EE, Reichert H, Jiang X, et al. Prediction model for freedom from TLR from a multi-study analysis of long-term results with the Zilver PTX drug-eluting peripheral stent. *CardioVascular and Interventional Radiology* (2020). doi:10.1007/s00270-020-02648-6

87. Ducci K, Liistro F, Porto I, Ventoruzzo G, Angioli P, Falsini G, et al. Ticagrelor versus clopidogrel in patients undergoing implantation of Paclitaxel-eluting stent in the femoropopliteal district: a randomized pilot study using frequency-domain optical coherence tomography. *International journal of cardiology* (2020) **304**:192–7. doi:10.1016/j.ijcard.2020.01.024

88. Fanelli F, Cannavale A, Citone M, Santoni M, Gazzetti M, Falcone GM, et al. Provisional stenting using the Zilver PTX drug-eluting stent after drug-coated balloon angioplasty: initial experience from the double drug dose "3D" study. *Journal of Endovascular Therapy* (2020) **27**:34–41. doi:10.1177/1526602819884062

89. Golzar J, Soga Y, Babaev A, Iida O, Kawasaki D, Bachinsky W, et al. Effectiveness and safety of a Paclitaxel-eluting stent for superficial femoral artery lesions up to 190 mm: one-year outcomes of the single-arm IMPERIAL long lesion substudy of the Eluvia drug-eluting stent. *Journal of Endovascular Therapy* (2020) **27**:296–303. doi:10.1177/1526602820901723

90. Miki K, Tanaka T, Yanaka K, Yoshihara N, Kimura T, Imanaka T, et al. Influence of self-expanding Paclitaxel-eluting stent sizing on neointimal hyperplasia in superficial femoral artery lesions. *Circulation Journal* (2020). doi:10.1253/circj.CJ-20-0470

91. Müller-Hülsbeck S, Benko A, Soga Y, Fujihara M, Iida O, Babaev A, et al. Two-year efficacy and safety results from the IMPERIAL randomized study of the Eluvia polymer-coated drug-eluting stent and the Zilver PTX polymer-free drug-coated stent. *CardioVascular and Interventional Radiology* (2021) **44**:368–75. doi:10.1007/s00270-020-02693-1

92. Iida O, Fujihara M, Kawasaki D, Mori S, Yokoi H, Miyamoto A, et al. 24-month efficacy and safety results from Japanese patients in the IMPERIAL randomized study of the Eluvia drug-eluting stent and the Zilver PTX drug-coated stent. *CardioVascular and Interventional Radiology* (2021) **44**:1367–74. doi:10.1007/s00270-021-02901-6

93. Kum S, Ipema J, Huizing E, Tan YK, Lim D, Lok IY, et al. Outcomes of the Paclitaxel-eluting Eluvia stent for long femoropopliteal lesions in Asian patients with predominantly chronic limb-threatening ischemia. *Vascular medicine (London, England)* (2021) **26**:267–72. doi:10.1177/1358863X20987894

94. Kwon LM, Hur S, Jae HJ, Min SK, Min SI, Ahn S, et al. One-year outcomes of two different Paclitaxel-eluting stents (Zilver PTX and Eluvia) for trans-atlantic inter-society consensus document (TASC) C/D obstructive femoropopliteal lesions. *Iranian Journal of Radiology* (2022) **19**:e111918. doi:10.5812/iranjradiol.111918

95. Marples R, Binks M, Spina R, Wright M, Huilgol R. Prophylactic Paclitaxel-eluting stent placement does not improve covered femoropopliteal stent patency. *Surgery Open Science* (2022) **7**:18–21. doi:10.1016/j.sopen.2021.09.004

96. Stavroulakis K, Torsello G, Bosiers M, Argyriou A, Tsilimparis N, Bisdas T. 2-year outcomes of the Eluvia drug-eluting stent for the treatment of complex femoropopliteal lesions. *JACC: Cardiovascular Interventions* (2021) **14**:692–701. doi:10.1016/j.jcin.2021.01.026

97. Cheban AV, Osipova OS, Ignatenko PV, Bugurov SV, Gostev AA, Saaya SB, et al. One-year results of long femoropopliteal lesions stenting with fasciotomy lamina vastoadductoria. *medRxiv* (2022). doi:10.1101/2022.03.28.22272737

98. Giannopoulos S, Secemsky EA, Schneider PA, Armstrong EJ. Concomitant drug-coated balloon angioplasty with bail-out use of Eluvia drug-eluting stent: Is there any downside to a double dose of Paclitaxel? *The Journal of invasive cardiology* (2022) **34**:E469-E476.

99. Gray WA, Griffiths RI, Elroy PW, Amorosi SL, McGovern AM, Jaff MR, et al. Cost-effectiveness of a Paclitaxel-eluting stent (Eluvia) compared to Zilver PTX for endovascular femoropopliteal intervention. *Journal of Medical Economics* (2022) **25**:880–7. doi:10.1080/13696998.2022.2088965

100. Vu MH, Sande-Docor GM, Liu Y, Tsai S, Patel M, Metzger C, et al. Endovascular treatment and outcomes for femoropopliteal in-stent restenosis: insights from the XLPAD registry. *Journal of Interventional Cardiology* (2022) **2022**:5935039. doi:10.1155/2022/5935039
