# Supplementary material for: Introducing and utilizing innovative technologies in health care systems: a country comparison for peripheral drug-eluting stents in Germany and the USA
Source: Front Public Health. 2025 Jun 19;13:1488091. doi: 10.3389/fpubh.2025.1488091 (PMC12222216; doi:10.3389/fpubh.2025.1488091)
Supplement: Supplementary file 1 [file Data_Sheet_1.zip › Supplement_Material/A.7_Exctraction_table_LoE_Ia_and_IIa_studies.docx]

**A.7 Extraction table: LoE Ia and IIa studies**

| **No.** | **Author (year)** | **Publ. date** (online, if earliest) | **Product name(s) of evaluated DES technologies** | **Study design** | **DES RCTs**  (study acronyms, if available) | **Follow-up** | | **Number of patients in DES studies** | **Reported endpoints** | | | | | | **Author assessment** |
| --- | --- | --- | --- | --- | --- | --- | --- | --- | --- | --- | --- | --- | --- | --- | --- |
|  |  |  |  |  |  | **Min.** | **Max.** |  | **M** | **UE** | **KM** | **QoL** | **SURE** | **Other** |  |
| **Studies of LoE Ia** | | | | | | | | | | | | | | | |
| Ia.1 | Jens et al. (2014) (1) | 2014 | ns | SR | Dake et al. (2011), Dake et al. (2013), VISTAR, SIROCCO I (2002), SIROCCO I (2006), SIROCCO II (2005), SIROCCO II (2006) | 1 month | 2 years | 828 | x | x | x |  |  |  | positive |
| Ia.2 | Katsanos et al. (2014) (2) | 2014 | ZILVER PTX | MA | SIROCCO (2006), ZILVER PTX (2012) | 3 years | 4 years | 572 |  |  | x |  | x | x | indecisive |
| Ia.3 | Baerlocher et al. (2015) (3) | 2015 | ns | MA | Bosiers et al. (2012), Dake et al. (2013), Dake et al. (2011), Duda et al. (2002), Duda et al. (2005), Duda et al. (2006), Falkowski et al. (2009), Rastan et al. (2012), Rastan et al. (2011), Scheinert et al. (2012), Siablis (2014), Tepe et al. (2010) | 6 months | 2 years | 1956 | x |  | x |  | x |  | positive |
| Ia.4 | Antonopoulos et al. (2017) (4) | 2017 | ZILVER PTX | MA | ZILVER PTX | 1 year | ns | 474 |  | x | x |  |  |  | indecisive |
| Ia.5 | He et al. (2019) (5) | 2019 | ZILVER PTX* | SR/MA | SIROCCO, Takashi et al. (2018), ZILVER PTX | 1 year | 2 years | 368 |  |  |  |  | x |  | neutral |
| Ia.6 | Zhou et al. (2020) (6) | 2019 | ZILVER PTX | SR/MA | Dake et al. (2011), Dake et al. (2013) | 1 year | 2 years | 122 | x |  | x |  | x |  | indecisive |
| Ia.7 | Dinh et al. (2021) (7) | 2021 | ZILVER PTX, Eluvia | SR/MA | BATTLE, DEBATE-ISR, Falkowski et al. (2020), FINN-PTX, SWEDEPAD, ZILVER PTX, ZILVERPASS | 1 year | 5 years | 1103 | x |  |  |  |  |  | neutral |
| Ia.8 | Zhang et al. (2021) (8) | 2021 | ZILVER PTX* | SR/MA | Bausback et al. (2019), DRASTICO, DEBATE-SFA, FINN-PTX, ZILVER PTX | 1 year | 7 years | 520 | x | x |  |  | x |  | indecisive |
| **Studies of LoE IIa** | | | | | | | | | | | | | | | |
| IIa.1 | Balk et al. (2008) (9) | 2008 | ns | HTA | Duda et al. (2006) | 2 years | 2 years | 74 | x |  | x |  |  | x | indecisive |
| IIa.2 | Marmagkiolis et al. (2014) (10) | 2014 | ZILVER PTX, S.M.A.R.T. | SR | SIROCCO, ZILVER PTX | 3 years | ns | 938 |  |  | x |  |  |  | indecisive |
| IIa.3 | Zechmeister-Kos et al. (2014) (11) | 2014 | ZILVER PTX | HTA | Dake et al. (2011), Dake et al. (2013), Duda et al. (2002), Duda et al. (2005) | 6 months | 2 years | 1122 | x | x | x | x | x |  | neutral |
| IIa.4 | Falkenberg et al. (2015) (12) | 2015 | ZILVER PTX, S.M.A.R.T. | HTA | Dake et al. (2011), Dake et al. (2013), Duda et al. (2005), Duda et al. (2006) | 6 months | 2 years | 1098 | x |  | x |  | x |  | indecisive |
| IIa.5 | Health Quality Ontario (2015) (13) | 2015 | ZILVER PTX | HTA | Dake et al. (2011), Dake et al. (2013) | 1 year | 2 years | 594 | x | x | x |  | x | x | positive |
| IIa.6 | Ho et al. (2017) (14) | 2017 | ZILVER PTX | SR | Zilver PTX | 1 year | 2 years | 144 |  |  |  |  |  | x | indecisive |
| IIa.7 | Jaff et al. (2017) (15) | 2017 | ZILVER PTX | MA | Zilver PTX | ns | 2 years | 1023 |  |  | x |  |  |  | indecisive |
| IIa.8 | Varetto et al. (2019) (16) | 2019 | ZILVER PTX, S.M.A.R.T. | SR/MA | Bausback et al. (2019), DEBATE-SFA, DRASTICO | ns | ns | ns |  |  |  |  | x | x | indecisive |
| IIa.9 | Nugraha et al. (2022) (17) | 2022 | ZILVER PTX | SR/MA | DRASTICO, REAL-PTX | ns | ns | 171 | x |  |  |  | x |  | indecisive |
| **Legend:** DES – drug-eluting stent; HTA – health technology assessment; MA – meta-analysis; ns – not stated; RCT – randomized controlled trial; SR – systematic review; * possibly more technologies focused, but not reported or not all sources available open access | | | | | | | | | | | | | | | |

**References**

1. Jens S, Conijn AP, Koelemay MJ, Bipat S, Reekers JA. Randomized trials for endovascular treatment of infrainguinal arterial disease: systematic review and meta-analysis (Part 1: above the knee). *European journal of vascular and endovascular surgery the official journal of the European Society for Vascular Surgery* (2014) **47**:524–35. doi:10.1016/j.ejvs.2014.02.011

2. Katsanos K, Spiliopoulos S, Karunanithy N, Krokidis M, Sabharwal T, Taylor P. Bayesian network meta-analysis of nitinol stents, covered stents, drug-eluting stents, and drug-coated balloons in the femoropopliteal artery. *Journal of Vascular Surgery* (2014) **59**:1123. doi:10.1016/j.jvs.2014.01.041

3. Baerlocher MO, Kennedy SA, Rajebi MR, Baerlocher FJ, Misra S, Liu D, et al. Meta-analysis of drug-eluting balloon angioplasty and drug-eluting stent placement for infrainguinal peripheral arterial disease. *Journal of Vascular and Interventional Radiology* (2015) **26**:459. doi:10.1016/j.jvir.2014.12.013

4. Antonopoulos CN, Mylonas SN, Moulakakis KG, Sergentanis TN, Sfyroeras GS, Lazaris AM, et al. A network meta-analysis of randomized controlled trials comparing treatment modalities for de novo superficial femoral artery occlusive lesions. *Journal of Vascular Surgery* (2017) **65**:234. doi:10.1016/j.jvs.2016.08.095

5. He R, Ye Y, Li Z, Jiang Y, Li Y, Liu Y, et al. Restenosis prevention with drug eluting or covered stents in femoropopliteal arterial occlusive disease: evidence from a comprehensive network meta-analysis. *European Journal of Vascular and Endovascular Surgery* (2019) **58**:61–74. doi:10.1016/j.ejvs.2018.12.020

6. Zhou Y, Zhang Z, Lin S, Xiao J, Ai W, Wang J, et al. Comparative efficacy and safety of endovascular treatment modalities for femoropopliteal artery lesions: a network meta-analysis of randomized controlled trials. *CardioVascular and Interventional Radiology* (2020) **43**:204–14. doi:10.1007/s00270-019-02332-4

7. Dinh K, Limmer AM, Chen AZ, Thomas SD, Holden A, Schneider PA, et al. Mortality rates after Paclitaxel-coated device use in patients with occlusive femoropopliteal disease: an updated systematic review and meta-analysis of randomized controlled trials. *Journal of Endovascular Therapy* (2021) **28**:755–77. doi:10.1177/15266028211023505

8. Zhang R, Ni L, Zeng R, Lai Z, Di X, Zhao Z, et al. An indirect comparison by Bayesian network meta-analysis of drug-coated devices versus saphenous vein graft bypass in femoropopliteal arterial occlusive disease. *Journal of Vascular Surgery* (2021) **74**:478-486.e11. doi:10.1016/j.jvs.2020.11.054

9. Balk E, Cepeda MS, Ip S, Trikalinos T, O'Donnell T. “Horizon scan of invasive interventions for lower extremity peripheral artery disease and systematic review of studies comparing stent placement to other interventions,”. In: *Horizon scan of invasive interventions for lower extremity peripheral artery disease and systematic review of studies comparing stent placement to other interventions*. Rockville (MD): Agency for Healthcare Research and Quality (US) (2008).

10. Marmagkiolis K, Hakeem A, Choksi N, Al-Hawwas M, Edupuganti MM, Leesar MA, et al. 12-month primary patency rates of contemporary endovascular device therapy for femoro-popliteal occlusive disease in 6,024 patients: beyond balloon angioplasty. *Catheterization and Cardiovascular Interventions* (2014) **84**:555–64. doi:10.1002/ccd.25510

11. Zechmeister-Koss I, Fischer S. *Drug-eluting stents for peripheral arterial occlusive disease [Medikamentenfreisetzende Stents bei peripherer arterieller Verschlusskrankheit]*. Decision support document 75. Wien (2014).

12. Falkenberg M, Carlson P, Nordanstig J, Pettersson J, Smidfelt K, Svanberg T, et al. *Drug eluting balloons and stents for symptomatic peripheral arterial disease*. Gothenburg: The Regional Health Technology Assessment Centre (HTA-centrum) Region Vastra Gotaland (2015).

13. Health Quality O. *Paclitaxel drug-eluting stents in peripheral arterial disease: a health technology assessment*. Toronto: Health Quality Ontario (2015).

14. Ho KJ, Owens CD. Diagnosis, classification, and treatment of femoropopliteal artery in-stent restenosis. *Journal of Vascular Surgery* (2017) **65**:545–57. doi:10.1016/j.jvs.2016.09.031

15. Jaff MR, Nelson T, Ferko N, Martinson M, Anderson LH, Hollmann S. Endovascular interventions for femoropopliteal peripheral artery disease: a network meta-analysis of current technologies. *Journal of Vascular and Interventional Radiology* (2017) **28**:1617. doi:10.1016/j.jvir.2017.08.003

16. Varetto G, Gibello L, Boero M, Frola E, Peretti T, Spalla F, et al. Angioplasty or bare metal stent versus drug-eluting endovascular treatment in femoropopliteal artery disease: a systematic review and meta-analysis. *Journal of Cardiovascular Surgery* (2019) **60**:546–56. doi:10.23736/S0021-9509.19.11115-9

17. Nugraha HG, Hilman S, Santiana L, Dewi DK, Raffaelo WM, Wibowo A, et al. Drug-coated balloon versus drug-eluting stent in patients with femoropopliteal artery disease: a systematic review and meta-analysis. *Vascular and Endovascular Surgery* (2022) **56**:385–92. doi:10.1177/15385744211051491
