# Supplementary material for: Introducing and utilizing innovative technologies in health care systems: a country comparison for peripheral drug-eluting stents in Germany and the USA
Source: Front Public Health. 2025 Jun 19;13:1488091. doi: 10.3389/fpubh.2025.1488091 (PMC12222216; doi:10.3389/fpubh.2025.1488091)
Supplement: Supplementary file 1 [file Data_Sheet_1.zip › Supplement_Material/A.8_HTA_reports_author_title_source.docx]

**A.8 HTA reports: author (year), title, and source**

| **Author (year)** | **Title** | **Source (URL), all last accessed: 10/20/2023** |
| --- | --- | --- |
| Cassese et al. (2013) | Paclitaxel-coated versus uncoated balloon angioplasty reduces target lesion revascularization in patients with femoropopliteal arterial disease: a meta-analysis of randomized trials* | <http://www.crd.york.ac.uk/CRDWeb/ShowRecord.asp?ID=12012055622> |
| Zechmeister-Koss et al. (2014) | Drug-eluting stents for peripheral arterial occlusive disease [Medikamentenfreisetzende Stents bei peripherer arterieller Verschlusskrankheit]** | <https://eprints.aihta.at/1032/> |
| Health Quality Ontario (2015) | Paclitaxel drug-eluting stents in peripheral arterial disease: a health technology assessment** | <http://www.crd.york.ac.uk/CRDWeb/ShowRecord.asp?ID=32016000197> |
| Health Quality Ontario (2015) | Paclitaxel drug-eluting stents in peripheral arterial disease: OHTAC recommendation*** | <http://www.crd.york.ac.uk/CRDWeb/ShowRecord.asp?ID=32016000196> |
| **Legend:** HTA – health technology assessment, OHTAC – Ontario Health Technology Advisory Committee, * considered not relevant after full text screening, ** identified in initial database literature search, *** no full text available | | |
