# Supplementary material for: Introducing and utilizing innovative technologies in health care systems: a country comparison for peripheral drug-eluting stents in Germany and the USA
Source: Front Public Health. 2025 Jun 19;13:1488091. doi: 10.3389/fpubh.2025.1488091 (PMC12222216; doi:10.3389/fpubh.2025.1488091)
Supplement: Supplementary file 1 [file Data_Sheet_1.zip › Supplement_Material/A.9_RCT_assessment_RoB.docx]

**A.9 RCT assessment: potential risk of bias and short description of assessment**

| **Study acronym  (publication / citation details)** | **Assessment domains (1.-5.)** | | | | | | **Potential risk of bias at study level*** |
| --- | --- | --- | --- | --- | --- | --- | --- |
|  | **1.  Adequate generation of randomization sequence** | **2. Concealment of group allocation** | **3. Blinding** | | **4.  Outcome- independent  reporting of all relevant  endpoints** | **5.  Absence of other  aspects** |  |
|  |  |  | **Patient** | **Treating or further treating persons** |  |  |  |
| **SIROCCO I** (Duda et al., 2002^A^ & 2006^B^) | unclear | unclear | yes | unclear | no | yes | **high** |
| **SIROCCO II** (Duda et al., 2005 & 2006^B^) | unclear | unclear | yes | unclear | no | yes | **high** |
| **ZILVER PTX** (Dake et al., 2011, 2013^C^, 2016 & 2020) | yes | yes | no | unclear | unclear | yes | **high** |
| **Miki et al.**, 2016 | unclear | unclear | yes | yes | unclear | yes | **high** |
| **REAL PTX** (Backhaus et al., 2019) | yes | unclear | unclear | unclear | yes | no | **high** |
| **DRASTICO** (Liistro et al. 2019) | yes | unclear | yes | unclear | yes | no | low |
| **ZILVERPASS** (Bosiers et al., 2020) | yes | yes | unclear | no | yes | yes | low |
| **Falkowski et al.**, 2020 | yes | yes | yes | unclear | unclear | no | low |
| **BATTLE** (Goueffic et al., 2020) | yes | yes | yes | no/yes^D^ | yes | no | low |
| **Legend:** ^A^ Publication was not found in the systematic literature search but was identified afterwards; ^B^ it is the same publication; ^C^ publication is assigned level of evidence (LoE) III; ^D^ treating physicians were not blinded but further personnel (incl. data reviewers/analysts) was blinded; result per assessment domain: yes, no, unclear; * bias assessment result: "low" – assessment of "yes" in all domains, or in some domains and situation-specific assessment of "unclear" or "no" in some domains expect domain 2. / bias assessment result: "high" – assessment of "unclear" or "no" at least in domain 2., and situation-specific assessment of "unclear" or "no" in domain 4. The classification of bias potential of the studies’ results considers the individual assessment of domains 1.-5. A "high" risk of bias means that studies’ results would change in their basic statement if the bias aspects were eliminated (1, 2). | | | | | | | |

**Short description of RCT assessment of risk of bias (RoB) potential:**

One of the 10 RCTs had to be downgraded to level of evidence (LoE) IIb after determining the potential RoB, as the assessment criterion concealment of group allocation was not reported in the corresponding article (3). According to our assessment, most of the remaining n=9 RCTs had a high RoB (n=5). Trials with a high RoB are SIROCCO I (4, 5), SIROCCO II (5, 6), REAL PTX (7), DRASTICO (8), and the RCT presented in the article by Miki et al. (2016) (9). In these five RCTs, the concealment of the group allocation is not clearly reported and therefore is assessed as "unclear". In the articles on the SIROCCO I & II trials, an outcome-independent reporting of all relevant endpoints is missing (assessment "no"), and in the article on REAL PTX, explanations on blinding of patients, and treating or further treating persons are only reported "unclear" (7). The RCTs ZILVER PTX (10–13), ZILVERPASS (14), BATTLE (15), and the one described in Falkowski et al. (2020) (16) show a low potential of RoB according to our assessment. For example, the articles on all these trials report an adequate generation of the randomization sequence and the concealment of group allocation. However, for none of the trials all RoB criteria can be considered as fulfilled (assessment "yes").

**References**

1. Federal Joint Committee (Gemeinsamer Bundesausschuss). *Rules of Procedure (VerfO) of the Federal Joint Committee (G-BA) in the version of March 6, 2019: in the version of December 18, 2008 published in the Federal Gazette No. 84a (Supplement) of June 10, 2009 entered into force on April 1, 2009 last amended on August 16, 2018 published in the Federal Gazette BAnz AT 05.03.2019 B2 entered into force on March 6, 2019 [Verfahrensordnung (VerfO) des Gemeinsamen Bundesausschusses (G-BA) in der Version vom 06.03.2019: in der Fassung vom 18. Dezember 2008 veröffentlicht im Bundesanzeiger Nr. 84a (Beilage) vom 10. Juni 2009 in Kraft getreten am 1. April 2009 zuletzt geändert am 16. August 2018 veröffentlicht im Bundesanzeiger BAnz AT 05.03.2019 B2 in Kraft getreten am 6. März 2019]* (2019) [cited 2024 Apr 10]. Available from: https://www.g-ba.de/richtlinien/42/

2. Institute for Quality and Efficiency in Health Care [Institut für Qualität und Wirtschaftlichkeit im Gesundheitswesen]. *General methods: Version 5.0, Institute for Quality and Efficiency in Health Care (IQWiG), Cologne, Germany [Allgemeine Methoden: Version 5.0, Institut für Qualität und Wirtschaftlichkeit im Gesundheitswesen (IQWiG), Köln]* (2017) [cited 2024 Apr 10]. Available from: https://www.iqwig.de/methoden/allgemeine-methoden_version-5-0.pdf

3. Björkman P, Auvinen T, Hakovirta H, Romsi P, Turtiainen J, Manninen H, et al. Drug-eluting stent shows similar patency results as prosthetic bypass in patients with femoropopliteal occlusion in a randomized trial. *European Journal of Vascular and Endovascular Surgery* (2019) **58**:e353‐e354. doi:10.1016/j.ejvs.2019.06.980

4. Duda SH, Pusich B, Richter G, Landwehr P, Oliva VL, Tielbeek A, et al. Sirolimus-eluting stents for the treatment of obstructive superficial femoral artery disease: six-month results. *Circulation* (2002) **106**:1505–9. doi:10.1161/01.cir.0000029746.10018.36

5. Duda SH, Bosiers M, Lammer J, Scheinert D, Zeller T, Oliva V, et al. Drug-eluting and bare nitinol stents for the treatment of atherosclerotic lesions in the superficial femoral artery: long-term results from the SIROCCO trial. *Journal of Endovascular Therapy* (2006) **13**:701–10. doi:10.1583/05-1704.1

6. Duda SH, Bosiers M, Lammer J, Scheinert D, Zeller T, Tielbeek A, et al. Sirolimus-eluting versus bare nitinol stent for obstructive superficial femoral artery disease: the SIROCCO II trial. *Journal of Vascular and Interventional Radiology* (2005) **16**:331–8. doi:10.1097/01.RVI.0000151260.74519.CA

7. Bausback Y, Wittig T, Schmidt A, Zeller T, Bosiers M, Peeters P, et al. Drug-eluting stent versus drug-coated balloon revascularization in patients with femoropopliteal arterial disease. *Journal of the American College of Cardiology* (2019) **73**:667–79. doi:10.1016/j.jacc.2018.11.039

8. Liistro F, Angioli P, Porto I, Ducci K, Falsini G, Ventoruzzo G, et al. Drug-eluting balloon versus drug-eluting stent for complex femoropopliteal arterial lesions: the DRASTICO study. *Journal of the American College of Cardiology* (2019) **74**:205–15. doi:10.1016/j.jacc.2019.04.057

9. Miki K, Fujii K, Shibuya M, Fukunaga M, Imanaka T, Tamaru H, et al. Comparing the vascular response in implantation of self-expanding, bare metal nitinol stents or Paclitaxel-eluting nitinol stents in superficial femoral artery lesions: a serial optical frequency domain imaging study. *EuroIntervention* (2016) **12**:1551–8. doi:10.4244/EIJ-D-15-00399

10. Dake MD, Ansel GM, Jaff MR, Ohki T, Saxon RR, Smouse HB, et al. Paclitaxel-eluting stents show superiority to balloon angioplasty and bare metal stents in femoropopliteal disease: twelve-month Zilver PTX randomized study results. *Circulation: Cardiovascular Interventions* (2011) **4**:495–504. doi:10.1161/CIRCINTERVENTIONS.111.962324

11. Dake MD, Ansel GM, Jaff MR, Ohki T, Saxon RR, Smouse HB, et al. Sustained safety and effectiveness of Paclitaxel-eluting stents for femoropopliteal lesions: 2-year follow-up from the Zilver PTX randomized and single-arm clinical studies. *Journal of the American College of Cardiology* (2013) **61**:2417–27. doi:10.1016/j.jacc.2013.03.034

12. Dake MD, Ansel GM, Jaff MR, Ohki T, Saxon RR, Smouse HB, et al. Durable clinical effectiveness with Paclitaxel-eluting stents in the femoropopliteal artery: 5-year results of the Zilver PTX randomized trial. *Circulation* (2016) **133**:1472–83. doi:10.1161/CIRCULATIONAHA.115.016900

13. Dake MD, Ansel GM, Bosiers M, Holden A, Iida O, Jaff MR, et al. Paclitaxel-coated Zilver PTX drug-eluting stent treatment does not result in increased long-term all-cause mortality compared to uncoated devices. *CardioVascular and Interventional Radiology* (2020) **43**:8–19. doi:10.1007/s00270-019-02324-4

14. Bosiers M, Setacci C, Donato G de, Torsello G, Silveira PG, Deloose K, et al. ZILVERPASS study: Zilver PTX stent vs bypass surgery in femoropopliteal lesions. *Journal of Endovascular Therapy* (2020) **27**:287–95. doi:10.1177/1526602820902014

15. Gouëffic Y, Sauguet A, Desgranges P, Feugier P, Rosset E, Ducasse E, et al. A polymer-free Paclitaxel-eluting stent versus a bare-metal stent for de novo femoropopliteal lesions: the BATTLE trial. *JACC. Cardiovascular interventions* (2020) **13**:447–57. doi:10.1016/j.jcin.2019.12.028

16. Falkowski A, Bogacki H, Szemitko M. Assessment of mortality and factors affecting outcome of use of Paclitaxel-coated stents and bare metal stents in femoropopliteal pad. *Journal of clinical medicine* (2020) **9**:1–11.
