# Supplementary figures and images for: Introducing and utilizing innovative technologies in health care systems: a country comparison for peripheral drug-eluting stents in Germany and the USA
Source: Front Public Health. 2025 Jun 19;13:1488091. doi: 10.3389/fpubh.2025.1488091 (PMC12222216; doi:10.3389/fpubh.2025.1488091)

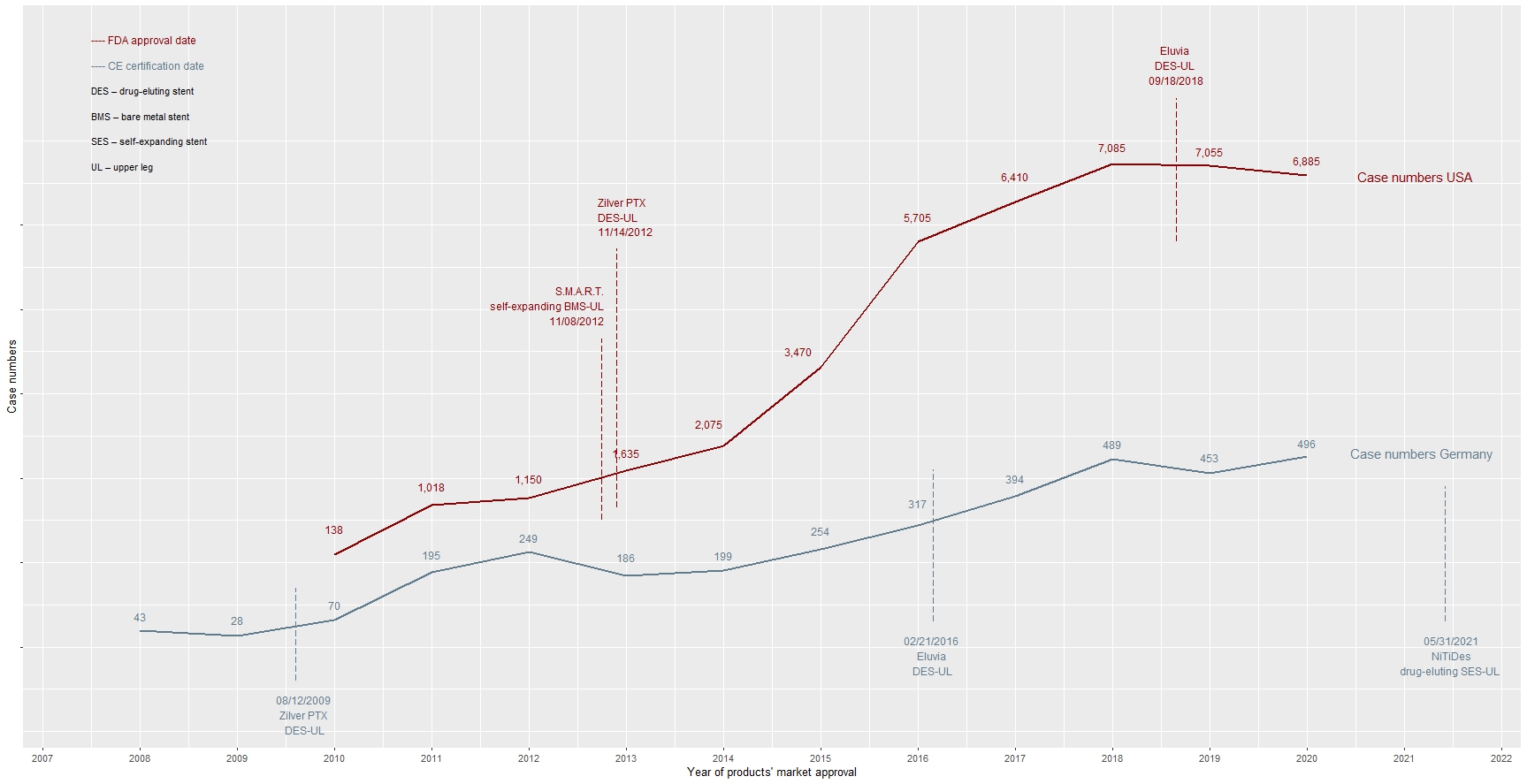

Supplement: Supplementary file 1 [file Data_Sheet_1.zip › Supplement_Material/A.14_Market_approval_development_graphical_representation.jpeg]

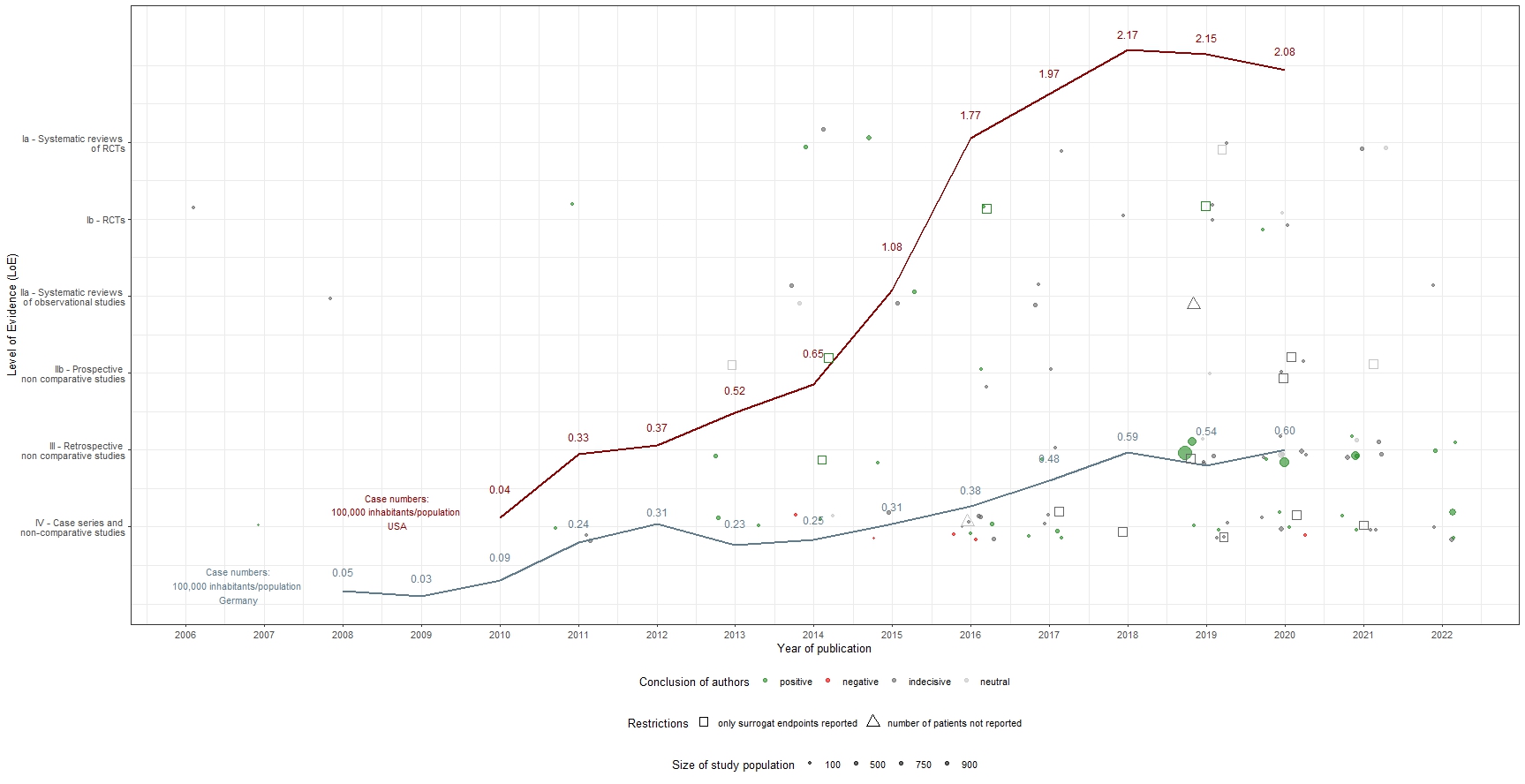

Supplement: Supplementary file 1 [file Data_Sheet_1.zip › Supplement_Material/A.17_Standardization_graphical_representation.jpeg]
